# Correction: Cold exposure protects against medial arterial calcification development via autophagy

**DOI:** 10.1186/s12951-023-02047-2

**Published:** 2023-08-29

**Authors:** Fu-Xing-Zi Li, Jun-Jie Liu, Feng Xu, Su-Kang Shan, Ming-Hui Zheng, Li-Min Lei, Xiao Lin, Bei Guo, Chang-Chun Li, Feng Wu, Ke-Xin Tang, Ye-Chi Cao, Yun-Yun Wu, Jia-Yue Duan, Yan-Lin Wu, Si-Yang He, Xi Chen, Ling-Qing Yuan

**Affiliations:** 1 Department of Metabolism and Endocrinology, National Clinical Research Center for Metabolic Disease, The Second Xiangya Hospital, Central South University, Changsha, Hunan 410011 China; 2 Department of Periodontal Division, Hunan Xiangya Stomatological Hospital, Central South University, Changsha, China; 3 Department of Radiology, The Second Xiangya Hospital, Central South University, Changsha, China; 4 Department of Pathology, The Second Xiangya Hospital, Central South University, Changsha, China

**Correction: Journal of Nanobiotechnology (2023) 21:226**

10.1186/s12951-023-01985-1

Following publication of the original article [1], details for affiliation 1 were incorrectly given as “Department of Metabolism and Endocrinology, National Clinical Research Center, The Second Xiangya Hospita for Metabolic Disease, Central South University, Changsha, China”, but should have been “Department of Metabolism and Endocrinology, National Clinical Research Center for Metabolic Disease, ​The Second Xiangya Hospital, Central South University, Changsha, Hunan, 410011, China.”

The original article [1] has been corrected.
