# Supplementary material for: Cold exposure protects against medial arterial calcification development via autophagy
Source: J Nanobiotechnology. 2023 Jul 17;21:226. doi: 10.1186/s12951-023-01985-1 (PMC10351118; doi:10.1186/s12951-023-01985-1)
Supplement: Supplementary file 1 — Supplementary Material 1 [file 12951_2023_1985_MOESM1_ESM.pdf]

Cold Exposure Protects Against Medial Arterial Calcification Development via Autophagy

Fu-Xing-Zi Li[1], Jun-Jie Liu[2], Feng Xu[1], Su-Kang Shan[1], Ming-Hui Zheng[1], Li-Min Lei[1], Xiao Lin[3], Bei Guo[1], Chang-Chun Li[1], Feng Wu[4], Ke-Xin Tang[1], Ye-Chi Cao[1], Yun-Yun Wu[1], Jia-Yue Duan[1], Yan-Lin Wu[1], Si-Yang He[1], Xi Chen[1] and Ling-Qing Yuan[1]

[1]Department of Metabolism and Endocrinology, National Clinical Research Center for Metabolic Disease, Hunan Provincial Key Laboratory of Metabolic Bone Diseases, The Second Xiangya Hospital, Central South University, Changsha, China.
[2]Department of Periodontal Division, Hunan Xiangya Stomatological Hospital, Central South University, Changsha, China.
[3]Department of Radiology, The Second Xiangya Hospital, Central South University, Changsha, China.
[4]Department of Pathology, The Second Xiangya Hospital, Central South University, Changsha, China.

Abstract

Medial arterial calcification (MAC), a systemic vascular disease different from atherosclerosis, is associated with an increased incidence of cardiovascular events. Several studies have demonstrated that ambient temperature is one of the most important factors affecting cardiovascular events. However, there has been limited research on the effect of different ambient temperatures on MAC. In the present study, we showed that cold temperature exposure (CT) in mice slowed down the formation of vitamin D (VD)-induced vascular calcification compared with room temperature exposure (RT). To investigate the mechanism involved, we isolated plasma-derived exosomes from mice subjected to CT or RT for 30 days (CT-Exo or RT-Exo, respectively). Compared with RT-Exo, CT-Exo remarkably alleviated the calcification/senescence formation of vascular smooth muscle cells (VSMCs) and promoted autophagy by activating the phosphorylation of AMP-activated protein kinase (p-AMPK) and inhibiting phosphorylation of mammalian target of rapamycin (p-mTOR). At the same time, CT-Exo promoted autophagy in β-glycerophosphate (β-GP)-induced VSMCs. The number of autophagosomes and the expression of autophagy-related proteins ATG5 and LC3B increased, while the expression of p62 decreased. Based on a microRNA chip microarray assay and real-time polymerase chain reaction, miR-320a-3p was highly enriched in CT-Exo as well as thoracic aortic vessels in CT mice. miR-320a-3p downregulation in CT-Exo using AntagomiR-320a-3p inhibited autophagy and blunted its anti-calcification protective effect on VSMCs. Moreover, we identified that programmed cell death 4 (PDCD4) is a target of miR-320a-3p, and silencing PDCD4 increased autophagy and decreased calcification in VSMCs. Treatment with CT-Exo alleviated the formation of MAC in VD-treated mice, while these effects were partially reversed by GW4869. Furthermore, the anti-arterial calcification protective effects of CT-Exo were largely abolished by AntagomiR-320a-3p in VD-induced mice. In summary, we have highlighted that prolonged cold may be a good way to reduce the incidence of MAC. Specifically, miR-320a-3p from CT-Exo

could protect against the initiation and progression of MAC via the AMPK/mTOR autophagy pathway.

Keywords: Cold exposure, Arterial calcification, Plasma-derived exosomes, Autophagy, Senescence, miR-320a-3p, PDCD4.

Graphic Abstract

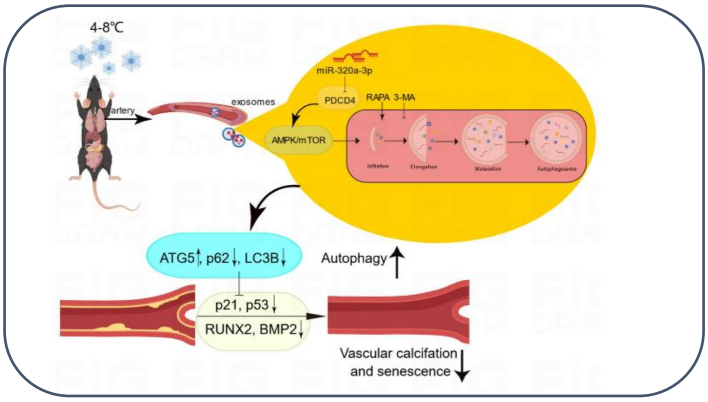

Introduction

The benefits of outdoor swimming in the winter and cold bathing are well known. Indeed, the physiological response of humans to cold environments has been studied for a long time. So-called cold exposure refers to the direct exposure of the human body to an environment lower than normal temperature (20°C). In a cold environment, the human body can produce a series of physiological reactions, but no definitive conclusion has been reached because this special environment has many influences on the human body, and the individual responses to the cold environment are also different. Researchers have shown that cold exposure can affect the activities of the nervous[1], cardiovascular[2, 3], musculoskeletal[4, 5], immune[6] and endocrine systems[7]. Cold environments induce long-term effects that increase the risk of cardiovascular disease (CVD) morbidity and mortality[8]. However, no studies have been reported on the effect of cold environments on the development of medial arterial calcification (MAC).

The founder of modern medicine, William Osler, once put forward the view of ‘vascular ageing, a man is as old as his arteries’, revealing the important connection between vascular ageing and individual ageing[9]. MAC is an important part of vascular ageing. It is a systemic vascular disease that is distinct from atherosclerosis and is commonly seen in diabetes, end stage renal disease and ageing, resulting in increased vascular stiffness[10, 11], diastolic heart failure[12], impaired coronary perfusion[13] and chronic limb ischaemia[14]. MAC was previously thought to be a simple passive deposition of calcium and phosphorus. However, researchers have paid more attention to the pathogenesis of arterial calcification since the discovery of bone morphogenetic protein (BMP) in tissue with MAC[15-19]. Nonetheless, the pathogenesis of MAC has not been fully elucidated – except for the pathogenesis of arterial calcification caused by a single gene mutation, which has been clearly studied – and there is a lack of treatment for the disease.

According to MISEV 2018, extracellular vesicles (EVs) can be divided into two subgroups: small EVs (sEVs or exosomes, < 100 nm or < 200 nm) and medium/large EVs (m/lEVs, > 200 nm)[20]. Exosomes are membranous vesicles secreted by cells, usually 50–150 nm in diameter, which are widely present in various body fluids and carry lipids, proteins, messenger RNAs (mRNAs), microRNAs (miRNAs), non-coding RNAs (ncRNAs) and other important biological function molecules[21-25]. Calcification of the major arteries is an important phenotype of vascular ageing. Researchers have found that exosomes play different roles in MAC[26-29]. Thus, we hypothesis exosomes may serve as communication vesicles and mediate vascular calcification at an ambient temperature.

Autophagy is associated with many physiological and pathological processes, such as development, differentiation, neurodegenerative diseases[30, 31], stress[32], infection[33] and cancer[34]. Mammalian target of rapamycin (RAPA) (mTOR) is an important kinase that regulates the induction of autophagy. Activated mTOR acts via AKT and mitogen-activated protein kinase (MAPK) signalling to inhibit autophagy, while adenosine monophosphate–activated kinase (AMPK) and p53 signalling negatively regulate mTOR to promote autophagy. Studies have shown that autophagy is particularly closely related to ageing[35]. Cell ageing and autophagy have a common regulatory pathway that involves key proteins such as mTOR, SIRTL and p53. With ageing, cellular senescence is usually accompanied by a decrease in the level of autophagy as well as the degradation of damaged organelles and proteins; the decrease in the level of autophagy can accelerate the ageing process[13, 36]. Multiple studies have shown that autophagy occurs in the context of atherosclerosis[37-39] and hypertension[40]. Evidence suggests that RAPA, an inducer of autophagy, prevents phenotypic switching and the hyperproliferation of vascular smooth muscle cells (VSMCs)[41]. Therefore, autophagy may act as an endogenous protective mechanism to alleviate calcification in VSMCs[42]. These phenomena suggest that autophagy plays a key role in arterial calcification.

In the present study, we hypothesised that plasma-derived exosomes isolated from mice subjected to cold temperature exposure (CT-Exo) protect against the calcification and senescence of the aortic media by regulating the level of autophagy. We thoroughly explored the effects of CT on the pathogenesis of MAC and clarified its mechanism, investigating whether cold temperature exposure (CT) can protect against MAC, whether autophagy is involved in arterial calcification during CT and whether plasma-derived exosomes play a protective role by regulating autophagy. Our findings might provide new ideas and new ways to explore the pathogenesis and prevention of MAC.

Methods and materials

Cell Culture

VSMCs were purchased from the National Platform of Experimental Cell Resources for SciTech (Beijing, China). They were incubated in Dulbecco's Modified Eagle's Medium (DMEM) (Gibco, Grand Island, NY, USA) with 10% foetal bovine serum (FBS; Gibco) and 1% penicillin-streptomycin (P1400, Solarbio, Beijing, China). The

culture medium was refreshed every 3 days and the cells were cultured at 37°C with a humidified atmosphere of 5% CO_2. To induce calcification, VSMCs were cultured in a medium containing 10 mM β-glycerophosphate (β-GP; 50020, Sigma-Aldrich, St. Louis, MO, USA) to induce the osteoblastic differentiation of VSMCs. To reveal the effect of exosomes isolated from mice subjected to room temperature exposure (RT-Exo) or CT-Exo on the osteoblastic differentiation of VSMCs and the mechanism involved, VSMCs were incubated with 200 ng/μL of CT-Exo or RT-Exo in subsequent experiments. To investigate the effect of autophagy on VSMC calcification, cells were pre-treated with 5 mM of the autophagy inhibitor 3-MA (5142-23-4; SelleckChemm, USA) or 1 μM of the autophagy inducer RAPA (53123-88-9; SelleckChem) for 30 min. The cells were treated with β-GP for various times and then collected for different experiments: after 3 days, cells were collected for western blotting; after 10 days, cells were collected for senescence-associated β-galactosidase (SA-β-gal) staining (C0602; Beyotime Institute of Biotechnology, Shanghai, China); after 14 days, cells were collected for alkaline phosphatase (ALP) activity detection (A059-1-1; Nanjing Jiancheng Bioengineering Institute, Nanjing, China) and ALP staining (Solarbio); and after 28 days, cells were collected for ARS staining (G1038; Servicebio, Wuhan, China).Agonists and inhibitors of the AMPK/mTOR signalling pathway were used to investigate its role in calcification. VSMCs were stimulated with 10 μM of Compound C (S7306; SelleckChem) or 10 μM of MHY1485 (S7811; SelleckChem) for 30 min and then treated with 200 ng/μL of CT-Exo for 48 h. p-AMPK, t-AMPK, p-mTOR, t-mTOR and RUNX2 protein expression was evaluated in the cell lysates. The SA-β-gal and ARS staining was the same as described above; CT-Exo, Compound C and MHY1485 were changed once every 3 days for a period of 10 or 28 days, respectively.

Plasma Collection and Administration

CT plasma or CT-Exo^{free} plasma was isolated from mice subjected to CT for 30 days (4–8°C). CT-Exo^{free} plasma was produced as follows: CT plasma was diluted with PBS (1:4, v/v), and then ultracentrifuged at 100,000 *g* for 18 h to collect the supernatant. After centrifugation, the exosomes were concentrated at the bottom of the test tube and about 80% of the upper plasma had been collected, CT-Exo^{free} plasma was filtered by 0.22 μm filter and centrifuged at 4,000 *g* to approximately the initial plasma volume by ultrafiltration in a 15 mL Amicon Ultra-15 centrifugal filter unit (Millipore, Billerica, MA, USA). The exosomes were stored at -80°C before use.

Six-week-old male mice (n = 6) were systemically treated with phosphate-buffered saline (PBS), CT plasma or CT-Exo^{free} plasma (100 μL/injection) via tail intravenous injection 8 times over 24 days (From 0 day to 24th day)[43]. On the 14th day, the mice were intraperitoneally injected with vitamin D (VD) for 5 consecutive days and mice were sacrificed after waiting for another week of PBS, CT plasma or CT-Exo^{free} plasma treatment.

Isolation and Identification of Exosomes

Plasma samples were obtained from RT mice (kept at 22–25°C) or CT mice (kept at 4–8°C) for 30 days. Briefly, we collected the whole blood of mice using cardiac blood collection technology into Eppendorf (EP) tubes containing Ethylene Diamine Tetra

Acetic Acid (EDTA) anticoagulant. Blood samples were processed within 30 min of collection. The mixture was centrifuged to collect the plasma at 3,000 *g* for 20 min. Subsequently, the plasma underwent successive centrifugation at 3,000 *g* for 20 min and then 10,000 *g* for 30 min to discard dead cells and cellular debris. Then supernatant was collected, supernatant:PBS=1:4 Plasma+PBS suspension was added to the ultra-high centrifuge tube. The final supernatant was ultracentrifuged at 100,000 *g* for 120 min. The supernatant was removed, with 500 μL left at the bottom and then 11 mL PBS was added to resuspend, before being ultracentrifuged again at 100,000 *g* for 120 min (avoiding freeze-thaw cycles) and then re-suspended in 15 mL of PBS. The suspension was filtered through a 0.22 μm filter steriliser (Millipore) and centrifuged at 4,000 *g* to approximately 200 μL by ultrafiltration in a 15 mL Amicon Ultra-15 centrifugal filter unit (Millipore). All procedures were performed at 4°C. Exosomes were stored at -80°C or used for the downstream experiments.

The exosomal protein content was quantified with the BCA protein assay kit (P0012; Beyotime). Transmission electron microscopy (TEM; H-7650, Hitachi, Tokyo, Japan) and dynamic light scattering (DLS) with a Nanosizer™ instrument (Malvern Instruments, Malvern, UK) were used to observe the morphology and measure the size distribution of exosomes, respectively. The protein expression of exosomal markers (TSG101, CD81 and CD9) was assessed by western blotting.

For *in vitro* assays, exosomes in different groups were used at a concentration of 200 ng/μL. For *in vivo* experiments, exosomes were used at 200 μg (dissolved in 100 μL PBS for intravenous injection) per time and per mouse.

TEM

VSMCs were fixed overnight in 2.5% glutaraldehyde and post-fixed in 1% osmic acid for 2 h. The samples were then dehydrated, embedded and sectioned. After being double stained with 3% uranyl acetate and lead nitrate, the autophagic structures in the cells were viewed using a TEM (H-7650, Hitachi, Tokyo, Japan).

Exosome Uptake Assay and Tracing

In vitro, CT-Exo were labelled with PKH26 red fluorescent dye (MINI26-1KT, Sigma-Aldrich) according to the manufacturer's protocol. After removing the unbound dye, CT-Exo were added to the VSMCs and incubated at 37°C for 6 h. After discarding the culture supernatant and washing the cells with PBS, the cells were fixed with 4% paraformaldehyde (PFA) for 15 min and then incubated with DAPI (C0065; Solarbio) to stain the nuclei. The uptake of the red PKH26-labeled CT-Exo by VSMCs was determined with a fluorescence microscope (Nikon Instruments Korea, Seoul, Korea).

In vivo, to explore whether CT-Exo could be transported from bone to blood vessel walls after intramedullary injection, 100 μL of 1 μg/μL CT-Exo was labelled with 5 μL of 200 μg/mL 1,1′-dioctadecyl-3,3,3′,3′-tetramethylindotricarbocyanine iodide (DiR; 2024243, Invitrogen, Carlsbad, CA, USA) according to the manufacturer's instructions. Then, the same was ultracentrifuged to remove unbound dye. Mice were injected with DiR-labelled CT-Exo via the tail vein injection for 3 consecutive days. Live imaging was performed 24 h after the last injection. The mice were killed, organs removed for

imaging, the thoracic aorta of the mice was dissected and immunofluorescence staining was performed on quick frozen sections to analyse the uptake of exosomes in arterial vessels. An anti-TSG101 antibody (1:250, bs-1365R, Bioss, Beijing, China) was used to label exosomes.

Measurement of Reactive Oxygen Species (ROS) Generation

Intracellular ROS production was measured by flow cytometry using the cell-permeable fluorogenic probe DCFH-DA (S0033S; Beyotime) according to the manufacturer's instructions. Briefly, calcified VSMCs were treated with 200 ng/μL of CT-Exo or PBS for 6 days, washed three times with PBS and then incubated with 1×10^{-5} μM DCFH-DA at 37°C for 20 min.

Apoptosis Assay

VSMCs were treated with CT-Exo or PBS with or without β-GP for 3 days. Apoptosis was measured using the Annexin V-FITC/PI Detection Kit (556547, BD Bioscience, USA) according to the manufacturer's protocol. For Annexin V-FITC/PI staining, the treated cells were harvested, washed twice with PBS and resuspended in 300 μL of 1× binding buffer, at room temperature in the dark, followed by incubation with 5 μL of Annexin V-FITC for 15 min and 10 μL of PI solution for 5 min. Next, the cell suspension was diluted with 200 μL of annexin V binding buffer and analysed by flow cytometry.

Animal Study

Mice were housed in the Animal House of the Second Xiangya Hospital with a 12-h photoperiod. All experiments were started on 7–8 week old mice. Mice were placed in RT (22–25°C) or CT (4–8°C) environments, and their hair changes, mental state and activity were observed. Their body mass was measured and recorded at regular intervals every week. On the 30th day after modelling, blood was taken to measure ALT levels. Mice were shaved to observe whether their skin was frostbitten, important organs were collected for photography and the mass of the heart, liver, spleen, lung, and kidney tissues was measured. The organ indices and lung wet/dry weight of the mice were calculated.

Mice were injected intraperitoneally with VD (500 U/g/day) for 5 days to induce arterial calcification and ageing. Mice were fed with regular chow throughout the entire experiments. The RT mice were kept at 22–25°C for 30 days. The CT mice were first kept at 18°C for 7 days (for adaptation) and then kept at 4–8°C for another 30 days. The 4–8°C cold room was equipped with a ventilation system that allowed cold air to circulate.

After 30 days of RT or CT, the mice were administered a high-dose of VD for 5 consecutive days, followed by waiting for 7 days. This treatment occurred at either RT or CT, depending on the initial 30-day treatment. All live mice (n = 6) were sacrificed via the intraperitoneal injection of sodium pentobarbital (50 mg/kg) followed by cervical dislocation. Blood samples were collected to detect the levels of aminotransferase (ALT), using an automatic biochemical analyser (Chemray 800; Redu

Life Technology, Shenzhen, China). The thoracic aorta was embedded in paraffin, sectioned and then stained with ARS. The artery from the aortic arch to the iliac branch was isolated for the determination of arterial wall calcium content. No mice died during the experiment.

In another experiment, CT mice were injected intraperitoneally with GW4869 (2 mg/kg; S7609, SelleckChem) to inhibit circulating exosomes[44, 45]. Immunohistochemistry was carried out to determine RUNX2 expression in aortic tissues. ARS staining were used to detect MAC. Finally, the calcium content was measured.The impact of CT-Exo and RT-Exo on acute arterial calcification and the role of miR-320a-3p in the CT-Exo-induced alleviation of arterial calcification were also evaluated. Mice were injected intravenously with 200 μg of CT-Exo, AntagomiR-320a-3p or AntagomiR-NC-pre-treated CT-Exo, or an equal volume of PBS (100 μL per mouse) every 3 days until the end of the experiment (n = 6 per group). At the same time, the mice were injected with VD for 5 consecutive days, followed by waiting for 7 days. Blood samples were collected to detect the levels of blood urea nitrogen (BUN), creatinine (CREA), calcium, and phosphorus using an automatic biochemical analyser. The thoracic aortas were dissected. Immunohistochemistry was carried out to determine the levels of RUNX2 in aortic tissues. ARS or Von Kossa staining (G1043; Servicebio) was used to detect artery calcification. Finally, the calcium content was measured.

To explore whether miR-320a-3p was the only effective component in CT-Exo, we intravenously injected 200μg CT-Exo, 5mg/kg AgomiR-320a-3p, 5mg/kg AgomiR NC, or equivalent volume of PBS (100 per mouse) into mice every 3 days until the end of the experiment (n = 6 per group). Meanwhile, mice were continuously injected with VD for 5 days and then waited for 7 days. The thoracic aorta was dissected, ARS staining was performed to detect the content of mineralised nodules in the arteries and calcium content was measured. Immunohistochemical detection of RUNX2 levels was performed on the aortic mesomembrane.

Next, whether CT-Exo exerts an inhibitory effect on MAC *in vivo* through the autophagy pathway was investigated. The mice were randomly divided into six groups (n = 6 per group): PBS (CTRL), VD+PBS (PS), VD+CT-Exo (CT-Exo), VD+3-MA (3-MA), VD+RAPA (RAPA) and VD+CT-Exo+3-MA (CT-Exo+3-MA). Mice were intraperitoneally injected with either 3-MA (15 mg/kg) or RAPA (2mg/kg) starting 5 days before the first CT-Exo injection (CT-Exo was injected every 3 days for a total of eight injections) until the experiment was terminated. Then, arterial calcification was induced by VD 2 weeks before the mice were sacrificed. One mouse from the CT-Exo+3-MA group and the RAPA group died from unknown causes after being treated four times. Immunohistochemistry was carried out to determine p21 expression in aortic tissues. MAC was detected by ARS and Von Kossa staining and the calcium content was measured.

Quantitative Real-Time Polymerase Chain Reaction (qRT-PCR)

Total RNA was isolated from cells with TRIzol Reagent (Invitrogen) based on the manufacturer's instructions[46]. For miRNA detection, miRNA was reverse transcribed and analysed by TB Green® Premix Ex Taq™ II (Tli RNaseH Plus; RR820A, Takara,

Kyoto, Japan) based on the manufacturer's protocol and using U6 as the normalisation control. U6 (HmiRQP9001) and miR-320a-3p (HmiRQP0405) primers were purchased from GeneCopoeia (Guangzhou, China).

RNA Sequencing

The RT-Exo and CT-Exo groups were selected for RNA sequencing (n = 3 per group). Total RNA was extracted and quantified using a NanoDrop spectrophotometer and an Agilent 2100 bioanalyzer (Agilent, Santa Clara, CA, USA). A messenger RNA (mRNA) library was then constructed and amplified with Phi29 to produce 100 base pair reads on the BGIseq500 platform (BGI, Shenzhen, China). SOAPnuke (V1.5.2) was used to filter the sequencing data and Bowtie2 (V2.2.5) was used to compare the clean reads with the gene database established by Shenzhen Beijing Genomics Institute to calculate gene expression levels and identify differentially expressed genes (DEGs) (fold-change > 1.5, $q < 0.05$). The annotated DEGs were analysed using Phyper based on Gene Ontology (GO) and Kyoto Encyclopaedia of Genes and Genomes (KEGG) analysis. Gene set enrichment analysis (GSEA) was used to evaluate DEGs enriched for either negatively or positively correlated genes.

RNA Interference

Small interfering RNAs (siRNAs) and the negative control RNA duplex (siRNA-NC) were purchased from GenePharma Biotech (Shanghai, China). The miR-320a-3p mimics or miR-320a-3p inhibitor and scrambled oligonucleotides (mimics NC or inhibitor-NC) were purchased from GenePharma Biotech. These were transfected into cells during the logarithmic growth phase. The transfection was performed using the GP-transfect-Mate transfection reagent (GenePharma Biotech) according to the manufacturer's protocol. The transfected sequences of the miR-320a-3p mimics/inhibitor and siRNA oligonucleotides are shown in Additional file 1, Table S1. AgomiRs or AntagomiRs were purchased from GenePharma Biotech. CT-Exo were transfected with AntagomiR-320a-3p or AntagomiR-NC at 200 nM for 60 min at 37°C. The AgomiRs and AntagomiRs that were not transfected were removed by centrifugation at 4,000 *g* for 5 min using a 100 kDa Amicon Ultra-4 Centrifugal Filter Unit (Millipore)[26]. The internalisation of AntagomiR-NC-Cy3 by CT-Exo was assessed by qRT-PCR. Treatment with CT-Exo and other AntagomiRs was used for subsequent experiments.

Western Blotting

Total protein was extracted from cultured VSMCs, artery samples or exosomes with radioimmunoprecipitation assay (RIPA) buffer (P0013B; Beyotime). The protein concentration was measured by the BCA assay. Total protein (20–40 μg) was submitted to 8–12% sodium dodecyl sulphate-polyacrylamide gel electrophoresis (SDS-PAGE) for separation. The separated protein was transferred onto 0.2 or 0.45 μm polyvinylidene difluoride (PVDF) membranes (Millipore). The membranes were incubated in 5% non-fat milk or bovine serum albumin (BSA) (depending on the primary antibody), followed by incubation overnight with primary antibody. The

following primary antibodies were used: anti-CD9 (ab92726, Abcam, 1:2000), anti-CD81 (ab109201, Abcam, 1:1000), anti-TSG101 (bs-1365R, Bioss, 1:500), anti-RUNX2 (ab76956, Abcam, 1:1000), anti-BMP2 (bs-10696R, Bioss, 1:500), anti-p53 (10442-1-AP, Proteintech, 1:3000), anti-p62 (18420-1-AP, Proteintech, 1:2000), anti-ATG5 (66744-1-Ig, Proteintech, 1:4000), anti-LC3B (14600-1-AP, Proteintech, 1:4000, to determine the LC3B-II:LC3B-I ratio), anti-PDCD4 (12587-1-AP, Proteintech, 1:1000), anti-p-AMPK (sc33524, Santa Cruz, 1:500), anti-t-AMPK (sc25792, Santa Cruz, 1:500), anti-p-mTOR (2971, CST, 1:1000), anti-t-mTOR (2983, CST, 1:1000), anti-β-actin (20536-1-AP, Proteintech, 1:3000) and anti-GAPDH (10494-1-AP, Proteintech, 1:5000). After washing the blots, they were incubated in secondary antibody conjugated to horseradish peroxidase (SA00001-1 or SA00001-2, Proteintech, 1:5000) for 1 h at room temperature. The immunoreactive bands were visualised with chemiluminescent assay using a chemiluminescence kit (RPN2232, Amersham Biosciences Ltd., UK) and then analysed with an Amersham Imager 600 (General Electric, USA) and Image-Pro Plus software (version 6.0). The relative protein expression level was normalised to the intensity of the β-actin or GAPDH band.

Luciferase Reporter Assay

For the luciferase reporter assay, VSMCs were co-transfected with a luciferase reporter carrying the wild-type PDCD4 3′-untranslated region (UTR), a mutant PDCD4 3′-UTR and miR-320a-3p mimics or scramble oligonucleotides. Forty-eight hours after transfection, luciferase activity was quantified with the luciferase assay system (Promega, Madison, WI, USA). The nucleotide sequences of primers for the construction and mutation of 3′ UTR PDCD4 mRNA were purchased from Ribobio (Guangzhou, China).

Immunohistochemistry

As mentioned above, the expression of RUNX2 and p21 in aortic tissue was examined by immunohistochemistry[45]. In brief, arterial tissue sections were incubated at 65°C for 2 h, dewaxed in turpentine twice for 10 min each; and rehydrated in 99%, 85% and 75% ethanol for 5 min each. Antigen retrieval was performed in a trypsin-EDTA solution. Next, sections were blocked with 5% BSA for 30 min at room temperature and incubated with specific primary antibodies, including anti-RUNX2 (bs-1134R, Bioss, 1:300) and anti-21 (10355-1-AP, Proteintech, 1:400) at 4°C overnight. The following day, sections were incubated with the appropriate secondary antibody conjugated to horseradish peroxidase (PV-9000, ZSGB-BIO, Beijing, China) at room temperature for 30 min. For control experiments, the primary antibody was replaced by PBS. Finally, the sections were incubated with DAB chromogenic solution (DA1015; Solarbio) for 1 min at room temperature. Nuclei were counterstained with haematoxylin (Solarbio) for 1 min at room temperature. The stained tissue was observed under a CX31 light microscope (Olympus Corporation, Japan). Images were taken at 100× magnification and images analysed using Image-Pro Plus software (version 6.0).

Analysis of Vascular Calcium Content

Arterial samples were decalcified with 0.6 N HCl at 4°C for 48 h. After determining the protein concentration, the calcium content in the supernatant was assessed using a commercial kit (C004-2-1; Nanjing Jiancheng Bioengineering Institute). The vascular calcium content was normalised to the protein concentration.

Statistical Analysis

All data are presented as the mean ± standard deviation of three independent experiments. Data were analysed and plotted using GraphPad Prism software (San Diego, CA, USA) and ImageJ software (National Institutes of Health, Bethesda, MD, USA). The unpaired, two-tailed Student's *t*-test was conducted to compare two groups. One- or two-way analysis of variance (ANOVA) with the Bonferroni *post hoc* test was used to compare three or more groups. Results were considered significant when the *p*-value was < 0.05. In the Figures, statistical significance is indicated as ns > 0.05; *$p < 0.05$; **$p < 0.01$; ***$p < 0.001$ and ****$p < 0.0001$.

Results

CT-Exo Played a Certain Role in the Progression of Protected against VD-Induced MAC in CT Mice

Firstly, we tested the food intake and body weight of mice in the low-temperature model, and the results showed that compared with the RT group, the average food intake of mice in the CT group was significantly increased, indicating that low-temperature can increase the food intake level of mice. The measurement of the weight of mice indicated that the weight of CT group mice showed a decreasing trend within 6 days, and gradually recovered and increased after 6 days. We observed that over time, the overall body weight was attenuated despite the stable food intake in CT mice (Additional file 1: Fig. s1, a and b). The level of ALT showed no significant difference between these two groups (Additional file 1: Fig. s1c). After shaving the hair of the mice (Additional file 1: Fig. s2a), we found that the mice showed no signs of numbness or frostbite, and there was no erythema, edema, hard gangrene, infarction, or epidermal detachment on the skin surface. After dissecting the mice, the liver, lungs, spleen, heart, and kidneys were taken. The general morphology is shown in Additional file 1: Fig. s2b. The organ index can objectively reflect the function of relevant organs and is one of the important biological indicators for experimental animals. As shown in Additional file 1: Fig. s2c, except for the increase in cardiac index in the CT group, there was no significant difference in liver, spleen, lung, kidney organ indices and liver morphology (Additional file 1: Fig. s2d) between two group mice, suggesting that all CT and RT mice are in a healthy metabolic status. H&E staining of lung tissues showed that exposure to cold stress slightly aggravated lung damage. In the lung slightly disruption of the alveolar structure, as well as vascular base thickening, a mild thickened alveolar wall and minimal inflammatory cell infiltration, were observed when compared to the RT group (Additional file 1: Fig. s2d). The CT mice had higher lung interstitial inflammation score and lung wet/dry ratio compared with the RT mice, but only by trend (Additional file 1: Fig. s2, e and f).

To investigate the protective effect of CT on MAC, we subjected mice to CT or RT for 30 days and then injected with VD to induce MAC. We kept mice in the CT or RT environment throughout the experiment (Fig. 1a). Based on ARS staining of the thoracic aorta, there was a lower degree of MAC in CT mice compared with RT mice (Fig. 1, b and c). The MAC in cold-exposed mice was significantly blunted, as evidenced by the decreased calcium content (Fig. 1d). The effect of a cold environment on the body's metabolism is holistic and systemic. We wondered whether these anti-arterial calcification protective effects of CT on MAC in mice could be transferred through circulating blood factors. We collected plasma from CT mice and then intravenously injected mice with MAC with CT-Exo or CT-Exo^{free} plasma every 3 days for a total of eight times (Fig. 1e). Surprisingly, the CT-Exo group had the lowest ARS-positive area of all mice with MAC. Treatment with CT-Exo^{free} plasma slightly ameliorated the degree of MAC, as shown by the ARS staining and calcium content. However, the effect of CT-Exo^{free} plasma was much lower CT-Exo plasma, which might suggest that CT-Exo play an important role in preventing calcification formation (Fig. 1, f to h). Subsequently, we explored the role of CT-Exo in CT mice with MAC. We intraperitoneally injected the mice with the exosome inhibitor GW4869, which blocks exosome production, every other day (Fig. 1i). The ARS staining area, RUNX2 expression and arterial calcium and calcification were significantly lower in CT mice compared with CT+GW4869 mice (Fig. 1, j to n), suggesting that GW4869 reverse the protective effects of CT. These results indicate that inhibition of endogenous CT-Exo can promote MAC.

CT-Exo Mediated the CT-Induced MAC Inhibitory Effects in Mice

To directly identify the effects of exosomes, we subjected the mice to RT or CT for 30 days, isolated exosomes from them (RT-Exo or CT-Exo) and purified them by hyper-centrifugation (Additional file 1: Fig. s3a). As viewed with TEM, CT-Exo and RT-Exo exhibited a cup-like morphology ((Additional file 1: Fig. s3b). Nanoparticle tracking analysis (NTA) revealed that CT-Exo and RT-Exo had mean diameters of 110.7 ± 39.6 and 109.6 ± 40.9 nm, respectively (Additional file 1: Fig. s3c), which are similar to a previous report[47]. Western blotting showed that a vast majority of the isolated CT-Exo and RT-Exo expressed exosomal markers including TSG101, CD9 and CD81 (Additional file 1: Fig. s3d), which further indicates that these vesicles are exosomes. To determine whether exosomes could be incorporated by aortic VSMCs *in vivo*, we injected DiR-labelled CT-Exo into mice via the tail vein and tracked their distribution. We adjusted the fluorescence intensity of control mice to exclude the interference of autofluorescence. We successfully injected the DiR-labelled CT-Exo into the mice through the tail vein (Fig. 2a). Mice photography mainly detected the fluorescence signal in the liver and spleen (Additional file 1: Fig. s4). Considering that the relatively stronger fluorescence signal of the liver and spleen masked the fluorescence signals of other organs, we removed the liver and spleen, then repeated the imaging. Photographs showed that the fluorescent signals of the DiR-labeled exosomes entered the aorta after injection *in vivo* (Fig. 2a). In addition, CT-Exo injection significantly increased the expression of the exosomal marker TSG101 in VSMCs in the aortic media (Additional

file 1: Fig. s5). Moreover, TSG101 colocalised with alpha smooth muscle actin (α-SMA), which suggests that VSMCs could take up the exosomes. Hence, we successfully injected exogenous CT-Exo into mice and they were then taken up by VSMCs in the aorta.

To investigate whether CT-Exo protect VSMCs against arterial calcification *in vivo*, we analysed the calcification level by using an *in vivo* model of VD-induced MAC in mice (Fig. 2b). VD-induced mice developed significant MAC compared with the vehicle control (PBS). Intriguingly, the MAC level in CT-Exo-treated mice ranged from undetectable to just very low, as demonstrated with the ARS (Fig. 2, c and f) and Von Kossa staining (Fig. 2, d and h). At the same time, based on the staining results, RT-Exo treatment slightly weakened MAC compared with PBS treatment. Unfortunately, there was no significant inhibition of MAC in the RT-Exo group compared with the PBS group (Fig. 2, c to h). Moreover, the aortic calcium content (Fig. 2g) and RUNX2 immunostaining (Fig. 2, e and i) were significantly decreased in CT-Exo-treated mice compared with the VD-treated and RT-Exo-treated mice. These results show that CT-Exo serve as a protective factor in VD-induced MAC in mice.

CT-Exo Prevented Osteogenic Differentiation and Senescence of VSMCs via Autophagy

To determine whether CT-Exo play a vital effect on the osteogenic differentiation and senescence of VSMCs, we examined whether these exosomes could be taken up by VSMCs. We labelled CT-Exo with PKH26 and incubated VSMCs with the labelled exosomes. Fluorescence microscopy analysis showed that the labelled exosomes were taken up by the VSMCs (Fig. 3a). It is widely believed that the process of MAC is similar to bone mineralisation. ALP, RUNX2 and mineralised matrix are recognized phenotypic markers of osteoblasts and are upregulated during osteoblast differentiation of VSMCs[16, 47]. Consistent with our previous results, CT-Exo treatment significantly protected VSMCs against β-GP-induced osteogenic conversion, as demonstrated by the remarkably reduced ARS (Fig. 3, b and c) and ALP (Fig. 3f) staining of β-GP-treated VSMCs, and markedly decreased ALP activity (Fig. 3g) and the expression of RUNX2 protein (Fig. 3, h and i). VSMCs senescence were also decreased, denoted by reduced p53 expression (Fig. 3, h and i) and fewer SA-β-gal-positive VSMCs (Fig. 3, d and e). Thus, we verified that CT-Exo could protected VSMCs against osteogenic differentiation and senescence *in vitro*.

To investigate the mechanism of anti-calcification protective effect of CT-Exo, we first examined the effect of CT-Exo on autophagosome formation in VSMCs. CT-Exo increased LC3B protein expression and reduced p62 protein expression during the osteoblastic differentiation of VSMCs (Fig. 3, j and k). TEM of typical autophagic structures provided direct evidence to support the CT-Exo-mediated increase in autophagy: there were more autophagosomes in VSMCs treated with CT-Exo than in the negative and positive controls (β-GP treatment alone and PS, respectively) (Fig. 3l). Studies have shown that autophagy plays an important role in the function of VSMCs and the development of vascular diseases, suggesting that autophagy may be a potential target to prevent vascular calcification[48]. It has previously been reported that

activating the AMPK[49] and mTOR signalling pathway regulates autophagy directly and indirectly[50]. AMPK could initiate autophagy either by directly phosphorylating the serine/threonine kinase ULK1[51] or indirectly by deactivating mTORC1[52]. As shown in Additional file 1: Fig. s6a, exposure to β-GP triggered a significant elevation in ROS production in VSMCs, as revealed by the increase in the percentage of cells with green fluorescence compared with CT-Exo, suggesting that ROS-induced oxidative injury may be involved in CT-Exo-attenuated cell death. As evidenced by Annexin V-FITC/PI double staining with flow cytometry, CT-Exo treatment decreased the percentages of early apoptotic (Annexin V-FITC positive/PI negative) and late apoptotic/dead (Annexin V-FITC/PI double positive) VSMCs (Additional file 1: Fig. s6b), revealing the CT-Exo protected VSMCs from apoptosis. Taken together, these data suggest that CT-Exo prevented osteogenic differentiation and senescence of VSMCs via autophagy.

The Autophagy Inhibitor 3-MA Significantly Weakened the Pro-Autophagy Effect of CT-Exo

We next addressed the potential role of autophagy in the osteoblastic differentiation of VSMCs. RAPA, a pharmacological inducer of autophagy, can activate autophagy, cell proliferation and other cellular activities by inhibiting mTOR activity. RAPA treatment suppressed calcification and senescence of VSMCs, as demonstrated by the reduced matrix mineralisation (Fig. 4, a and b), SA-β-gal staining (Fig. 4, c and d) and ALP staining and activity (Additional file 1: Fig. s7, a and b) compared with the PS group. We used 3-MA, a pharmacological inhibitor of autophagy, to decrease autophagy during osteoblastic differentiation of VSMCs. 3-MA treatment augmented matrix mineralisation (Fig. 4a, b), SA-β-gal staining (Fig. 4, c and d) and ALP staining and activity (Additional file 1: Fig. s7, a and b) in VSMCs compared with the PS group. CT-Exo robustly protected VSMCs against osteoblastic differentiation and senescence, similar to RAPA, and the protective effect of CT-Exo could be reversed by 3-MA (Fig. 4, a to d and Additional file 1: Fig. s7, a and b).

In the mouse model of MAC (Fig. 4e), CT-Exo protected against VD-induced MAC. RAPA treatment suppressed MAC and senescence of the aortic media compared with the PS group, as demonstrated by the reduced Von Kossa staining (Fig. 4f), p21 expression (Fig. 4h), ARS staining (Additional file 1: Fig. s7, c and d) and calcium content (Additional file 1: Fig. s7e). Arterial calcification increased significantly in the group treated with 3-MA plus CT-Exo compared with the CT-Exo-treated group, demonstrated by the increased ARS (Additional file 1: Fig. s7, c and d) and Von Kossa (Fig. 4, f and g) staining, the elevated calcium content (Additional file 1: Fig. s7e) and the upregulation of p21 expression (Fig. 4, h and i) compared with the CT-Exo-treated group. Collectively, these results indicate that CT-Exo protects VSMCs against the osteoblastic differentiation and arterial calcification by promoting autophagy. 3-MA reversed the protective effect of CT-Exo on the osteoblastic differentiation of VSMCs. Thus, both *in vitro* and *in vivo*, the protective effect of CT-Exo against calcification was attenuated by blocking CT-Exo-induced autophagy.

To understand the role of the AMPK/mTOR signalling pathway in the induction of

autophagy by CT-Exo, we pre-treated VSMCs with CT-Exo for 30 min before β-GP treatment. Western blotting showed that compared with treatment with CT-Exo alone, treatment with 3-MA significantly attenuated CT-Exo-induced autophagy, reflected by the dramatic decrease in p/t-AMPK expression (Additional file 1: Fig. s7, f and g), whereas p/t-mTOR expression increased significantly (Additional file 1: Fig. s7, f and g). Interestingly, VSMCs were treated with or without CT-Exo or Compound C, an inhibitor of AMPK, or MHY1485, an activator of mTOR. In the presence of Compound C, the CT-Exo-induced inhibition of RUNX2 protein expression (Additional file 1: Fig. s8, a and b), ARS staining (Additional file 1: Fig. s8, c and e) and SA-β-gal staining (Additional file 1: Fig. s8, d and f) were abolished. Similarly, MHY1485 mimicked the effects of Compound C. Thus, these experiments demonstrate that CT-Exo inhibited osteoblastic differentiation/ageing of VSMCs via the AMPK/mTOR signalling pathway.

miR-320a-3p was Enriched in CT-Exo and Responsible for the CT-Exo-Induced protection VSMCs against Calcification/Ageing

To explore the mechanism involved in the CT-Exo-induced protection against MAC, we employed an Agilent miRNA array to compare the miRNA expression profiles of CT-Exo and RT-Exo from mouse plasma. We identified a total of 1380 miRNAs, of which 71 were differentially expressed (absolute fold-change ≥1.5, $p < 0.05$) between CT-Exo and RT-Exo. We found that 33 miRNAs were much higher and 38 miRNAs were much lower in CT-Exo compared with RT-Exo (Fig. 5a). We selected miR-320a-3p, which was the most abundant miRNA in CT-Exo compared with RT-Exo (Fig. 5b). With qRT-PCR, we assessed the changes in miR-320a-3p expression in exosomes from plasma obtained from CT and RT mice. As shown in Fig. 5b, miR-320a-3p expression was higher in CT-Exo. Moreover, miR-320a-3p expression was significantly increased in vessels from CT mice compared with vessels from RT mice (Fig. 5c). After transfection with miR-320a-3p mimics, miR-320a-3p expression in VSMCs was significantly higher than in VSMCs transfected with NC mimics, and miR-320a-3p expression in VSMCs treated with miR-320a-3p inhibitor was significantly lower than in VSMCs treated with the NC inhibitor (Fig. 5d). Moreover, miR-320a-3p overexpression with mimics greatly decreased ALP activity, while miR-320a-3p knockdown with an inhibitor greatly increased ALP activity (Fig. 5e).

Previous studies have shown that miR-320a has a certain correlation with the occurrence and development of atherosclerosis[53]. However, the role of miR-320a-3p in VSMCs calcification is largely unknown. To assess the effects of miR-320a-3p on the osteoblastic differentiation of VSMCs, we first determined the effects of miR-320a-3p overexpression or knockdown in β-GP-induced VSMCs. miR-320a-3p overexpression reduced the expression of RUNX2, BMP2 and p21 and increased the expression of LC3B and ATG5 (Fig. 5, f and g). In contrast, miR-320a-3p knockdown inhibited the level of autophagy and promoted VSMCs calcification. We then used specific AntagomiRs to silence miR-320a-3p in CT-Exo. After transfection with Antagomir-320a-3p, miR-320a-3p expression in CT-Exo decreased significantly (Fig. 5h). ARS staining showed VSMCs treated with AntagomiR-320a-3p and CT-Exo induced a much higher extent of mineralised nodule formation than VSMCs treated

with AntagomiR-NC and CT-Exo (Fig. 5, i and j). Knocking down miR-320a-3p in CT-Exo significantly reduced the ability of CT-Exo to restrain ALP activity (Fig. 5l) and ALP staining (Fig. 5k). Similarly, VSMCs treated with AntagomiR-320a-3p and CT-Exo showed accelerated senescence of VSMCs compared with VSMCs treated with AntagomiR-NC and CT-Exo (Fig. 5, m and n).

We assessed the role of miR-320a-3p in the CT-Exo-induced protection VSMCs against MAC in mice subjected to VD treatment (Fig. 6a). ARS staining indicated that ARS-positive mineralised nodule area was markedly elevated in the CT-Exo+AntagomiR-320a-3p group compared with the CT-Exo+AntagomiR-NC group (Fig. 6, b and c). The vascular calcium content analysis confirmed that CT-Exo markedly increased the vascular calcium content after pre-treatment with AntagomiR-320a-3p (Fig. 6d). These findings indicate that miR-320a-3p acts as the mediator of the CT-Exo-induced protection VSMCs against calcification. The CT-Exo+AntagomiR-320a-3p mice had slightly higher serum levels of BUN and CREA compared with the CT-Exo mice. Subsequently, both calcium and phosphorus could also be detected in CT-Exo+AntagomiR-320a-3p and CT-Exo mice, but these indicators were not significantly different between the three groups (Fig. 6, e to h). Interestingly, from the expression results of ARS staining (Fig. 6, j and k), calcium content (Fig. 6l) and RUNX2 expression (Fig. 6, m and n), we found that tail vein injection of AgomiR-320a-3p can provide a certain protective effect on arterial media calcification (Fig. 6i), but its protective effect is not as good as that of the CT-Exo group, indicating that miR-320a-3p was the main miRNA in CT-Exo, but not the only active component of CT-Exo.

miR-320a-3p Protected VSMCs Against Calcification and Ageing by Targeting Programmed Cell Death 4 (PDCD4)

To understand the mechanism by which miR-320a-3p restrained VSMCs calcification, we used the online bioinformatics tool TargetScan (Version 7.2, http://www.targetscan.org/vert_72/) and miRDB (http://mirdb.org/mirdb/index.html) and miRWalk (https://http://mirwalk.umm.uni-heidelberg.de/) to predict potential target genes of miR-320a-3p (Fig. 7a). Among them, PDCD4 is an important tumour suppressor that inhibits carcinogenesis, tumour progression and invasion by inhibiting translation[54]. Recent studies have found that PDCD4 negatively regulates autophagy by inhibiting the expression of ATG5 in tumour cells[55] and plays a certain role in autophagy in the treatment of atherosclerosis[56]. The sequence alignment results illustrated that miR-320a-3p has a complementary pairing relationship with the 3′-UTR region of PDCD4 (Fig. 7b), indicating that PDCD4 may be a target gene of miR-320a-3p. A luciferase reporter assay also demonstrated that miR-320a-3p overexpression reduced the activity of wild type PDCD4 promotor but not mutant PDCD4 promoter (Fig. 7c). In addition, western blotting showed that PDCD4 protein was downregulated by miR-320a-3p mimics and upregulated by miR-320a-3p inhibitor (Fig. 7, d and f). These data suggest that PDCD4 may be a target of miR-320a-3p in VSMCs.

To determine whether PDCD4 mediates the inhibitory effect of miR-320a-3p on VSMC calcification, we also used PDCD4-specific siRNA to block its expression.

Western blot detected that all three siPDCD4 sequences could suppress > 70% of PDCD4 protein expression; the third siRNA sequence was the most effective (Fig. 7, e and g). Hence, we used this siRNA in subsequent experiments. PDCD4 downregulation reduced the expression of RUNX2 and p53 (Fig. 7, h and i) and decreased ARS (Fig. 7, j and l) and SA-β-gal (Fig. 7, k and m) staining, indicating that PDCD4 plays a crucial role in VSMC autophagy and calcification. Notably, miR-320a-3p inhibitor enhanced the ARS and SA-β-gal stained areas, but these effects were abolished by the suppression of PDCD4 (Fig. 7, j to m). After silencing PDCD4 by siRNA and inducing calcification, we measured the expression levels of autophagy-related and phosphorylated proteins in VSMCs 3 days later. Silencing PDCD4 could promote the occurrence of autophagy in VSMCs through the AMPK/mTOR signalling pathway, which was reflected in the overexpression levels of LC3B and ATG5 proteins (Additional file 1: Fig. s9, a and b). Taken together, these results demonstrate that miR-320a-3p protected VSMCs against calcification by targeting PDCD4.

Discussion

In the present study, autophagy played a vital endogenous protective role during cold exposure under β-GP/VD induction to attenuate MAC. Furthermore, miR-320a-3p, enriched in CT-Exo, promoted autophagy and mediated the protection VSMCs against MAC. Meanwhile, PDCD4 is a target gene of miR-320a-3p that regulates autophagy to reduce MAC.

The importance of ambient temperature on mouse physiology is not limited to the context of metabolic disease. Previous studies have shown that ambient temperature has a profound effect on the physiological responses of mice to infection, tumours and ageing. For example, mice exposed to higher temperatures have better immunity to bacterial, viral and protozoal infections[57, 58]. Mice raised in thermoneutrality have much smaller tumours[59]. Hypothermia correlates with a longer lifespan[60]. Cold exposure has been reported to suppress obesity, insulin resistance, adipose dysfunction and dyslipidaemia by promoting adipocyte thermogenesis[7]. The effects of cold exposure on atherosclerosis are still under debate. Cold exposure prevents atherosclerosis by activating fat thermogenesis, suppressing vascular inflammation and improving dyslipidaemia[61, 62]. In contrast, thermoneutral conditions (30°C) increase vascular inflammation and atherosclerosis by inhibiting adipose thermogenesis[63]. Dong et al.[2] found that cold exposure promoted atherosclerotic plaque growth and instability in mice reared at 4°C with cold exposure for 3 or 7 weeks. However, another study showed that long-term cold exposure to 16°C for 8 weeks protected against Western diet–induced atherosclerosis[64]. These contradictory findings may be due to the different cold exposure conditions. Chen at al.[65] showed an inverse J-shaped association between human cardiovascular mortality and ambient temperature, suggesting that moderate cold (ranging from -1.4–22.8°C) leads to the lowest risk of cardiovascular death, but both extreme cold (-6.4 to -1.4°C) and heat (29.0–31.6°C) increase cardiovascular death risk. Seki et al.[4] exposed mice to 4°C and found that cold-activated brown fat can ‘freeze’ cancer cells to death. Based on the available research on the effect of cold stimulation on metabolism (insulin resistance, obesity,

diabetes, etc.), we found that most researchers used the temperature of 4°C[66, 67].

Prior to our study, the effect of cold exposure on MAC had not yet been studied; hence, our exploration of hypothermia and MAC is both novel and very necessary. When designing *in vivo* experimental cold exposure studies in mice, it is important to consider the different metabolic, cardiovascular and heat-sensing responses evoked by different cold stimulation temperatures. Indeed, the lack of standardisation in defining the extent of cold exposure has posed serious challenges in the field. We chose 4–8°C for 30 days to represent relatively chronic stimulation of low temperature in mice. Vascular ageing is manifested by morphological abnormalities of cells and histologically manifested as the increased deposition of collagen fibres, increased and disordered elastic fibres, arteriosclerosis and calcification[68]. We found that MAC/senescence can be weakened in mice subjected to chronic cold stimulation, mainly through CT-Exo, as demonstrated by the significantly increased calcification area of Von Kossa and ARS staining and calcium content as well as the upregulated expression of calcification and ageing marker proteins (RUNX2 and p21). *In vitro*, CT-Exo decreased SA-β-gal staining, ALP activity, RUNX2 and p53 expression and mineralised nodule formation in β-GP-induced VSMCs.

Here, using *in vitro* and *in vivo* models of arterial calcification, we found that autophagy plays a vital endogenous protective role during the osteoblastic differentiation of VSMCs. CT-Exo directly potentiated autophagy, which attenuated the osteoblastic differentiation of VSMCs *in vitro* and arterial calcification *in vivo*. Moreover, CT-Exo increased the number of autophagosomes in β-GP-induced VSMCs, increased the expression of the autophagy-related protein LC3B and decreased the expression of p62. The inhibition of autophagy by 3-MA significantly attenuated the inhibitory effect of CT-Exo on the osteoblastic differentiation of VSMCs. In contrast, the promotion of autophagy by RAPA attenuated the osteogenic differentiation of VSMCs. CT-Exo also attenuated arterial calcification by promoting autophagy in mice, as demonstrated by the fact that RAPA but not 3-MA blocked the effect of CT-Exo. Thus, targeting the autophagic pathway may help to prevent or treat vascular calcification[42, 69], which provides a theoretical basis by which CT-Exo protect against vascular calcification.

Intracellular mTOR includes two complexes, mTORC1 and mTORC2. mTORC1 regulates cellular protein synthesis and cell growth through phosphorylation and activation of downstream target proteins such as p70 ribosomal S6 kinase 1 (S6K1), while mTORC2-related signalling pathways and functions are relatively less studied. Therefore, the currently available research has mainly focused on mTORC1[70]. mTOR involves multiple pathways and there are mainly two upstream signalling pathways: the PI3K/Akt/mTOR canonical pathway and the AMPK/TSC1-TSC2/mTOR non-canonical pathway. Regulation of cell growth, proliferation, metabolism and autophagy is achieved through these two pathways[71]. mTOR signalling also plays an important role in the process of cellular senescence. Numerous studies have shown that inhibiting the mTOR signalling pathway by means of dietary restriction, RAPA or gene knockout can significantly delay cellular senescence[72, 73]. Increased mTOR activity is associated with ageing and autophagy deficits with age. The mTOR-specific inhibitor

RAPA can delay replicative senescence, reduce senescence caused by DNA damage, and reduce mitochondrial dysfunction[74]. We had previously reported that the mTOR signalling pathway is involved in the process of arterial calcification caused by trans-differentiation of VSMCs into osteoblasts and inhibiting the mTOR signalling pathway can delay vascular calcification[75]. Consistent with these findings, CT-Exo activated AMPK and inhibited mTOR in VSMCs, while AMPK inhibitors or mTOR activators abolished the CT-Exo-induced protection effects VSMCs against osteoblastic differentiation/ageing. Taken together, these results demonstrate that CT-Exo protects against arterial calcification by activating AMPK/mTOR signalling.

In recent years, researchers have found that miRNAs also play an important role in the occurrence and development of vascular ageing and ageing-related diseases[76]. Previous studies have found that miR-320a is involved in the negative regulation of osteoblastic differentiation[77] and miRNA profiling revealed that miR-320a is overexpressed in osteoporotic samples[78]. However, the role of miR-320a-3p in the senescence of VSMCs has not yet been reported. We discovered the role of plasma exosome-derived miR-320a-3p in MAC for the first time and successfully identified its relevant downstream target gene, namely PDCD4. In contrast, miR-320a-3p silencing in VSMCs almost completely reversed these anti-calcification effects. Furthermore, we confirmed that miR-320a-3p knockdown in the context of CT-Exo treatment eliminates the anti-MAC effect in mice. PDCD4 is a transcriptional and translational inhibitor and tumour suppressor. Recent studies have shown that PDCD4 may also be involved in some inflammatory diseases[79] and negatively regulate autophagy[56]. Jiang et al.[80] found that PDCD4 deficiency attenuated atherosclerosis (a chronic inflammation of the arterial wall) in hyperlipidaemic mice partly by upregulating the anti-inflammatory cytokine IL-10. Meanwhile, Wang et al.[56] showed that endogenous PDCD4 promotes the formation of macrophage foam cells and the development of atherosclerosis by inhibiting autophagy. PDCD4 downregulation by miR-21 protects cardiomyocytes from ischaemia/reperfusion or ROS-induced injury[81]. Our study shows that endogenous PDCD4 promotes medial calcification/senescence and thus represents a potential therapeutic target for patients with MAC.

If the content of this study is transformed into research, it is obvious that it is impractical to collect exosomes from individuals exposed to cold environments and transplant them to other patients. Moveover, nucleic acids themselves are acidic and highly unstable in the blood, making it difficult to penetrate cell membranes. How to deliver drugs into cells from outside the body is a challenge and how to target drugs to diseased tissues to avoid systemic toxicity is also a problem. For these reasons, we suggest overexpressing miRNA-320a-3p in human blood extracellular vesicles before transplantation to exert a protective effect against arterial media calcification. Exosomes, as a naturally domesticated endogenous nanocarrier, can maintain the biological activity of their contents *in vivo* and have the characteristics of low immunogenicity and high safety. In addition, exosomes can circulate to all compartments in the body, which has good application potential in non-liver targeted nucleic acid drug delivery. Engineering transformation can maximise the advantages of extracellular vesicles as nucleic acid drug carriers and may become the mainstream

choice for extracellular nucleic acid drug carriers in the future.

There are some limitations to this study. In addition to the changes in the composition of plasma-derived exosomes induced by cold, we hypothesised that perivascular adipose tissue and brown adipose tissue in mice also secrete factors or vesicles that play a role in the calcification of the media under cold exposure. This will be our next research direction. The chronic cold stimulation at 4–8°C leads to a state of low metabolism and the ageing and calcification of VSMCs also slows down. Next, we will continue to study the effects of extremely cold (-10–0°C) and warm (34°C) environments on MAC in mice and the effects of acute, chronic and intermittent cold exposure on MAC. We believe that these results will be helpful to guide future clinical work. Another limitation of our study is that we did not perform a 'dose-response' experiment to assess the effects of CT-Exo and RT-Exo on the vascular phenotype and the pathology of vascular calcification in normal physiology. Currently, there is no evidence for the physiological concentrations of CT-Exo and RT-Exo in vascular tissue. Future studies should use accurate assays to determine the physiological concentrations of CT-Exo and RT-Exo and to investigate whether there is a dose-dependent response in CT-Exo- and RT-Exo-treated mice. Finally, it remains to be determined whether the beneficial effects of miR-320a-3p observed in cold-exposed mice can be translated to humans. Additional work should determine the frequency, minimum intensity, duration and type of cold exposure required to prevent changes in MAC in patients and whether there are any contraindications to such interventions in certain populations[82].

Conclusion

In conclusion, we have provided the first evidence that cold exposure or CT-Exo protects against arterial calcification in VD-induced mice. Collectively, our findings suggest a novel mechanism of MAC/senescence associated with a cold environment (Fig. 8). We have also shown that CT-Exo could protect VSMCs against calcification/senescence by activating the AMPK/mTOR autophagy pathway and protecting mice against medial arterial calcification. Plasma-derived exosomes may explain the hypothermic environment-vascular calcification remission. Moreover, CT-Exo are rich in miR-320a-3p, which is the molecular basis for CT-Exo to protect agains MAC. Taken together, miR-320a-3p-enriched CT-Exo protect VSMCs against calcification/senescence by downregulating the expression of PDCD4, thereby activating the AMPK/mTOR autophagy signalling pathway. These data suggest that CT-Exo represent a novel molecular mechanism mediating blood-cardiovascular crosstalk and thus may serve as a novel potential biomarker and new target of prevention for vascular calcification and CVD.

Supplementary Information

Additional fle 1: Fig. s1 General appearance of RT and CT group mice after different temperature treatments. Body weight gain (a) and food consumption (b) of cold exposed mice and RT controls over 30 days. (c) Alanine aminotransferase (ALT). n = 6 per group; ns > 0.05; $*p < 0.05$; $**p < 0.01$; $***p < 0.001$; $****p < 0.0001$, unpaired Student's t-test. **Fig. s2** (a) Observe the appearance changes and destruction of skin

tissue in mice after shaving. (b) General morphology of organs such as the liver, lung, spleen, heart and kidney. (c) The effect of RT or CT on different organ indices in mice. (d) Representative histological sections of fixed lungs and livers were embedded in paraffin and stained with hematoxylin and eosin (H&E) (scale bars, 100 μm). (e) Evaluation of interstitial inflammation scores in lung slices of the RT and CT groups. (f) Wet/dry ratio in lung samples. n = 6 per group, ns > 0.05 and *$p < 0.05$, unpaired t test with Welch's correction. **Fig. s3** (a) Flow chart showing the extraction and isolation of plasma-derived exosomes. The purification procedure is based on differential ultracentrifugation. (b) TEM analysis of exosomes. The white scale bar is 100 nm. (c) Diameter distribution of exosomes. (d) Western blot of exosome-specific proteins TSG101, CD81 and CD9, which are abundant in CT-Exo and RT-Exo. **Fig. s4** Fluorescence signals were detected in the organs of mice after execution (n = 3 per group). **Fig. s5** Representative fluorescence micrograph showing the CT-Exo marker TSG101 (red) and smooth muscle marker α-SMA (green) in thoracic aortic sections (n = 3 per group). **Fig. s6** CT-Exo exerted effects on the ROS level and apoptosis of VSMCs. (a) DCFH-DA measures intracellular ROS production by flow cytometry. (b) Representative flow cytometric analysis of Annexin V-FITC/PI-stained VSMCs receiving different treatments for 3 days (n = 4 per group). **Fig. s7** The autophagy inhibitor 3-MA, through the AMPK/mTOR signalling pathway, effectively inhibited the ability of CT-Exo to promote osteogenic differentiation. (a) Representative images of ALP staining of VSMCs that had been pre-treated with the indicated concentrations of 3-MA or rapamycin for 30 min and then incubated with β-GP for 14 days (n = 5 per group). The scale bar is 200 μm. (b) Quantitative analysis of the ALP activity. (c, d) ARS staining showing calcified aorta from CRTL, PS, CT-Exo, 3-MA, CT-Exo+3-MA and RAPA mice (n = 5 per group). The black scale bar is 200 μm. (e) Vascular calcium content measurement. (f) The expression of p/t-AMPK and p/t-mTOR was determined with western blot in calcified VSMCs treated with CT-Exo, 3-MA or 3-MA+CT-Exo (n = 4 per group). (g) Quantitative analysis of western blotting results. The CTRL group represented the negative control group with only PBS treatment. The PS group represented the positive control group with only β-GP treatment. The data are expressed as the mean ± standard deviation. The data were analysed with one-way ANOVA with the Bonferroni *post hoc* test or the unpaired, two-tailed Student's t-test. *$p < 0.05$; **$p < 0.01$; ***$p < 0.001$; ****$p < 0.0001$. **Fig. s8** The AMPK/mTOR signalling pathway mediated defensive roles of CT-Exo on calcification/aging of VSMCs. (a) Expression of p-mTOR and p-AMPK in the β-GP-induced VSMCs treated with Compound C or MHY1485 were analysed by western blot (n = 4 per group). (b) The data are presented as densitometric ratios of RUNX2/GAPDH, p/t-mTOR and p/t-AMPK respectively. (c, d) Representative micrographs of ARS and SA-β-gal staining view were shown (n = 5 per group). (e, f) The data are presented as the ratio of positive staining area, shown as the mean ± standard deviation. The data were analysed with one-way ANOVA with the Bonferroni *post hoc* test or the unpaired, two-tailed Student's t-test. *$p < 0.05$; **$p < 0.01$; ***$p < 0.001$; ****$p < 0.0001$. **Fig. s9** siPDCD4 can activate the AMPK/mTOR signalling pathway to promote VSMCs autophagy. Western blot analysis (a) and quantification (b) of LC3B, ATG5, p53, p/t-AMPK and p/t-mTOR in VSMCs treated

with siPDCD#3 or siRNA control (n = 4 per group). The CTRL group represented the negative control group with only PBS treatment. The data are presented as the mean ± standard deviation. The data were analysed one-way ANOVA with the Bonferroni *post hoc* test. *$p < 0.05$; ****$p < 0.0001$.

Acknowledgments

We sincerely thank the animal care staff and technicians of the Animal Experimental Center of the Second Xiangya Hospital of Central South University for their care and special treatment of these mice.

Author's Contributors

Conceptualization: L-Q.Y., F-X-Z.L. and K-X. T. Supervision: F-X-Z.L., S-K.S., F.X., M-H.Z., L-M.L. and X.L. Investigation: F-X-Z.L., B.G., C-C.L., K-X.T., Y-C.C., Y-Y.W., J-Y.D., Y-L.W., S-Y.H., X.C. and F.W. Visualization: F-X-Z.L. and L-Q.Y. Resources: F-X-Z.L., J-J.L., F.X. and L-Q.Y. Writing—original draft: F-X-Z.L., J-J.L. and L-Q.Y. Reviewing, editing, and funding acquisition: F.X. and L-Q.Y. All authors read and approved the final manuscript.

Funding

This study was supported by the National Natural Science Foundation of China (No. 82071593, 81974223, 81770833 and 82100944), Key R&D Plan Hunan Province (2020SK2078) and Natural Science Foundation of Hunan Province (No. 2021JJ30036 and 2021JJ40842).

Availability of data and materials

All data generated and analyzed during this research are included in this published article.

Declarations

Ethics approval and consent to participate

All experiments were reviewed and approved by the Ethics Committee of the Second Xiangya Hospital, Central South University. All the procedures conformed to the Guide for the Care and Use of Laboratory Animals, NIH publication (8th edition, 2011). All the animal protocols were formally approved by the Ethics Committee of the Second Xiangya Hospital, Central South University (2022708).

Consent for publication

All authors agree for publication.

Competing interests

The authors declare no confict of interest.

Author details

[1]Department of Metabolism and Endocrinology, National Clinical Research Center for Metabolic Disease, Hunan Provincial Key Laboratory of Metabolic Bone Diseases, The Second Xiangya Hospital, Central South University, Changsha, China. [2]Department of Periodontal Division, Hunan Xiangya Stomatological Hospital, Central South University, Changsha, China. [3]Department of Radiology, The Second Xiangya Hospital, Central South University, Changsha, China. [4]Department of Pathology, The Second Xiangya Hospital, Central South University, Changsha, China.

References

1. Wee NKY, Nguyen AD, Enriquez RF, Zhang L, Herzog H, Baldock PA. Neuropeptide Y Regulation of Energy Partitioning and Bone Mass During Cold Exposure. Calcif Tissue Int. 2020;107(5):510-23.
2. Dong M, Yang X, Lim S, Cao Z, Honek J, Lu H, Zhang C, Seki T, Hosaka K, Wahlberg E, Yang J, Zhang L, Länne T, Sun B, Li X, Liu Y, Zhang Y, Cao Y. Cold exposure promotes atherosclerotic plaque growth and instability via UCP1-dependent lipolysis. Cell Metab. 2013;18(1):118-29.
3. Sacks D, Baxter B, Campbell BCV, Carpenter JS, Cognard C, Dippel D, Eesa M, Fischer U, Hausegger K, Hirsch JA, Shazam Hussain M, Jansen O, Jayaraman MV, Khalessi AA, Kluck BW, Lavine S, Meyers PM, Ramee S, Rüfenacht DA, Schirmer CM, Vorwerk D. Multisociety Consensus Quality Improvement Revised Consensus Statement for Endovascular Therapy of Acute Ischemic Stroke. Int J Stroke. 2018;13(6):612-32.
4. Du J, He Z, Xu M, Qu X, Cui J, Zhang S, Zhang S, Li H, Yu Z. Brown Adipose Tissue Rescues Bone Loss Induced by Cold Exposure. Front Endocrinol (Lausanne). 2021;12:778019.
5. Serrat MA. Environmental temperature impact on bone and cartilage growth. Compr Physiol. 2014;4(2):621-55.
6. Salehipour-Shirazi G, Ferguson LV, Sinclair BJ. Does cold activate the Drosophila melanogaster immune system? J Insect Physiol. 2017;96:29-34.
7. Ivanova YM, Blondin DP. Examining the benefits of cold exposure as a therapeutic strategy for obesity and type 2 diabetes. J Appl Physiol (1985). 2021;130(5):1448-59.
8. Liu C, Yavar Z, Sun Q. Cardiovascular response to thermoregulatory challenges. Am J Physiol Heart Circ Physiol. 2015;309(11):H1793-812.
9. Ungvari Z, Kaley G, de Cabo R, Sonntag WE, Csiszar A. Mechanisms of vascular aging: new perspectives. J Gerontol A Biol Sci Med Sci. 2010;65(10):1028-41.
10. Reesink KD, Spronck B. Constitutive interpretation of arterial stiffness in clinical studies: a methodological review. Am J Physiol Heart Circ Physiol. 2019;316(3):H693-h709.
11. Cao YC, Shan SK, Guo B, Li CC, Li FX, Zheng MH, Xu QS, Wang Y, Lei LM, Tang KX, Ou-Yang WL, Duan JY, Wu YY, Ullah MHE, Zhou ZA, Xu F, Lin X, Wu F, Liao XB, Yuan LQ. Histone Lysine Methylation Modification and Its Role in Vascular Calcification. Front Endocrinol (Lausanne). 2022;13:863708.
12. Weber T, Chirinos JA. Pulsatile arterial haemodynamics in heart failure. Eur Heart J. 2018;39(43):3847-54.
13. Chirinos JA, Segers P, Hughes T, Townsend R. Large-Artery Stiffness in Health and Disease: JACC State-of-the-Art Review. J Am Coll Cardiol. 2019;74(9):1237-63.
14. Lanzer P, Hannan FM, Lanzer JD, Janzen J, Raggi P, Furniss D, Schuchardt M, Thakker R,

Fok PW, Saez-Rodriguez J, Millan A, Sato Y, Ferraresi R, Virmani R, St Hilaire C. Medial Arterial Calcification: JACC State-of-the-Art Review. J Am Coll Cardiol. 2021;78(11):1145-65.

15. Boström K, Watson KE, Horn S, Wortham C, Herman IM, Demer LL. Bone morphogenetic protein expression in human atherosclerotic lesions. J Clin Invest. 1993;91(4):1800-9.

16. Xu F, Li FX, Lin X, Zhong JY, Wu F, Shan SK, Tan CM, Yuan LQ, Liao XB. Adipose tissue-derived omentin-1 attenuates arterial calcification via AMPK/Akt signaling pathway. Aging (Albany NY). 2019;11(20):8760-76.

17. Ye Y, Chen A, Li L, Liang Q, Wang S, Dong Q, Fu M, Lan Z, Li Y, Liu X, Ou JS, Lu L, Yan J. Repression of the antiporter SLC7A11/glutathione/glutathione peroxidase 4 axis drives ferroptosis of vascular smooth muscle cells to facilitate vascular calcification. Kidney Int. 2022;102(6):1259-75.

18. Lan Z, Chen A, Li L, Ye Y, Liang Q, Dong Q, Wang S, Fu M, Li Y, Liu X, Zhu Z, Ou JS, Qiu X, Lu L, Yan J. Downregulation of HDAC9 by the ketone metabolite β-hydroxybutyrate suppresses vascular calcification. J Pathol. 2022;258(3):213-26.

19. Liu X, Chen A, Liang Q, Yang X, Dong Q, Fu M, Wang S, Li Y, Ye Y, Lan Z, Chen Y, Ou JS, Yang P, Lu L, Yan J. Spermidine inhibits vascular calcification in chronic kidney disease through modulation of SIRT1 signaling pathway. Aging Cell. 2021;20(6):e13377.

20. Théry C, Witwer KW, Aikawa E, Alcaraz MJ, Anderson JD, Andriantsitohaina R, Antoniou A, Arab T, Archer F, Atkin-Smith GK, Ayre DC, Bach JM, Bachurski D, Baharvand H, Balaj L, Baldacchino S, Bauer NN, Baxter AA, Bebawy M, Beckham C, Bedina Zavec A, Benmoussa A, Berardi AC, Bergese P, Bielska E, Blenkiron C, Bobis-Wozowicz S, Boilard E, Boireau W, Bongiovanni A, Borràs FE, Bosch S, Boulanger CM, Breakefield X, Breglio AM, Brennan M, Brigstock DR, Brisson A, Broekman ML, Bromberg JF, Bryl-Górecka P, Buch S, Buck AH, Burger D, Busatto S, Buschmann D, Bussolati B, Buzás EI, Byrd JB, Camussi G, Carter DR, Caruso S, Chamley LW, Chang YT, Chen C, Chen S, Cheng L, Chin AR, Clayton A, Clerici SP, Cocks A, Cocucci E, Coffey RJ, Cordeiro-da-Silva A, Couch Y, Coumans FA, Coyle B, Crescitelli R, Criado MF, D'Souza-Schorey C, Das S, Datta Chaudhuri A, de Candia P, De Santana EF, De Wever O, Del Portillo HA, Demaret T, Deville S, Devitt A, Dhondt B, Di Vizio D, Dieterich LC, Dolo V, Dominguez Rubio AP, Dominici M, Dourado MR, Driedonks TA, Duarte FV, Duncan HM, Eichenberger RM, Ekström K, El Andaloussi S, Elie-Caille C, Erdbrügger U, Falcón-Pérez JM, Fatima F, Fish JE, Flores-Bellver M, Försönits A, Frelet-Barrand A, Fricke F, Fuhrmann G, Gabrielsson S, Gámez-Valero A, Gardiner C, Gärtner K, Gaudin R, Gho YS, Giebel B, Gilbert C, Gimona M, Giusti I, Goberdhan DC, Görgens A, Gorski SM, Greening DW, Gross JC, Gualerzi A, Gupta GN, Gustafson D, Handberg A, Haraszti RA, Harrison P, Hegyesi H, Hendrix A, Hill AF, Hochberg FH, Hoffmann KF, Holder B, Holthofer H, Hosseinkhani B, Hu G, Huang Y, Huber V, Hunt S, Ibrahim AG, Ikezu T, Inal JM, Isin M, Ivanova A, Jackson HK, Jacobsen S, Jay SM, Jayachandran M, Jenster G, Jiang L, Johnson SM, Jones JC, Jong A, Jovanovic-Talisman T, Jung S, Kalluri R, Kano SI, Kaur S, Kawamura Y, Keller ET, Khamari D, Khomyakova E, Khvorova A, Kierulf P, Kim KP, Kislinger T, Klingeborn M, Klinke DJ, 2nd, Kornek M, Kosanović MM, Kovács Á F, Krämer-Albers EM, Krasemann S, Krause M, Kurochkin IV, Kusuma GD, Kuypers S, Laitinen S, Langevin SM, Languino LR, Lannigan J, Lässer C, Laurent LC, Lavieu G, Lázaro-Ibáñez E, Le Lay S, Lee MS, Lee YXF, Lemos DS, Lenassi M, Leszczynska A, Li IT, Liao K, Libregts SF, Ligeti E, Lim

R, Lim SK, Linē A, Linnemannstöns K, Llorente A, Lombard CA, Lorenowicz MJ, Lörincz Á M, Lötvall J, Lovett J, Lowry MC, Loyer X, Lu Q, Lukomska B, Lunavat TR, Maas SL, Malhi H, Marcilla A, Mariani J, Mariscal J, Martens-Uzunova ES, Martin-Jaular L, Martinez MC, Martins VR, Mathieu M, Mathivanan S, Maugeri M, McGinnis LK, McVey MJ, Meckes DG, Jr., Meehan KL, Mertens I, Minciacchi VR, Möller A, Møller Jørgensen M, Morales-Kastresana A, Morhayim J, Mullier F, Muraca M, Musante L, Mussack V, Muth DC, Myburgh KH, Najrana T, Nawaz M, Nazarenko I, Nejsum P, Neri C, Neri T, Nieuwland R, Nimrichter L, Nolan JP, Nolte-'t Hoen EN, Noren Hooten N, O'Driscoll L, O'Grady T, O'Loghlen A, Ochiya T, Olivier M, Ortiz A, Ortiz LA, Osteikoetxea X, Østergaard O, Ostrowski M, Park J, Pegtel DM, Peinado H, Perut F, Pfaffl MW, Phinney DG, Pieters BC, Pink RC, Pisetsky DS, Pogge von Strandmann E, Polakovicova I, Poon IK, Powell BH, Prada I, Pulliam L, Quesenberry P, Radeghieri A, Raffai RL, Raimondo S, Rak J, Ramirez MI, Raposo G, Rayyan MS, Regev-Rudzki N, Ricklefs FL, Robbins PD, Roberts DD, Rodrigues SC, Rohde E, Rome S, Rouschop KM, Rughetti A, Russell AE, Saá P, Sahoo S, Salas-Huenuleo E, Sánchez C, Saugstad JA, Saul MJ, Schiffelers RM, Schneider R, Schøyen TH, Scott A, Shahaj E, Sharma S, Shatnyeva O, Shekari F, Shelke GV, Shetty AK, Shiba K, Siljander PR, Silva AM, Skowronek A, Snyder OL, 2nd, Soares RP, Sódar BW, Soekmadji C, Sotillo J, Stahl PD, Stoorvogel W, Stott SL, Strasser EF, Swift S, Tahara H, Tewari M, Timms K, Tiwari S, Tixeira R, Tkach M, Toh WS, Tomasini R, Torrecilhas AC, Tosar JP, Toxavidis V, Urbanelli L, Vader P, van Balkom BW, van der Grein SG, Van Deun J, van Herwijnen MJ, Van Keuren-Jensen K, van Niel G, van Royen ME, van Wijnen AJ, Vasconcelos MH, Vechetti IJ, Jr., Veit TD, Vella LJ, Velot É, Verweij FJ, Vestad B, Viñas JL, Visnovitz T, Vukman KV, Wahlgren J, Watson DC, Wauben MH, Weaver A, Webber JP, Weber V, Wehman AM, Weiss DJ, Welsh JA, Wendt S, Wheelock AM, Wiener Z, Witte L, Wolfram J, Xagorari A, Xander P, Xu J, Yan X, Yáñez-Mó M, Yin H, Yuana Y, Zappulli V, Zarubova J, Žėkas V, Zhang JY, Zhao Z, Zheng L, Zheutlin AR, Zickler AM, Zimmermann P, Zivkovic AM, Zocco D, Zuba-Surma EK. Minimal information for studies of extracellular vesicles 2018 (MISEV2018): a position statement of the International Society for Extracellular Vesicles and update of the MISEV2014 guidelines. J Extracell Vesicles. 2018;7(1):1535750.

21. Luo ZW, Li FX, Liu YW, Rao SS, Yin H, Huang J, Chen CY, Hu Y, Zhang Y, Tan YJ, Yuan LQ, Chen TH, Liu HM, Cao J, Liu ZZ, Wang ZX, Xie H. Aptamer-functionalized exosomes from bone marrow stromal cells target bone to promote bone regeneration. Nanoscale. 2019;11(43):20884-92.

22. Liu J, Li F, Liu B, Yao Z, Li L, Liu G, Peng L, Wang Y, Huang J. Adipose-derived mesenchymal stem cell exosomes inhibit transforming growth factor-β1-induced collagen synthesis in oral mucosal fibroblasts. Exp Ther Med. 2021;22(6):1419.

23. Li FX, Lin X, Xu F, Shan SK, Guo B, Lei LM, Zheng MH, Wang Y, Xu QS, Yuan LQ. The Role of Mesenchymal Stromal Cells-Derived Small Extracellular Vesicles in Diabetes and Its Chronic Complications. Front Endocrinol (Lausanne). 2021;12:780974.

24. Li FX, Liu JJ, Xu F, Lin X, Zhong JY, Wu F, Yuan LQ. Role of tumor-derived exosomes in bone metastasis. Oncol Lett. 2019;18(4):3935-45.

25. Wu YL, Lin ZJ, Li CC, Lin X, Shan SK, Guo B, Zheng MH, Li F, Yuan LQ, Li ZH. Epigenetic regulation in metabolic diseases: mechanisms and advances in clinical study. Signal Transduct Target Ther. 2023;8(1):98.

26. Wang ZX, Luo ZW, Li FX, Cao J, Rao SS, Liu YW, Wang YY, Zhu GQ, Gong JS, Zou JT, Wang Q, Tan YJ, Zhang Y, Hu Y, Li YY, Yin H, Wang XK, He ZH, Ren L, Liu ZZ, Hu XK, Yuan LQ, Xu R, Chen CY, Xie H. Aged bone matrix-derived extracellular vesicles as a messenger for calcification paradox. Nat Commun. 2022;13(1):1453.

27. Lin X, Shan SK, Xu F, Zhong JY, Wu F, Duan JY, Guo B, Li FX, Wang Y, Zheng MH, Xu QS, Lei LM, Ou-Yang WL, Wu YY, Tang KX, Ullah MHE, Liao XB, Yuan LQ. The crosstalk between endothelial cells and vascular smooth muscle cells aggravates high phosphorus-induced arterial calcification. Cell Death Dis. 2022;13(7):650.

28. Guo B, Shan SK, Xu F, Lin X, Li FX, Wang Y, Xu QS, Zheng MH, Lei LM, Li CC, Zhou ZA, Ullah MHE, Wu F, Liao XB, Yuan LQ. Protective role of small extracellular vesicles derived from HUVECs treated with AGEs in diabetic vascular calcification. J Nanobiotechnology. 2022;20(1):334.

29. Wu YY, Shan SK, Lin X, Xu F, Zhong JY, Wu F, Duan JY, Guo B, Li FX, Wang Y, Zheng MH, Xu QS, Lei LM, Ou-Yang WL, Tang KX, Li CC, Ullah MHE, Yuan LQ. Cellular Crosstalk in the Vascular Wall Microenvironment: The Role of Exosomes in Vascular Calcification. Front Cardiovasc Med. 2022;9:912358.

30. Fleming A, Bourdenx M, Fujimaki M, Karabiyik C, Krause GJ, Lopez A, Martín-Segura A, Puri C, Scrivo A, Skidmore J, Son SM, Stamatakou E, Wrobel L, Zhu Y, Cuervo AM, Rubinsztein DC. The different autophagy degradation pathways and neurodegeneration. Neuron. 2022;110(6):935-66.

31. Menzies FM, Fleming A, Caricasole A, Bento CF, Andrews SP, Ashkenazi A, Füllgrabe J, Jackson A, Jimenez Sanchez M, Karabiyik C, Licitra F, Lopez Ramirez A, Pavel M, Puri C, Renna M, Ricketts T, Schlotawa L, Vicinanza M, Won H, Zhu Y, Skidmore J, Rubinsztein DC. Autophagy and Neurodegeneration: Pathogenic Mechanisms and Therapeutic Opportunities. Neuron. 2017;93(5):1015-34.

32. White E, Lattime EC, Guo JY. Autophagy Regulates Stress Responses, Metabolism, and Anticancer Immunity. Trends Cancer. 2021;7(8):778-89.

33. Deretic V. Autophagy in inflammation, infection, and immunometabolism. Immunity. 2021;54(3):437-53.

34. Gao W, Wang X, Zhou Y, Wang X, Yu Y. Autophagy, ferroptosis, pyroptosis, and necroptosis in tumor immunotherapy. Signal Transduct Target Ther. 2022;7(1):196.

35. Kaushik S, Tasset I, Arias E, Pampliega O, Wong E, Martinez-Vicente M, Cuervo AM. Autophagy and the hallmarks of aging. Ageing Res Rev. 2021;72:101468.

36. Wilhelm T, Richly H. Autophagy during ageing - from Dr Jekyll to Mr Hyde. Febs j. 2018;285(13):2367-76.

37. Qiao L, Ma J, Zhang Z, Sui W, Zhai C, Xu D, Wang Z, Lu H, Zhang M, Zhang C, Chen W, Zhang Y. Deficient Chaperone-Mediated Autophagy Promotes Inflammation and Atherosclerosis. Circ Res. 2021;129(12):1141-57.

38. Tao H, Yancey PG, Blakemore JL, Zhang Y, Ding L, Jerome WG, Brown JD, Vickers KC, Linton MF. Macrophage SR-BI modulates autophagy via VPS34 complex and PPARα transcription of Tfeb in atherosclerosis. J Clin Invest. 2021;131(7).

39. Chen Z, Ouyang C, Zhang H, Gu Y, Deng Y, Du C, Cui C, Li S, Wang W, Kong W, Chen J, Cai J, Geng B. Vascular smooth muscle cell-derived hydrogen sulfide promotes atherosclerotic plaque stability via TFEB (transcription factor EB)-mediated autophagy.

Autophagy. 2022:1-18.

40. Forte M, Bianchi F, Cotugno M, Marchitti S, De Falco E, Raffa S, Stanzione R, Di Nonno F, Chimenti I, Palmerio S, Pagano F, Petrozza V, Micaloni A, Madonna M, Relucenti M, Torrisi MR, Frati G, Volpe M, Rubattu S, Sciarretta S. Pharmacological restoration of autophagy reduces hypertension-related stroke occurrence. Autophagy. 2020;16(8):1468-81.

41. Li FF, Shang XK, Du XL, Chen S. Rapamycin Treatment Attenuates Angiotensin II -induced Abdominal Aortic Aneurysm Formation via VSMC Phenotypic Modulation and Down-regulation of ERK1/2 Activity. Curr Med Sci. 2018;38(1):93-100.

42. Peng YQ, Xiong D, Lin X, Cui RR, Xu F, Zhong JY, Zhu T, Wu F, Mao MZ, Liao XB, Yuan LQ. Oestrogen Inhibits Arterial Calcification by Promoting Autophagy. Sci Rep. 2017;7(1):3549.

43. Horowitz AM, Fan X, Bieri G, Smith LK, Sanchez-Diaz CI, Schroer AB, Gontier G, Casaletto KB, Kramer JH, Williams KE, Villeda SA. Blood factors transfer beneficial effects of exercise on neurogenesis and cognition to the aged brain. Science. 2020;369(6500):167-73.

44. Dinkins MB, Dasgupta S, Wang G, Zhu G, Bieberich E. Exosome reduction in vivo is associated with lower amyloid plaque load in the 5XFAD mouse model of Alzheimer's disease. Neurobiol Aging. 2014;35(8):1792-800.

45. Xu F, Zhong JY, Lin X, Shan SK, Guo B, Zheng MH, Wang Y, Li F, Cui RR, Wu F, Zhou E, Liao XB, Liu YS, Yuan LQ. Melatonin alleviates vascular calcification and ageing through exosomal miR-204/miR-211 cluster in a paracrine manner. J Pineal Res. 2020;68(3):e12631.

46. Wu F, Lin X, Shan SK, Li F, Xu F, Zhong JY, Guo B, Zheng MH, Wang Y, Mo ZH, Yuan LQ. The Suppression of miR-199a-3p by Promoter Methylation Contributes to Papillary Thyroid Carcinoma Aggressiveness by Targeting RAP2a and DNMT3a. Front Cell Dev Biol. 2020;8:594528.

47. Viegas CSB, Santos L, Macedo AL, Matos AA, Silva AP, Neves PL, Staes A, Gevaert K, Morais R, Vermeer C, Schurgers L, Simes DC. Chronic Kidney Disease Circulating Calciprotein Particles and Extracellular Vesicles Promote Vascular Calcification: A Role for GRP (Gla-Rich Protein). Arterioscler Thromb Vasc Biol. 2018;38(3):575-87.

48. Tai S, Hu XQ, Peng DQ, Zhou SH, Zheng XL. The roles of autophagy in vascular smooth muscle cells. Int J Cardiol. 2016;211:1-6.

49. Piwkowska A, Rogacka D, Jankowski M, Dominiczak MH, Stepiński JK, Angielski S. Metformin induces suppression of NAD(P)H oxidase activity in podocytes. Biochem Biophys Res Commun. 2010;393(2):268-73.

50. Kim J, Kundu M, Viollet B, Guan KL. AMPK and mTOR regulate autophagy through direct phosphorylation of Ulk1. Nat Cell Biol. 2011;13(2):132-41.

51. Egan D, Kim J, Shaw RJ, Guan KL. The autophagy initiating kinase ULK1 is regulated via opposing phosphorylation by AMPK and mTOR. Autophagy. 2011;7(6):643-4.

52. Wang S, Song P, Zou MH. AMP-activated protein kinase, stress responses and cardiovascular diseases. Clin Sci (Lond). 2012;122(12):555-73.

53. Zhang C, Wang X. miR-320a Targeting RGS5 Aggravates Atherosclerosis by Promoting Migration and Proliferation of ox-LDL-Stimulated Vascular Smooth Muscle Cells. J Cardiovasc Pharmacol. 2022;80(1):110-17.

54. Lankat-Buttgereit B, Göke R. The tumour suppressor Pdcd4: recent advances in the elucidation of function and regulation. Biol Cell. 2009;101(6):309-17.

55. Song X, Zhang X, Wang X, Zhu F, Guo C, Wang Q, Shi Y, Wang J, Chen Y, Zhang L. Tumor suppressor gene PDCD4 negatively regulates autophagy by inhibiting the expression of autophagy-related gene ATG5. Autophagy. 2013;9(5):743-55.
56. Wang L, Jiang Y, Song X, Guo C, Zhu F, Wang X, Wang Q, Shi Y, Wang J, Gao F, Zhao W, Chen YH, Zhang L. Pdcd4 deficiency enhances macrophage lipoautophagy and attenuates foam cell formation and atherosclerosis in mice. Cell Death Dis. 2016;7(1):e2055.
57. Bell JF, Moore GJ. Effects of high ambient temperature on various stages of rabies virus infection in mice. Infect Immun. 1974;10(3):510-5.
58. Amrein YU. Effects of environmental temperature on Trypanosoma cruzi infection in mice. J Parasitol. 1967;53(6):1160.
59. Kokolus KM, Capitano ML, Lee CT, Eng JW, Waight JD, Hylander BL, Sexton S, Hong CC, Gordon CJ, Abrams SI, Repasky EA. Baseline tumor growth and immune control in laboratory mice are significantly influenced by subthermoneutral housing temperature. Proc Natl Acad Sci U S A. 2013;110(50):20176-81.
60. Simonsick EM, Meier HCS, Shaffer NC, Studenski SA, Ferrucci L. Basal body temperature as a biomarker of healthy aging. Age (Dordr). 2016;38(5-6):445-54.
61. Chang L, Villacorta L, Li R, Hamblin M, Xu W, Dou C, Zhang J, Wu J, Zeng R, Chen YE. Loss of perivascular adipose tissue on peroxisome proliferator-activated receptor-γ deletion in smooth muscle cells impairs intravascular thermoregulation and enhances atherosclerosis. Circulation. 2012;126(9):1067-78.
62. Reynés B, van Schothorst EM, García-Ruiz E, Keijer J, Palou A, Oliver P. Cold exposure down-regulates immune response pathways in ferret aortic perivascular adipose tissue. Thromb Haemost. 2017;117(5):981-91.
63. Tian XY, Ganeshan K, Hong C, Nguyen KD, Qiu Y, Kim J, Tangirala RK, Tontonoz P, Chawla A. Thermoneutral Housing Accelerates Metabolic Inflammation to Potentiate Atherosclerosis but Not Insulin Resistance. Cell Metab. 2016;23(1):165-78.
64. Zhang X, Zhang Y, Wang P, Zhang SY, Dong Y, Zeng G, Yan Y, Sun L, Wu Q, Liu H, Liu B, Kong W, Wang X, Jiang C. Adipocyte Hypoxia-Inducible Factor 2α Suppresses Atherosclerosis by Promoting Adipose Ceramide Catabolism. Cell Metab. 2019;30(5):937-51.e5.
65. Chen R, Yin P, Wang L, Liu C, Niu Y, Wang W, Jiang Y, Liu Y, Liu J, Qi J, You J, Kan H, Zhou M. Association between ambient temperature and mortality risk and burden: time series study in 272 main Chinese cities. Bmj. 2018;363:k4306.
66. Chevalier C, Stojanović O, Colin DJ, Suarez-Zamorano N, Tarallo V, Veyrat-Durebex C, Rigo D, Fabbiano S, Stevanović A, Hagemann S, Montet X, Seimbille Y, Zamboni N, Hapfelmeier S, Trajkovski M. Gut Microbiota Orchestrates Energy Homeostasis during Cold. Cell. 2015;163(6):1360-74.
67. Bukowiecki LJ. Energy balance and diabetes. The effects of cold exposure, exercise training, and diet composition on glucose tolerance and glucose metabolism in rat peripheral tissues. Can J Physiol Pharmacol. 1989;67(4):382-93.
68. Shanahan CM. Mechanisms of vascular calcification in CKD-evidence for premature ageing? Nat Rev Nephrol. 2013;9(11):661-70.
69. Dai XY, Zhao MM, Cai Y, Guan QC, Zhao Y, Guan Y, Kong W, Zhu WG, Xu MJ, Wang X. Phosphate-induced autophagy counteracts vascular calcification by reducing matrix

vesicle release. Kidney Int. 2013;83(6):1042-51.

70. Lin X, Xu F, Cui RR, Xiong D, Zhong JY, Zhu T, Li F, Wu F, Xie XB, Mao MZ, Liao XB, Yuan LQ. Arterial Calcification Is Regulated Via an miR-204/DNMT3a Regulatory Circuit Both In Vitro and in Female Mice. Endocrinology. 2018;159(8):2905-16.

71. Cui RR, Li SJ, Liu LJ, Yi L, Liang QH, Zhu X, Liu GY, Liu Y, Wu SS, Liao XB, Yuan LQ, Mao DA, Liao EY. MicroRNA-204 regulates vascular smooth muscle cell calcification in vitro and in vivo. Cardiovasc Res. 2012;96(2):320-9.

72. Hao J, Zhang L, Cong G, Ren L, Hao L. MicroRNA-34b/c inhibits aldosterone-induced vascular smooth muscle cell calcification via a SATB2/Runx2 pathway. Cell Tissue Res. 2016;366(3):733-46.

73. Pantsulaia I, Ciszewski WM, Niewiarowska J. Senescent endothelial cells: Potential modulators of immunosenescence and ageing. Ageing Res Rev. 2016;29:13-25.

74. Yang L, Cheng P, Chen C, He HB, Xie GQ, Zhou HD, Xie H, Wu XP, Luo XH. miR-93/Sp7 function loop mediates osteoblast mineralization. J Bone Miner Res. 2012;27(7):1598-606.

75. Maegdefessel L, Rayner KJ, Leeper NJ. MicroRNA regulation of vascular smooth muscle function and phenotype: early career committee contribution. Arterioscler Thromb Vasc Biol. 2015;35(1):2-6.

76. Lee S, Choi E, Cha MJ, Park AJ, Yoon C, Hwang KC. Impact of miRNAs on cardiovascular aging. J Geriatr Cardiol. 2015;12(5):569-74.

77. Wang CG, Hu YH, Su SL, Zhong D. LncRNA DANCR and miR-320a suppressed osteogenic differentiation in osteoporosis by directly inhibiting the Wnt/β-catenin signaling pathway. Exp Mol Med. 2020;52(8):1310-25.

78. De-Ugarte L, Yoskovitz G, Balcells S, Güerri-Fernández R, Martinez-Diaz S, Mellibovsky L, Urreizti R, Nogués X, Grinberg D, García-Giralt N, Díez-Pérez A. MiRNA profiling of whole trabecular bone: identification of osteoporosis-related changes in MiRNAs in human hip bones. BMC Med Genomics. 2015;8:75.

79. Sheedy FJ, Palsson-McDermott E, Hennessy EJ, Martin C, O'Leary JJ, Ruan Q, Johnson DS, Chen Y, O'Neill LA. Negative regulation of TLR4 via targeting of the proinflammatory tumor suppressor PDCD4 by the microRNA miR-21. Nat Immunol. 2010;11(2):141-7.

80. Jiang Y, Gao Q, Wang L, Guo C, Zhu F, Wang B, Wang Q, Gao F, Chen Y, Zhang L. Deficiency of programmed cell death 4 results in increased IL-10 expression by macrophages and thereby attenuates atherosclerosis in hyperlipidemic mice. Cell Mol Immunol. 2016;13(4):524-34.

81. Cheng Y, Zhu P, Yang J, Liu X, Dong S, Wang X, Chun B, Zhuang J, Zhang C. Ischaemic preconditioning-regulated miR-21 protects heart against ischaemia/reperfusion injury via anti-apoptosis through its target PDCD4. Cardiovasc Res. 2010;87(3):431-9.

82. Blondin DP, Haman F. Shivering and nonshivering thermogenesis in skeletal muscles. Handb Clin Neurol. 2018;156:153-73.

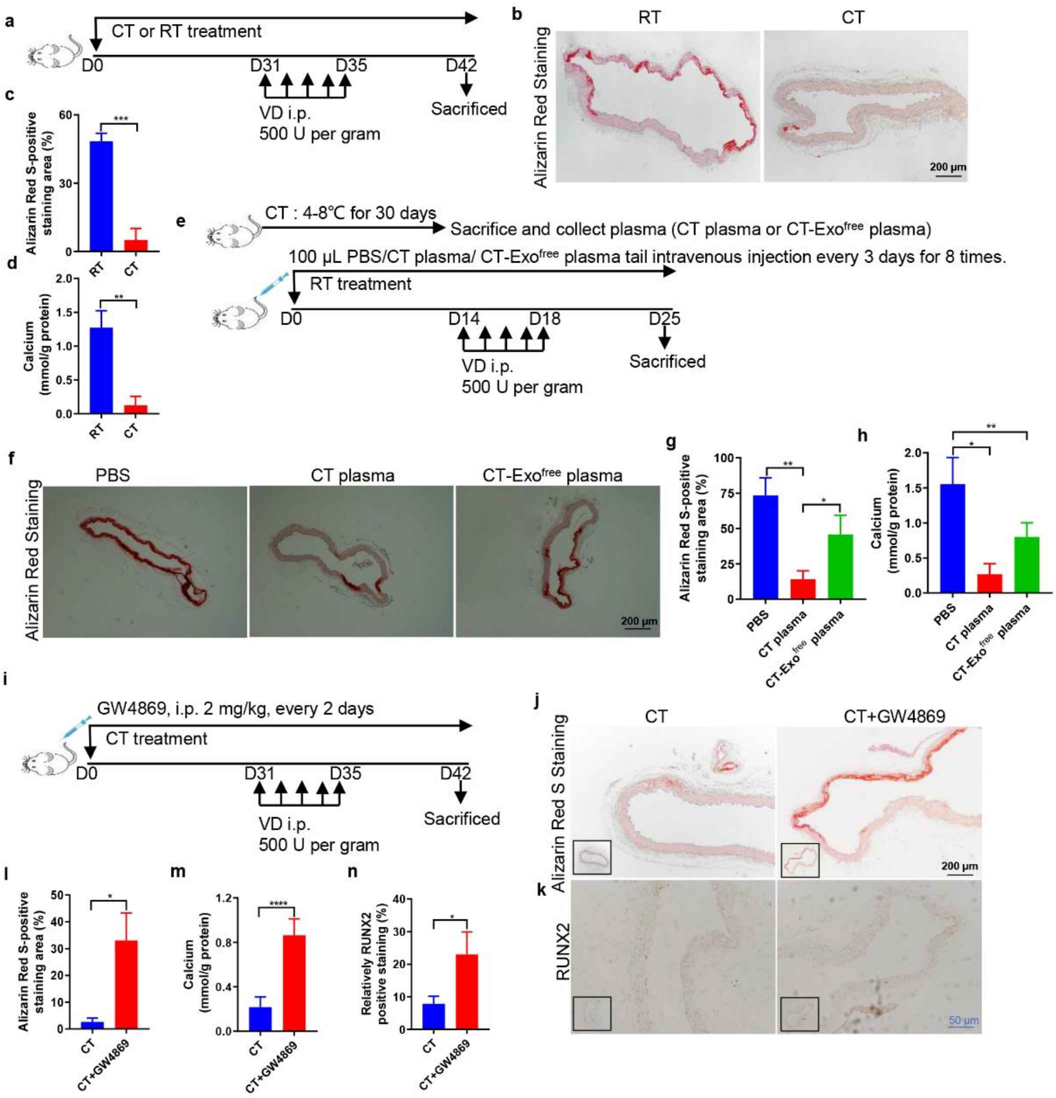

Fig. 1 Cold exposure protected against MAC in a VD-induced mouse model. (a) The schematic flow diagram represents the *in vivo* treatment of CT or RT in the VD-treated mouse model (n = 6 per group). ARS-stained sections from thoracic aorta (b) and quantitation of positive staining area (c) are shown. The black scale bar is 200 μm. (d) Vascular calcium content measurement. (e) Experimental design of the VD-induced vascular calcification mouse model treated with PBS, CT plasma or CT-Exo^{free} plasma by intravenous injection (n = 6 per group). ARS-stained sections from thoracic aorta (f) and quantitation of the positive staining area (g) are shown. The black scale bar is 200 μm. (h) Calcium content of the thoracic aorta. (i) Schematic flow diagram represented the *in vivo* treatment of CT with or without GW4869 in the VD-induced mice model (n = 6 per group). Evaluation of the effect of pre-treatment of the exosome blocker GW4869 on arterial calcification induced by VD calcified mice in CT treatment. ARS staining (j, l) and RUNX2 expression (k, n) analysis of paraffin-embedded vascular tissue from mice. (m) Vascular calcium content measurement. The black scale bar is

200 μm and the blue scale bar is 50 μm. The data are presented as the mean ± standard deviation with three replicates for each group. The data were analysed with Student's t-test or one-way ANOVA with the Bonferroni *post hoc* test. *$p < 0.05$; **$p < 0.01$; ***$p < 0.001$; ****$p < 0.0001$.

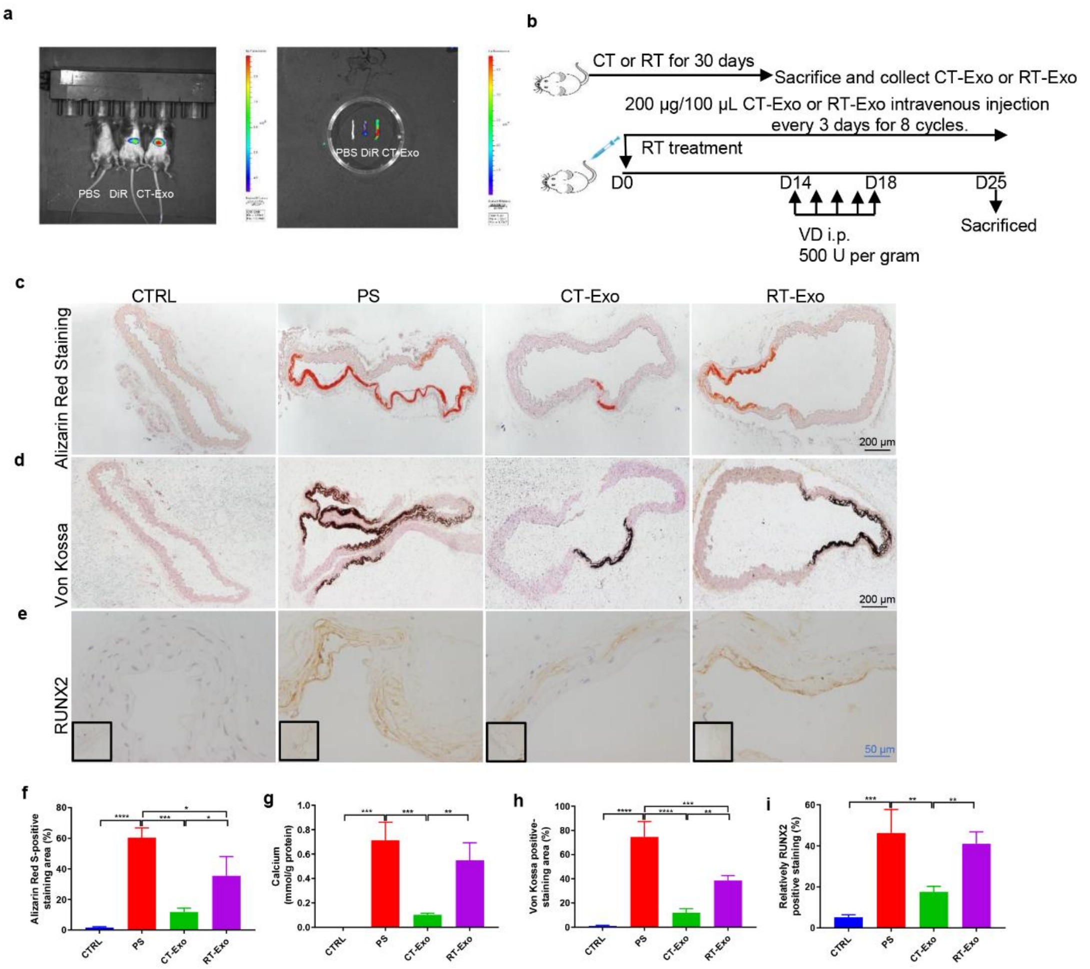

Fig. 2 CT-Exo protected against vascular calcification in the VD-induced mouse model. (a) Uptake of DiR-labelled CT-Exo in aortic VSMCs of mice. The mice were subjected to the intravenous administration of PBS, DiR or DiR-labelled CT-Exo treatments (100 μg/mice, n = 3 per group). Representative *in vivo* fluorescence image of CT-Exo distribution in mice 24 h after CT-Exo injection. (b) Experimental design of the VD-induced vascular calcification mouse model treated with PBS, CT-Exo or RT-Exo by intravenous injection (n = 6 per group). ARS (c) and Von Kossa staining (d) and quantification of the percentages of ARS+ (f) and Von Kossa+ (h) areas. (g) Vascular calcium content measurement. RUNX2 expression in thoracic aorta (e) and quantitation of positive staining area (i) are shown. The black scale bar is 200 μm and the blue scale

bar is 50 µm. The CTRL group represents the negative control group with only PBS treatment. The PS group represents the positive control group with only β-GP treatment. The data are presented as the mean ± standard deviation with three replicates for each group. The data were analysed with one-way ANOVA with the Bonferroni *post hoc* test or the unpaired, two-tailed Student's t-test. *$p < 0.05$; **$p < 0.01$; ***$p < 0.001$; ****$p < 0.0001$.

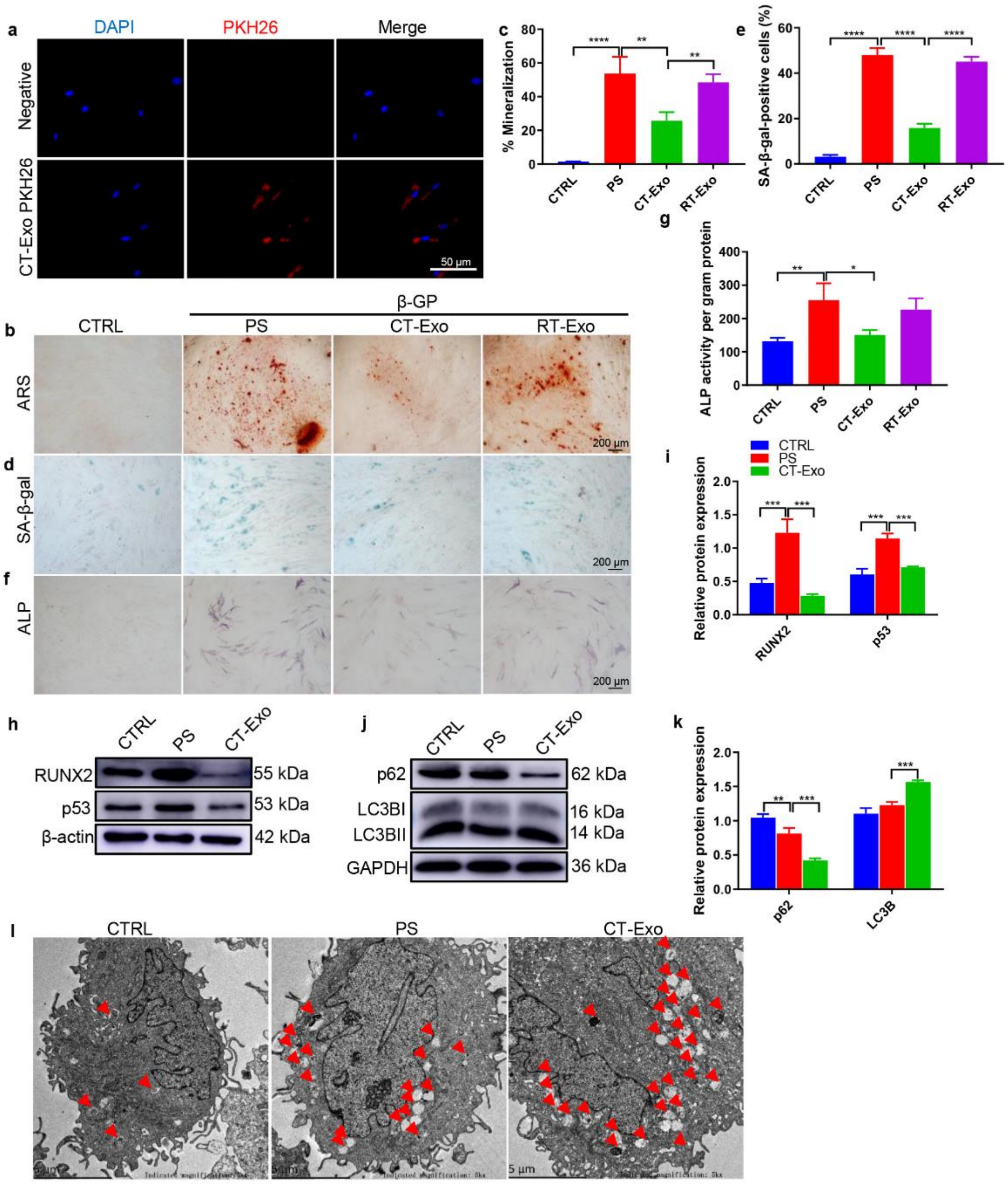

Fig. 3 CT-Exo protected VSMCs against calcification by promoting autophagy. (a) Representative fluorescence micrograph of PKH26-labelled CT-Exo (red) internalised by VSMCs; nuclei are shown in blue. The white scale bar is 50 µm. ARS (b) and SA-β-gal (d) staining was evaluated in VSMCs incubated with β-GP and CT-Exo for 28 and 10 days, respectively. n = 5, the black scale bar is 200 µm. (c, e) The data are presented as ratio of positive staining area. (f) ALP staining was measured in VSMCs incubated with β-GP and CT-Exo for 14 days. The black scale bar is 200 µm. (g) ALP activity. (h) RUNX2 and p53 protein expression was determined by western blotting after β-GP and

CT-Exo treatment for 3 days. The data are presented as densitometric ratios normalised to β-actin (i), n = 4. (j, k) Western blots (j) and quantification (k) of p62 and LC3B in the PBS, PS and CT-Exo VSMCs, n = 4. (l) VSMCs were incubated with β-GP and CT-Exo for 72 h and then analysed by electron microscopy; a representative image is shown. Autophagosomes containing organelle remnants are highlighted by red arrows (n = 4 per group). The PS group represents the control group with only β-GP treatment. Each experiment was repeated three times. The data are presented as the mean ± standard deviation with three replicates. The data were analysed with one-way ANOVA with the Bonferroni *post hoc* test. *$p < 0.05$; **$p < 0.01$; ***$p < 0.001$; ****$p < 0.0001$

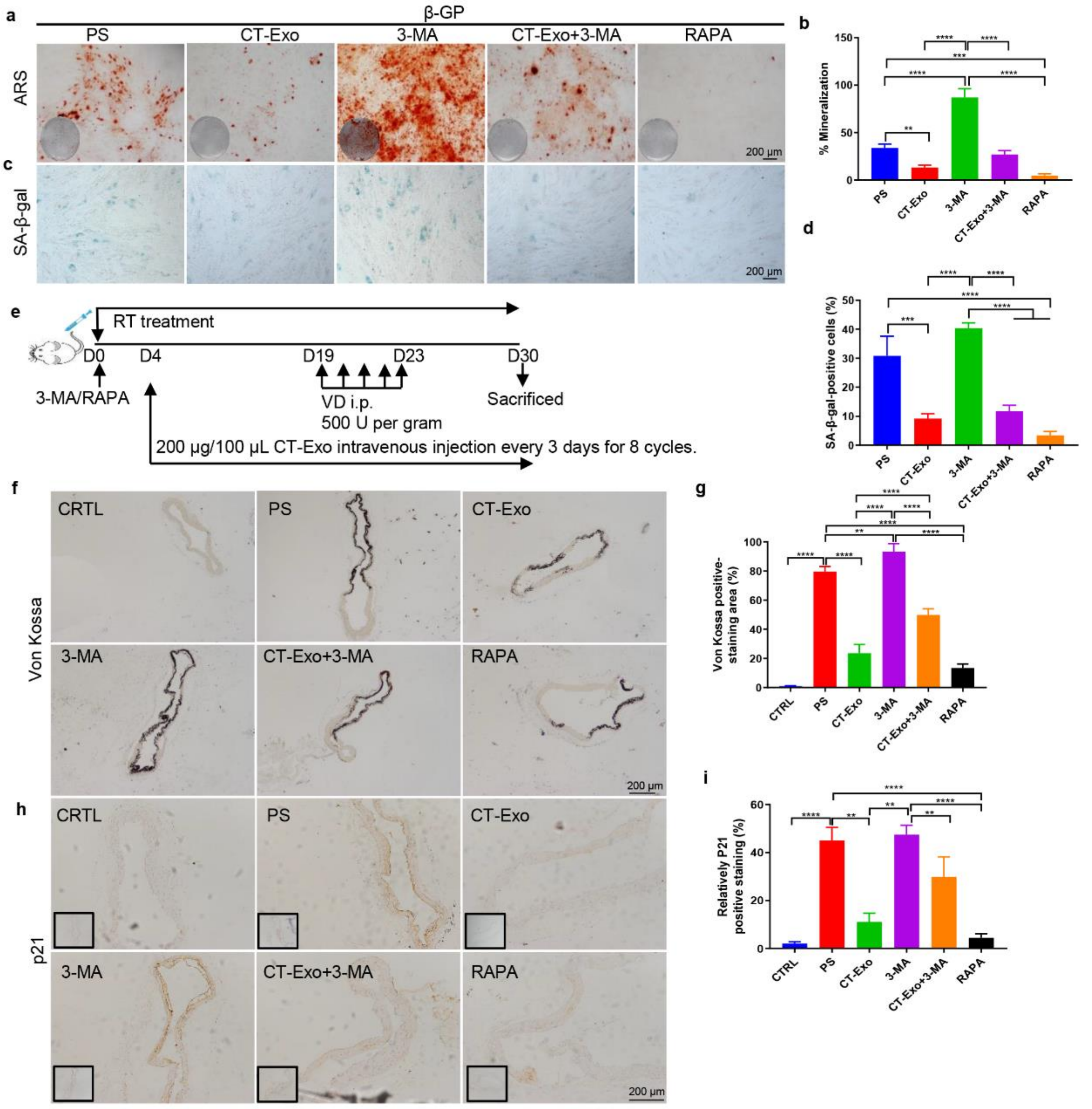

Fig. 4 3-MA attenuated the pro-aging/pro-calcification preventive effect of CT-Exo *in vitro* and *in vivo*. Representative images of ARS (a) and SA-β-gal (c) staining of VSMCs that had been pre-treated with the indicated concentrations of 3-MA or RAPA for 30 min and then incubated with β-GP for 28 and 10 days, respectively. n = 5, the scale bar is 200 μm. Quantitative analysis of the percentages of ARS+ (b, in red) and

SA-β-gal+ (d, in green) areas. (e) Schematic illustration of the experimental design used to assess the effects of CT-Exo and 3-MA on the vascular phenotype in VD-induced mice (n = 6 per group). (f, g) Von Kossa staining showed calcified aorta from CRTL, PS, CT-Exo, 3-MA, CT-Exo+3-MA and RAPA mice (n = 6 per group). The black scale bar is 200 μm. (h, i) p21 expression in aorta from the six groups of mice were examined by immunohistochemistry. The black scale bar is 200 μm (n = 6 per group). The CTRL group represents the negative control group with only PBS treatment. The PS group represents the positive control group with only β-GP treatment. The data are presented as the mean ± standard deviation. The data were analysed with one-way ANOVA with the Bonferroni *post hoc* test. *$p < 0.05$; **$p < 0.01$; ***$p < 0.001$; ****$p < 0.0001$

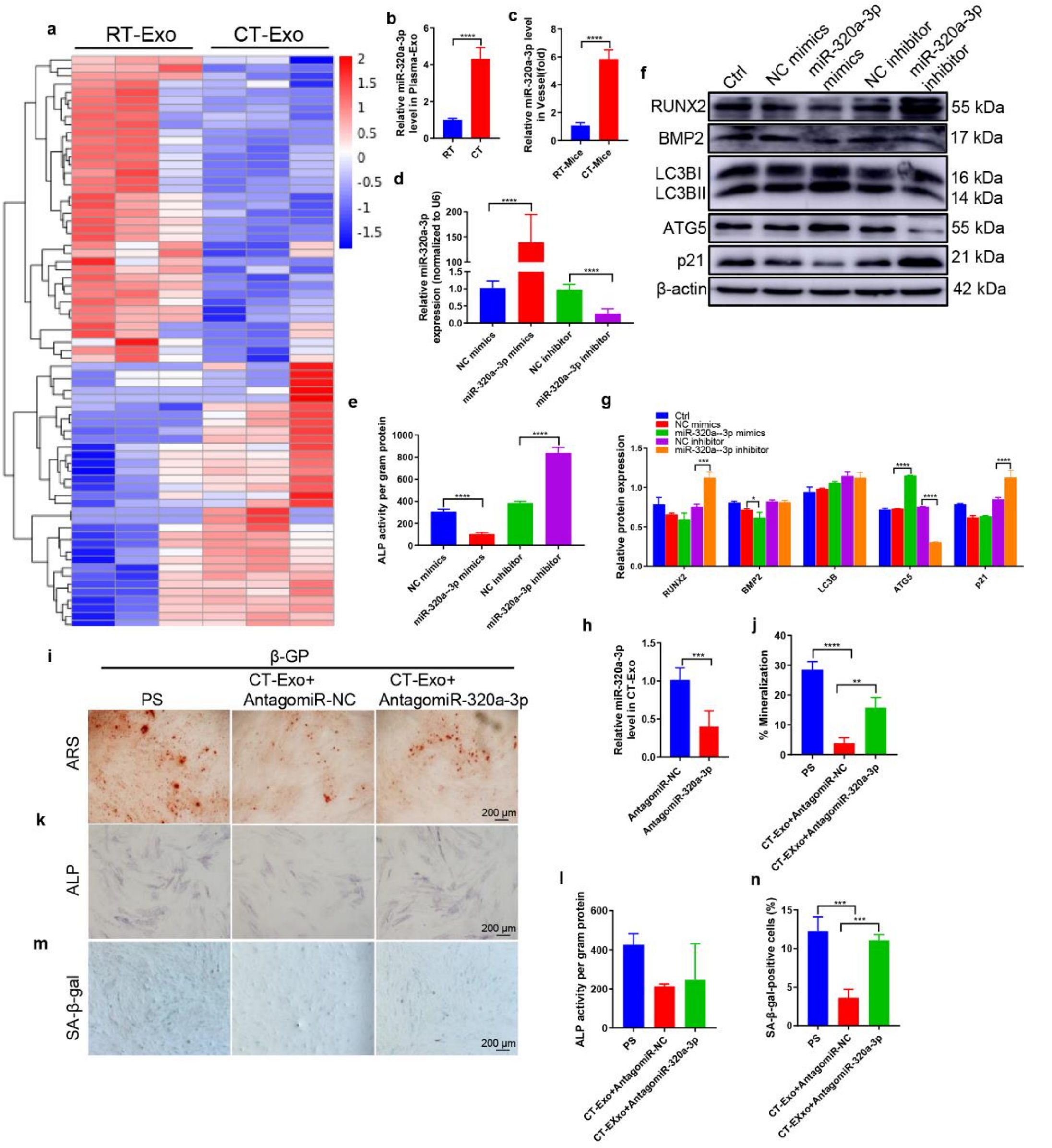

Fig. 5 miR-320a-3p antagonised osteogenic differentiation of VSMCs. (a) The heatmap shows the differentially expressed miRNAs (absolute fold change ≥ 1.5, $p < 0.05$) between CT-Exo and RT-Exo (n = 3 per group). (b) qRT-PCR analysis of miR-320a-3p expression in exosomes from the plasma of the RT or CT mice (n = 6). (c) qRT-

PCR analysis of miR-320a-3p expression in vessel s from RT or CT mice ($n = 6$). (d) qRT-PCR was performed to evaluate the expression of miR-320a-3p in VSMCs transfected with specific miR-320a-3p mimics or inhibitor ($n = 4$). (e) The ALP activity was evaluated by using specific kits in VSMCs transfected with specific miR-320a-3p mimics or inhibitors ($n = 4$). (f) Western blotting was performed to determine the protein expression of RUNX2, BMP2, LC3B, ATG5 and p21 in VSMCs transfected with specific miR-320a-3p mimics or inhibitors ($n = 4$). (g) The data are presented as densitometric ratios normalised to β-actin. (h) qRT-PCR analysis of miR-320a-3p expression in CT-Exo+AntagomiR-320a-3p ($n = 6$). ARS staining (i, j), ALP staining (k) and ALP activity (l) quantification of SA-β-gal-stained positive cells was shown (m, n). The black scale bar represents 200 μm ($n = 5$ per group). The PS group represents the positive control group with only β-GP treatment. The data are presented as the mean ± standard deviation. The data were analysed with one-way ANOVA with the Bonferroni *post hoc* test or the unpaired, two-tailed Student's t-test. *$p < 0.05$; **$p < 0.01$; ***$p < 0.001$; ****$p < 0.0001$.

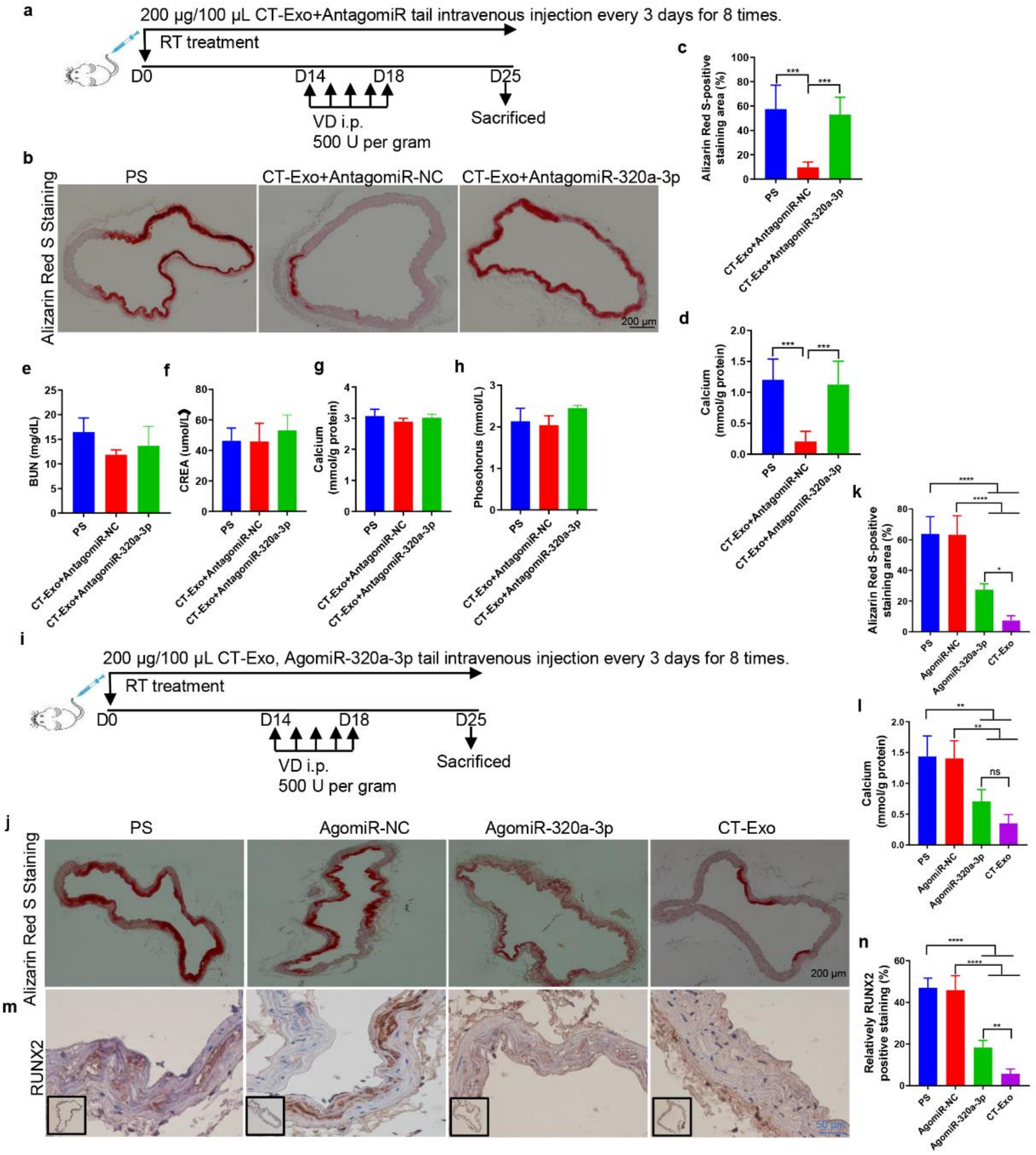

Fig. 6 miR-320a-3p effectively protected against MAC *in vivo* and its related biochemical indicators. (a) Experimental design of the VD-induced vascular calcification mouse model treated with PBS, CT-Exo+AntagomiR-NC or CT-Exo+AntagomiR-320a-3p by intravenous injection (n = 6 per group). ARS staining and quantitation (b, c) and vascular calcium content measurement (d). The black scale bar is 200 μm. Serum BUN (e), CREA (f), calcium (g) and phosphate (h) levels in mice with VD-induced vascular calcification (n = 6). (i) Experimental design of the VD-induced vascular calcification mouse model treated with PBS, AgomiR-NC or AgomiR-320a-3p by intravenous injection (n = 6). ARS staining and quantitation (j, k) and vascular calcium content measurement (l). RUNX2 expression in the thoracic aorta (m) and quantitation of positive staining area (n) are shown. The black scale bar is 200 μm and the blue scale bar is 50 μm. The PS group represents the control group with only β-GP treatment. The data are presented as the mean ± standard deviation. The data were analysed with one-way ANOVA with the Bonferroni *post hoc* test. ns > 0.05; **p*

< 0.05; **p < 0.01; ***p < 0.001 and ****p < 0.0001.

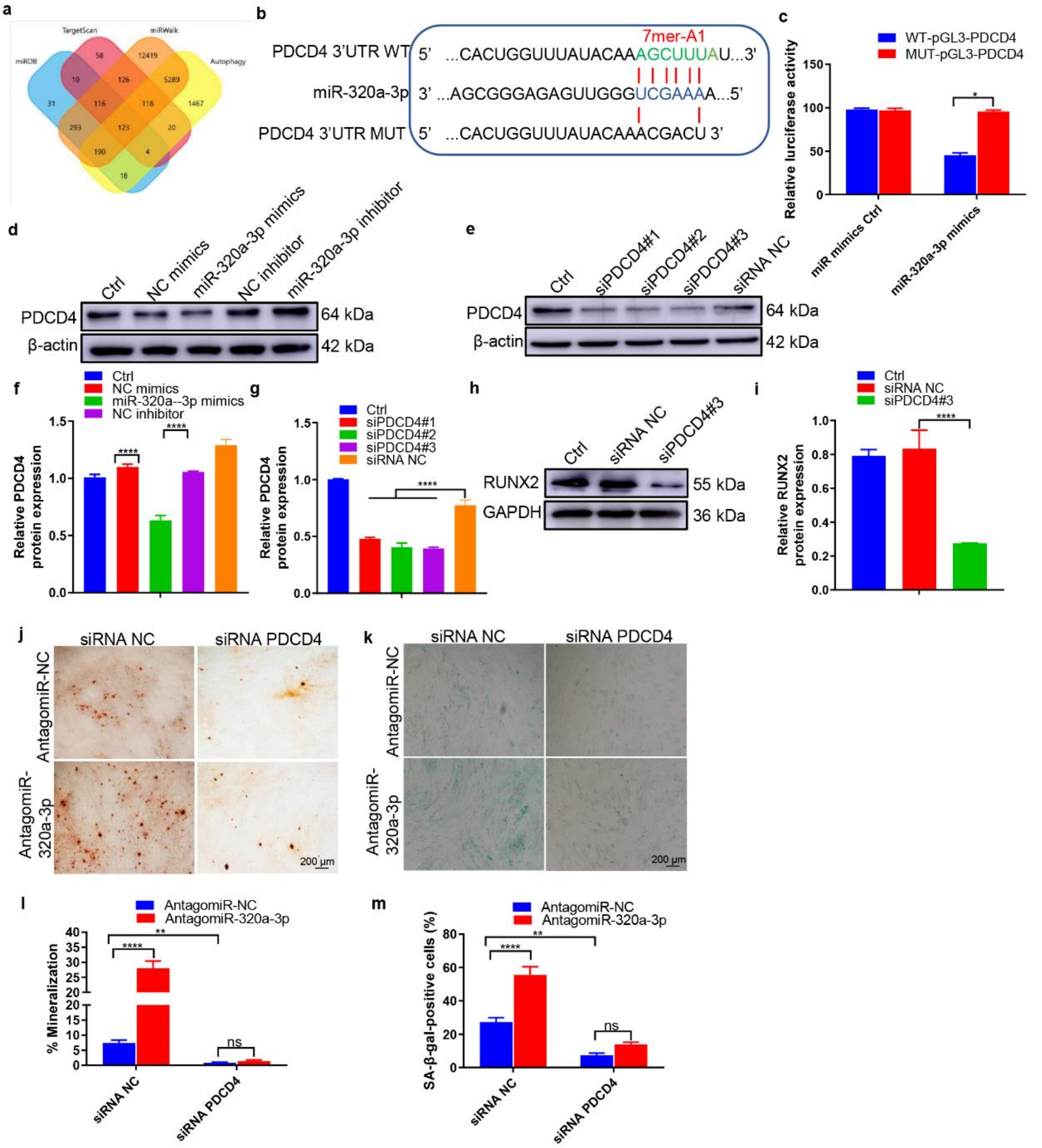

Fig. 7 PDCD4 was a direct target gene of miR-320a-3p and regulated VSMCs calcification. (a) A Venn diagram showing bioinformatics analysis of miR-320a-3p target genes. (b) Schematic representation of miR-320a-3p putative target sites in the PDCD4 3′-UTR and the alignment of miR-320a-3p with wild type and mutant PDCD4 3′-UTR showing pairing. (c) Luciferase reporter assays were performed using luciferase constructs carrying a wild type or mutant PDCD4 3′-UTR co-transfected into VSMCs with miR-320a-3p mimics compared with empty vector control. Firefly luciferase activity was normalised to *Renilla* luciferase activity. (d, f) PDCD4 protein expression in VSMCs transfected with miR-320a-3p mimics or miR-320a-3p inhibitor was determined by western blot (n = 4). (e and g) The efficiency of PDCD4 knockdown in VSMCs by siRNA was measured by western blot (n = 4). (h-i) RUNX2 expression was measured in the VSMCs treated with siPDCD4#3 or siRNA control (n = 4). (j) ARS staining in β-GP-treated VSMCs transfected with inhibitors of miR-320a-3p in the

presence or absence of PDCD4 siRNA for 28 days; representative micrographs are shown. (K) SA-β-gal staining was measured in VSMCs incubated with β-GP for 10 days. n = 4, the data are presented as the ratio of positive ARS (j) and SA-β-gal (m) staining area. The scale bar is 200 μm. The data are presented as the mean ± standard deviation. The data were analysed with one or two-way ANOVA with the Bonferroni *post hoc* test. ns > 0.05; **p* < 0.05; ***p* < 0.01; ****p* < 0.001 and *****p* < 0.0001.

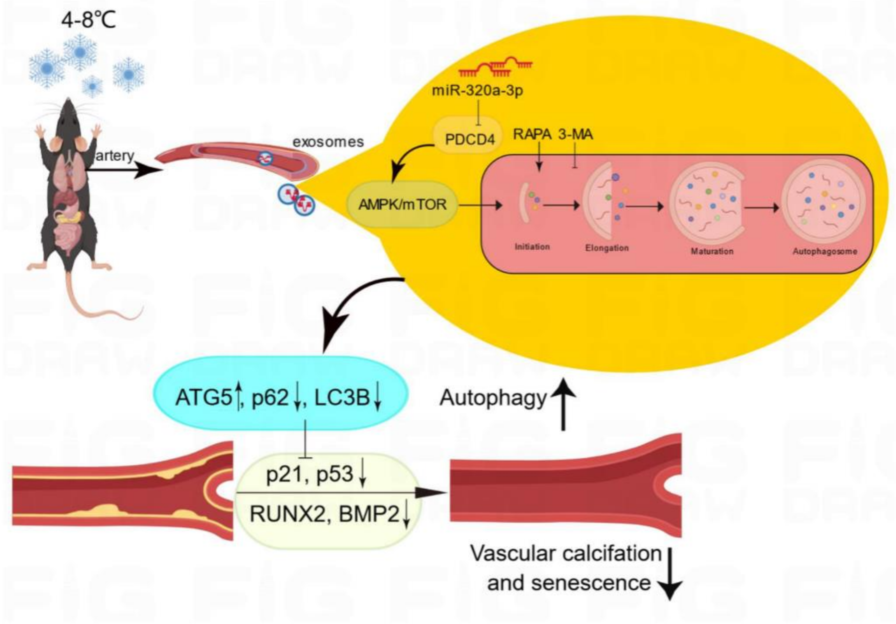

Fig. 8 CT-Exo enrichment of miR-320a-3p under CT exposure can protect against vascular calcification and senescence by activating autophagy through the AMPK/mTOR pathway. CT-Exo with the high expression of miR-320a-3p can be secreted from mice plasma exposed to a cold environment. PDCD4 was found to be a potential target of miR-320a-3p and to increase osteogenic differentiation and senescence of VSMCs. CT-Exo can activate AMPK/mTOR, a classical autophagy pathway and then activate the expression of autophagy proteins (LC3B and ATG5) and reduce the degradation of autophagy specific substrates (p62). Ultimately, this slow

down the level of senescence (p21 and p53) and decrease the level of calcification (RUNX2 and BMP2) of VSMCs.
