# Supplementary material for: Cold exposure protects against medial arterial calcification development via autophagy
Source: J Nanobiotechnology. 2023 Jul 17;21:226. doi: 10.1186/s12951-023-01985-1 (PMC10351118; doi:10.1186/s12951-023-01985-1)
Supplement: Supplementary file 4 — Supplementary Material 4 [file 12951_2023_1985_MOESM4_ESM.docx]

Supplementary Materials for

**Cold Exposure Protects Against Medial Arterial Calcification Development via Autophagy**

Fu-Xing-Zi Li *et al.*

*Corresponding author: Ling-Qing Yuan, allenylq@csu.edu.cn

**This PDF file includes:**

Figs. S1 to S9

Tables S1 to S2

**
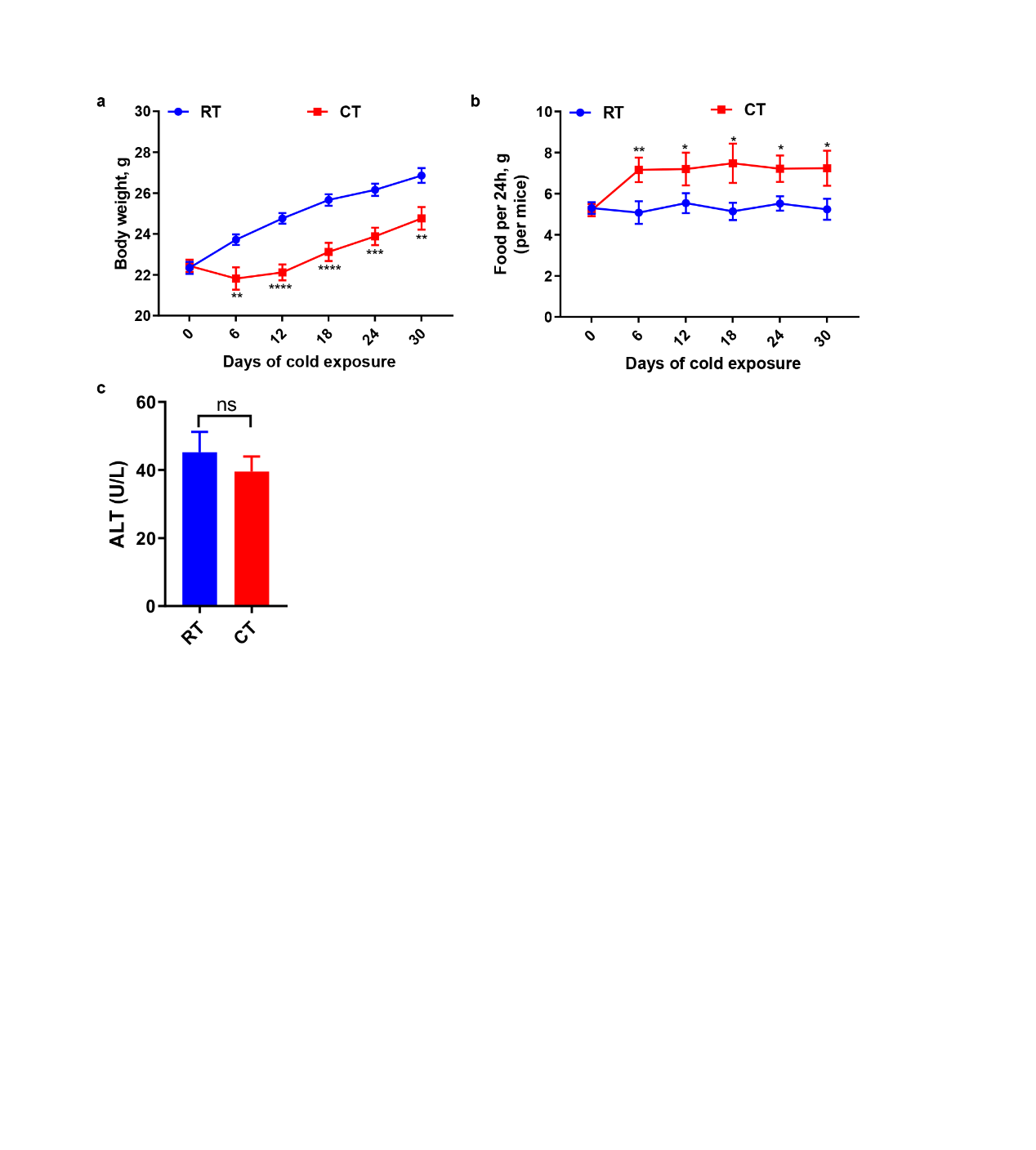
**

**Fig. s1** General appearance of RT and CT group mice after different temperature treatments. Body weight gain (a) and food consumption (b) of cold exposed mice and RT controls over 30 days. (c) Alanine aminotransferase (ALT). n = 6 per group; ns >0.05; **p* < 0.05; ***p* < 0.01; ****p* < 0.001; *****p* < 0.0001, unpaired Student’s test.


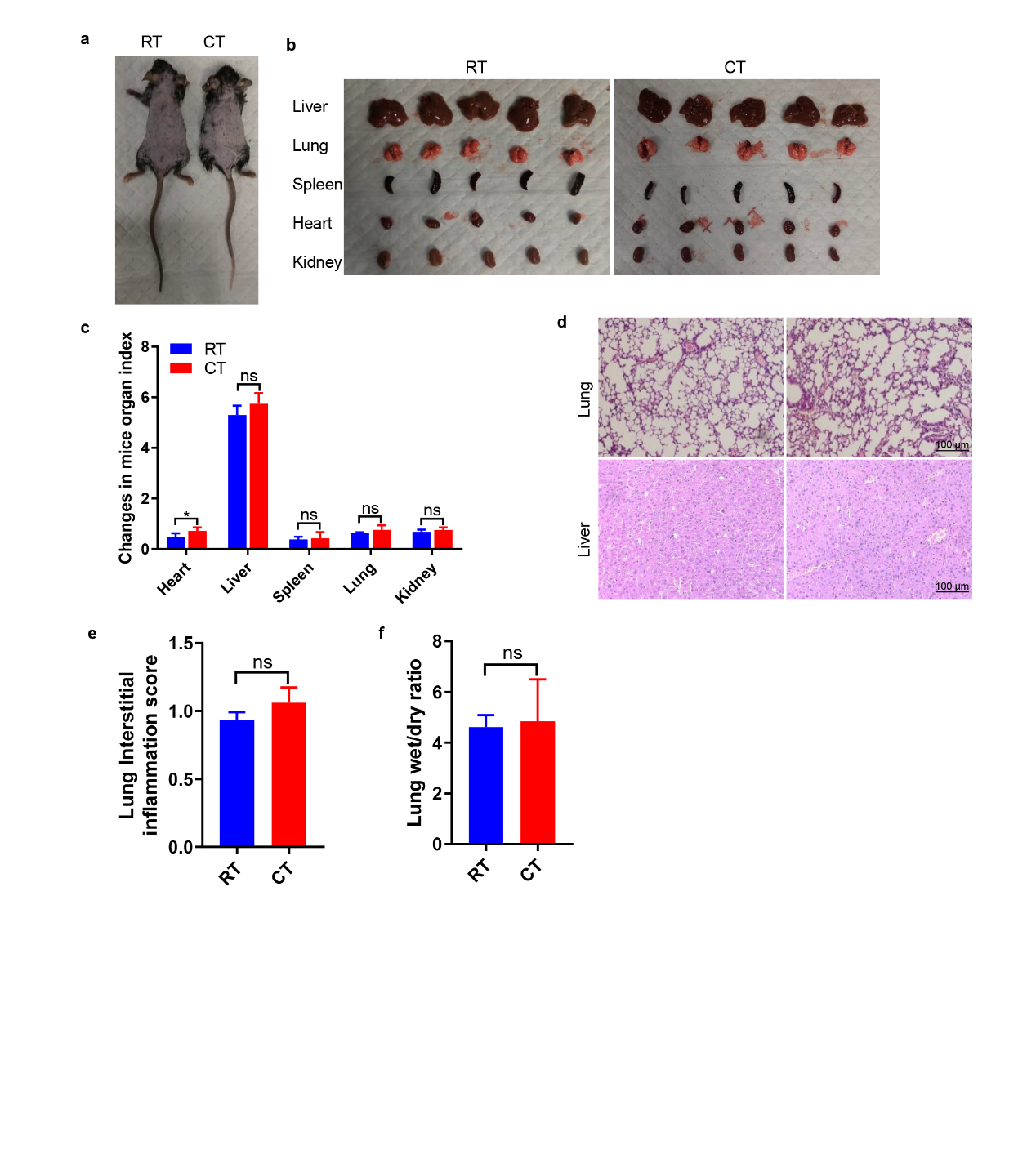


**Fig. s2** (a) Observe the appearance changes and destruction of skin tissue in mice after shaving. (b) General morphology of organs such as liver, lung, spleen, heart and kidney. (c) The effect of RT or CT on different organ indices in mice. (d) Representative histological sections of fixed lungs and livers were embedded in paraffin and stained with hematoxylin and eosin (H&E) (scale bars, 100 μm). (e) Evaluation of interstitial inflammation scores in lung slices of the RT and CT groups. (f) Wet/dry ratio in lung samples. n = 6 per group, ns > 0.05 and **p* < 0.05, unpaired t test with Welch's correction.


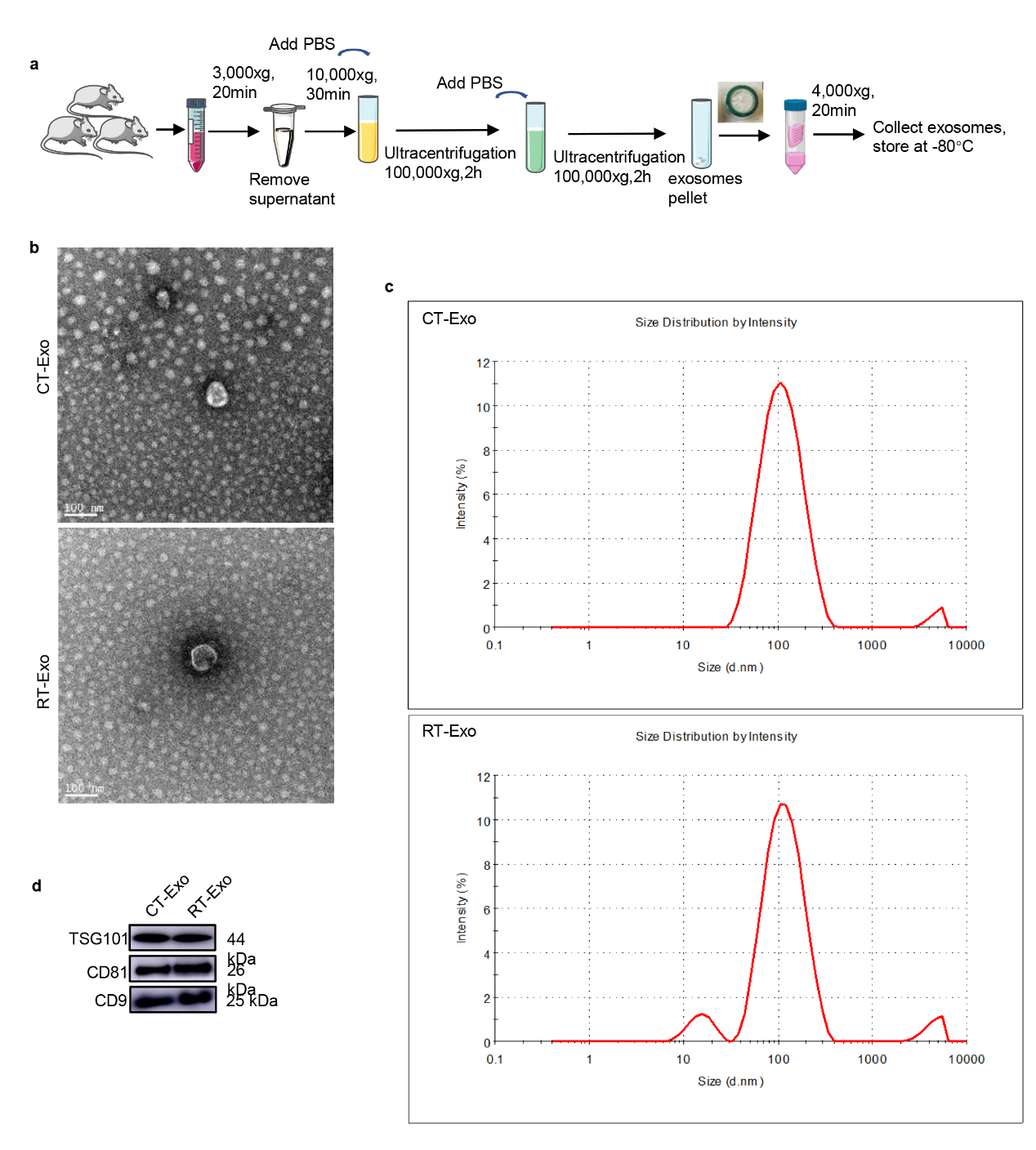


**Fig. s3** (a) Flow chart showing the extraction and isolation of plasma-derived exosomes. The purification procedure is based on differential ultracentrifugation. (b) TEM analysis of exosomes. The white scale bar is 100 nm. (c) Diameter distribution of exosomes. (d) Western blot of exosome-specific proteins TSG101, CD81 and CD9, which are abundant in CT-Exo and RT-Exo.


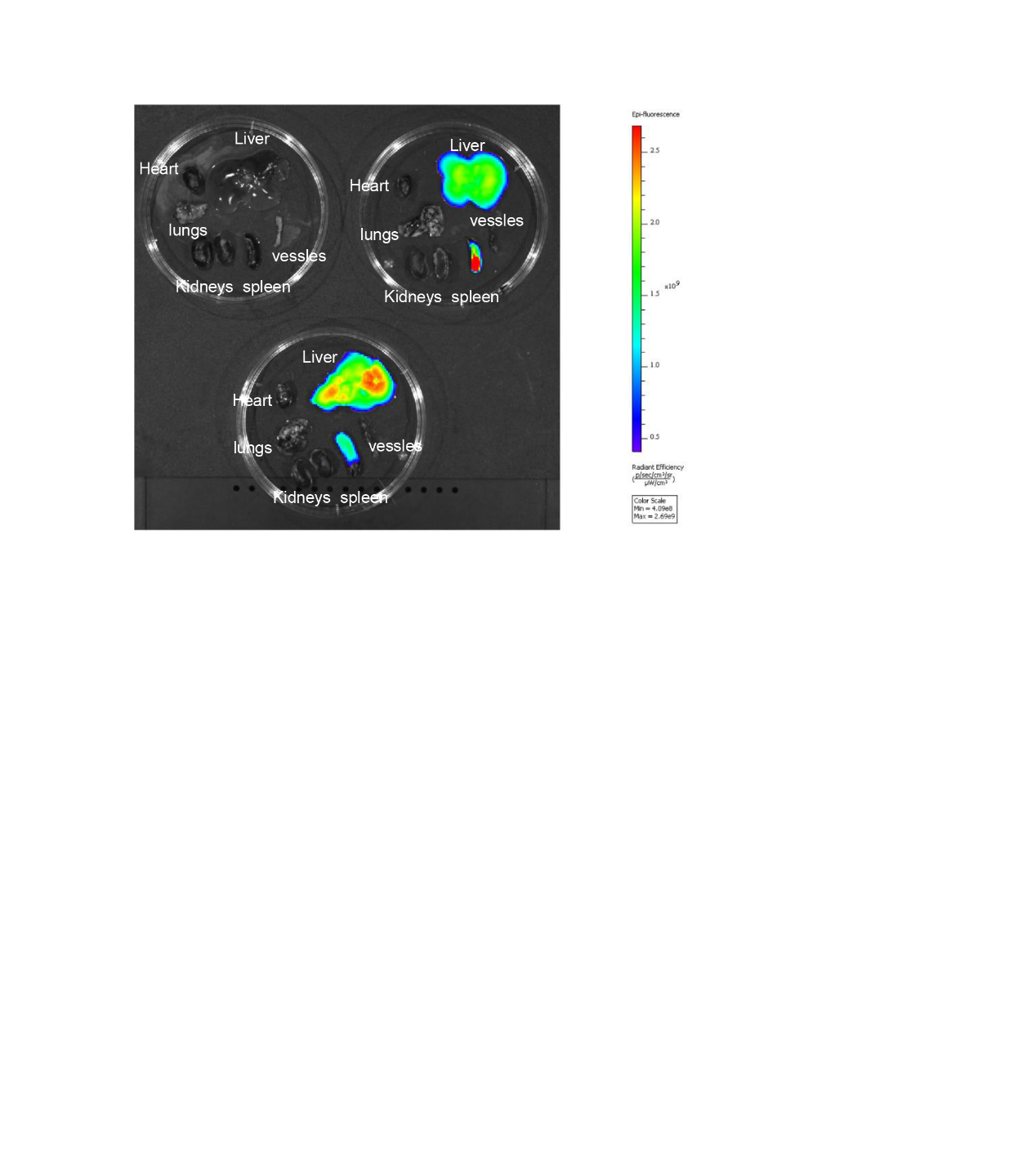


**Fig. s4** Fluorescence signals were detected in the organs of mice after execution (n = 3 per group).


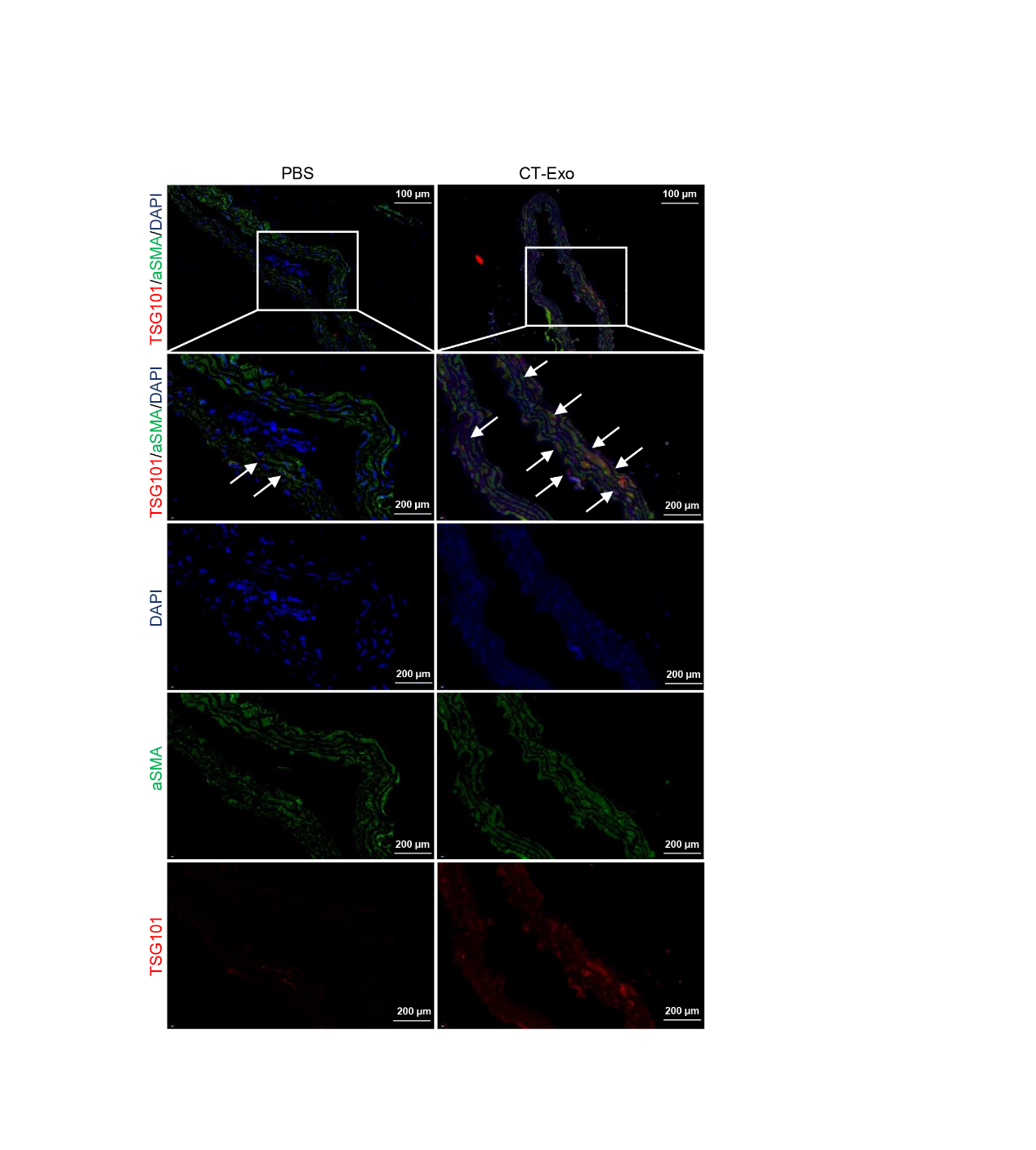


**Fig. s5** Representative fluorescence micrograph showed the CT-Exo marker TSG101 (red) and smooth muscle marker α-SMA (green) in thoracic aortic sections (n = 3 per group).

**
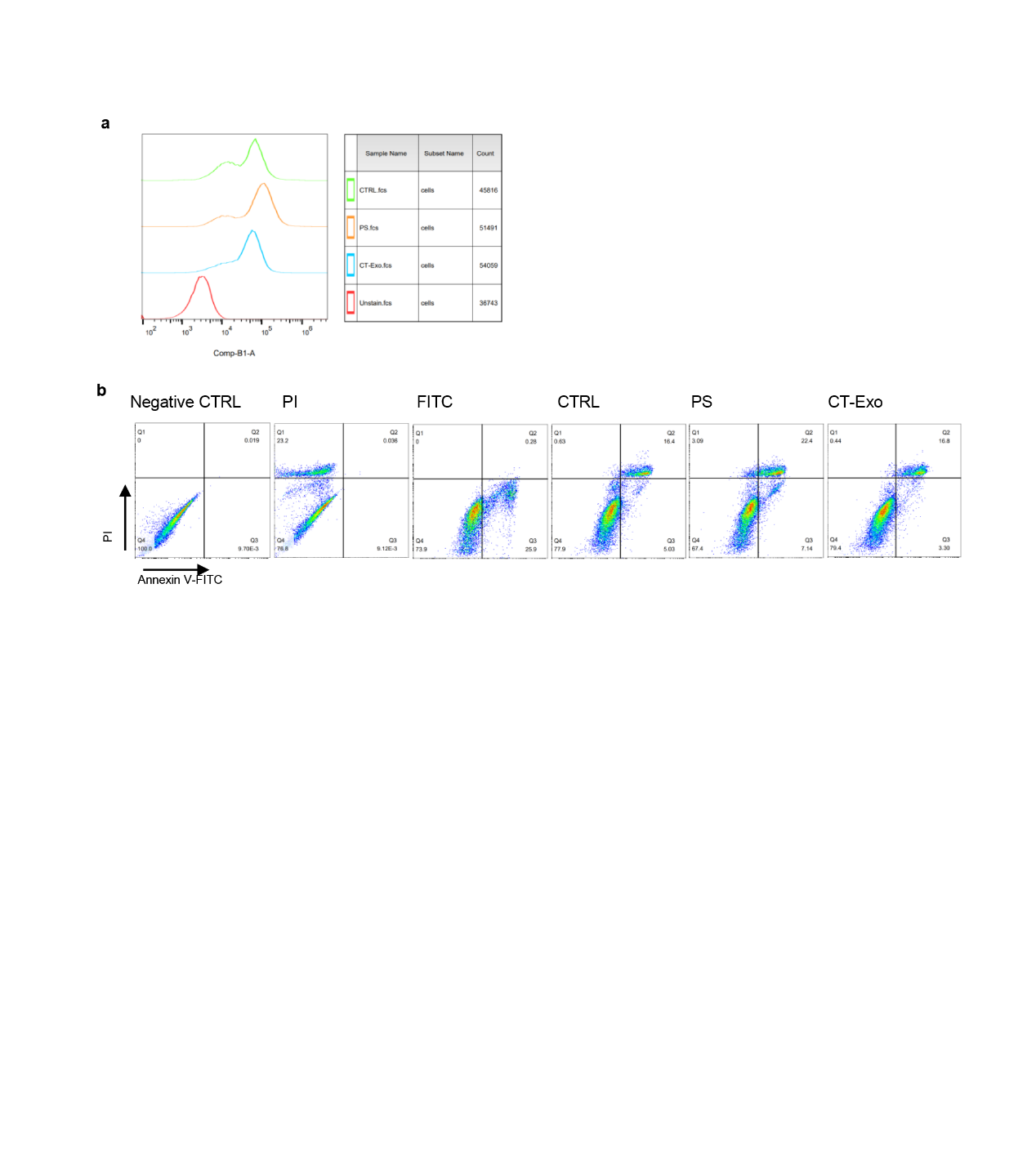
**

**Fig. s6** CT-Exo exerted effects on the ROS level and apoptosis of VSMCs. (a) DCFH-DA measures intracellular ROS production by flow cytometry. (b) Representative flow cytometric analysis of Annexin V-FITC/PI-stained VSMCs receiving different treatments for 3 days (n = 4 per group).


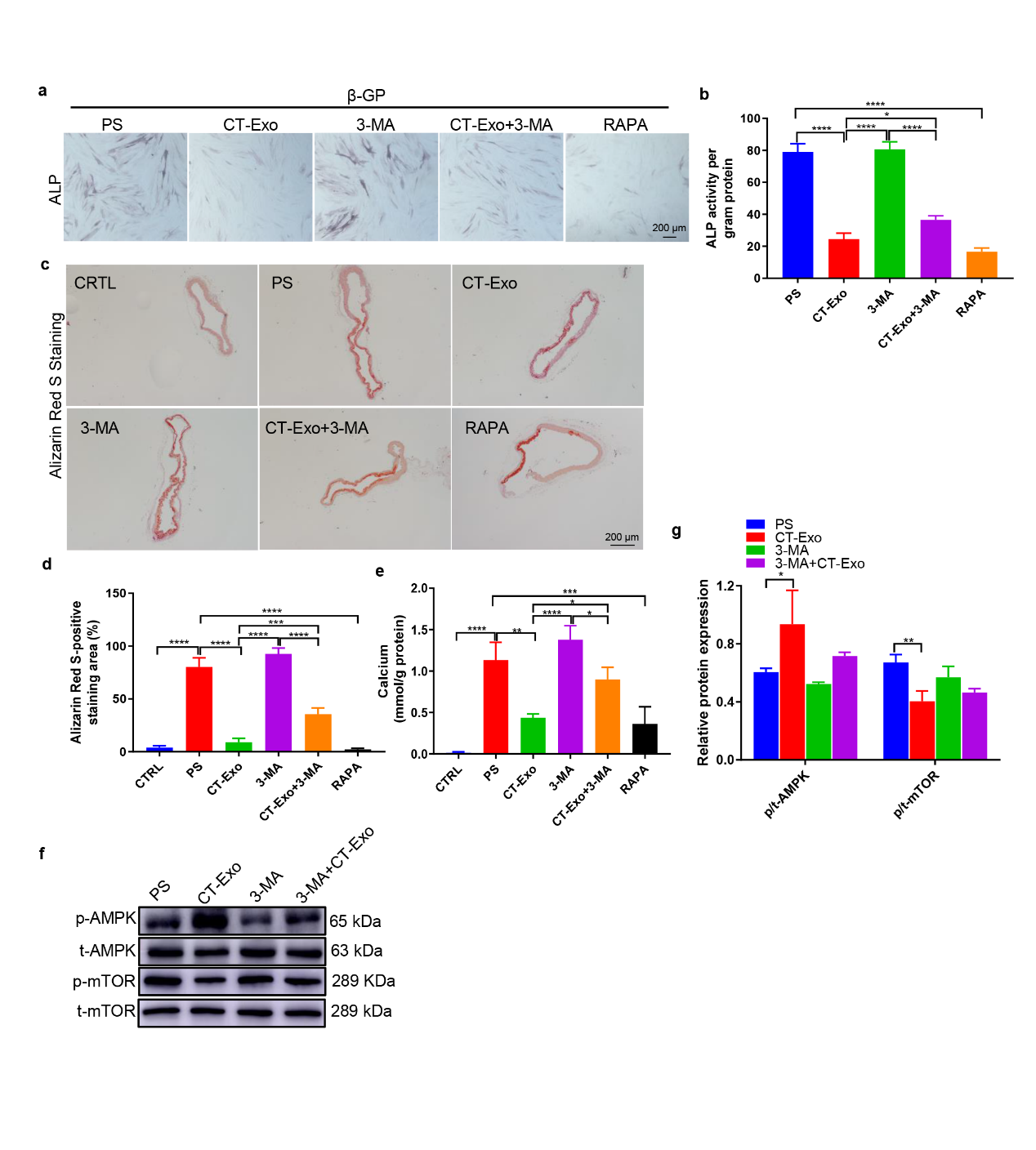


**Fig. s7** The autophagy inhibitor 3-MA, through the AMPK/mTOR signalling pathway, effectively inhibited the ability of CT-Exo to promote osteogenic differentiation. (a) Representative images of ALP staining of VSMCs that had been pre-treated with the indicated concentrations of 3-MA or rapamycin for 30 min and then incubated with β-GP for 14 days (n = 5 per group). The scale bar is 200 μm. (b) Quantitative analysis of the ALP activity. (c, d) ARS staining showing calcified aorta from CRTL, PS, CT-Exo, 3-MA, CT-Exo+3-MA and RAPA mice (n = 5 per group). The black scale bar is 200 μm. (e) Vascular calcium content measurement. (f) The expression of p/t-AMPK and p/t-mTOR was determined with western blot in calcified VSMCs treated with CT-Exo, 3-MA or 3-MA+CT-Exo (n = 4 per group). (g) Quantitative analysis of western blotting results. The CTRL group represented the negative control group with only PBS treatment. The PS group represented the positive control group with only β-GP treatment. The data are expressed as the mean ± standard deviation. The data were analysed with one-way ANOVA with the Bonferroni *post hoc* test or the unpaired, two-tailed Student’s t-test. **p* < 0.05; ***p* < 0.01; ****p* < 0.001; *****p* < 0.0001.


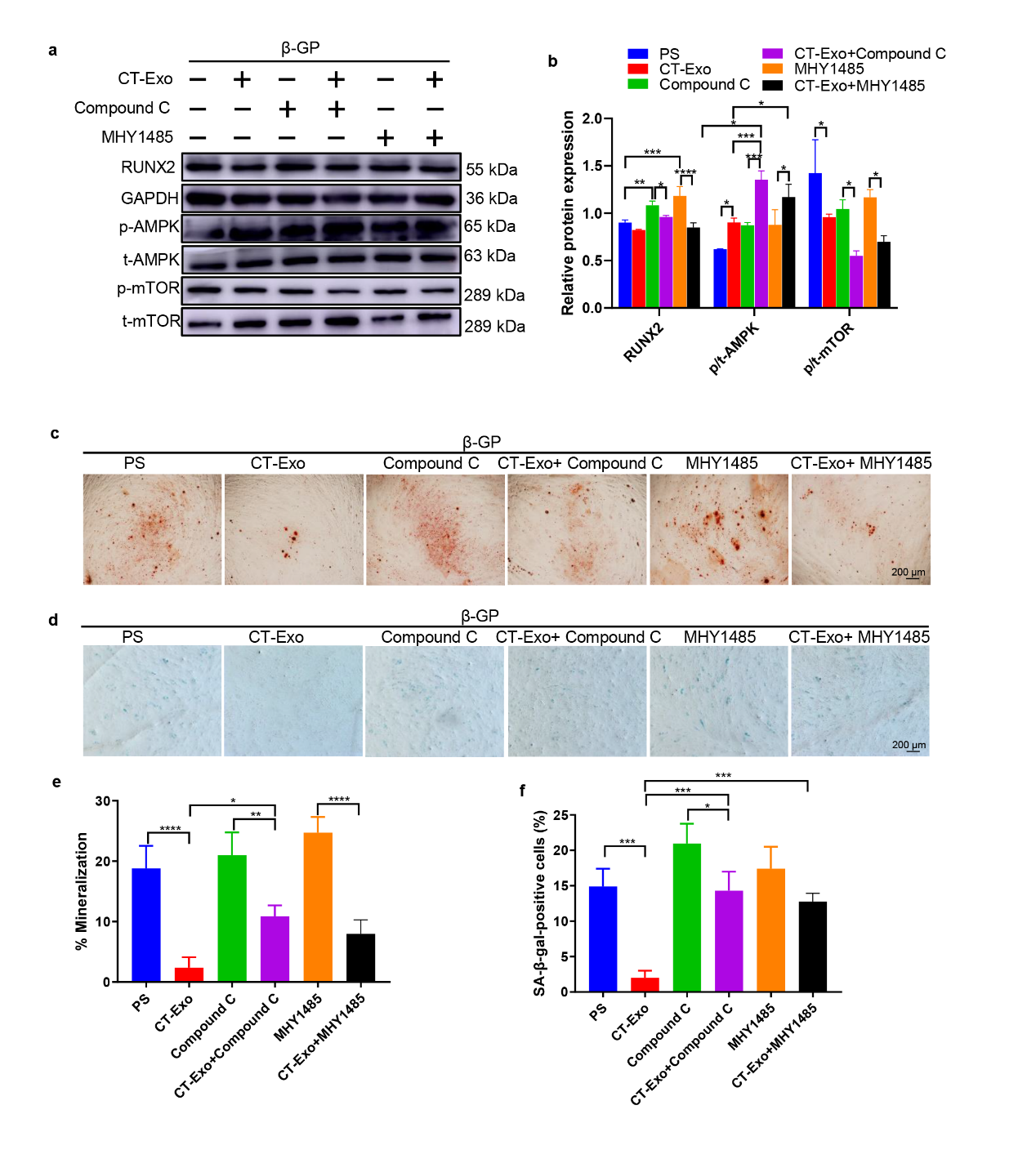


**Fig. s8** The AMPK/mTOR signalling pathway mediated defensive roles of CT-Exo on calcification/aging of VSMCs. (a) Expression of p-mTOR and p-AMPK in the β-GP-induced VSMCs treated with Compound C or MHY1485 were analysed by western blot (n = 4 per group). (b) The data are presented as densitometric ratios of RUNX2/GAPDH, p/t-mTOR and p/t-AMPK respectively. (c, d) Representative micrographs of ARS and SA-β-gal staining view were shown (n = 5 per group). (e, f) The data are presented as ratio of positive staining area, shown as the mean ± standard deviation. The data were analysed with one-way ANOVA with the Bonferroni *post hoc* test or the unpaired, two-tailed Student’s t-test. **p* < 0.05; ***p* < 0.01; ****p* < 0.001; *****p* < 0.0001.


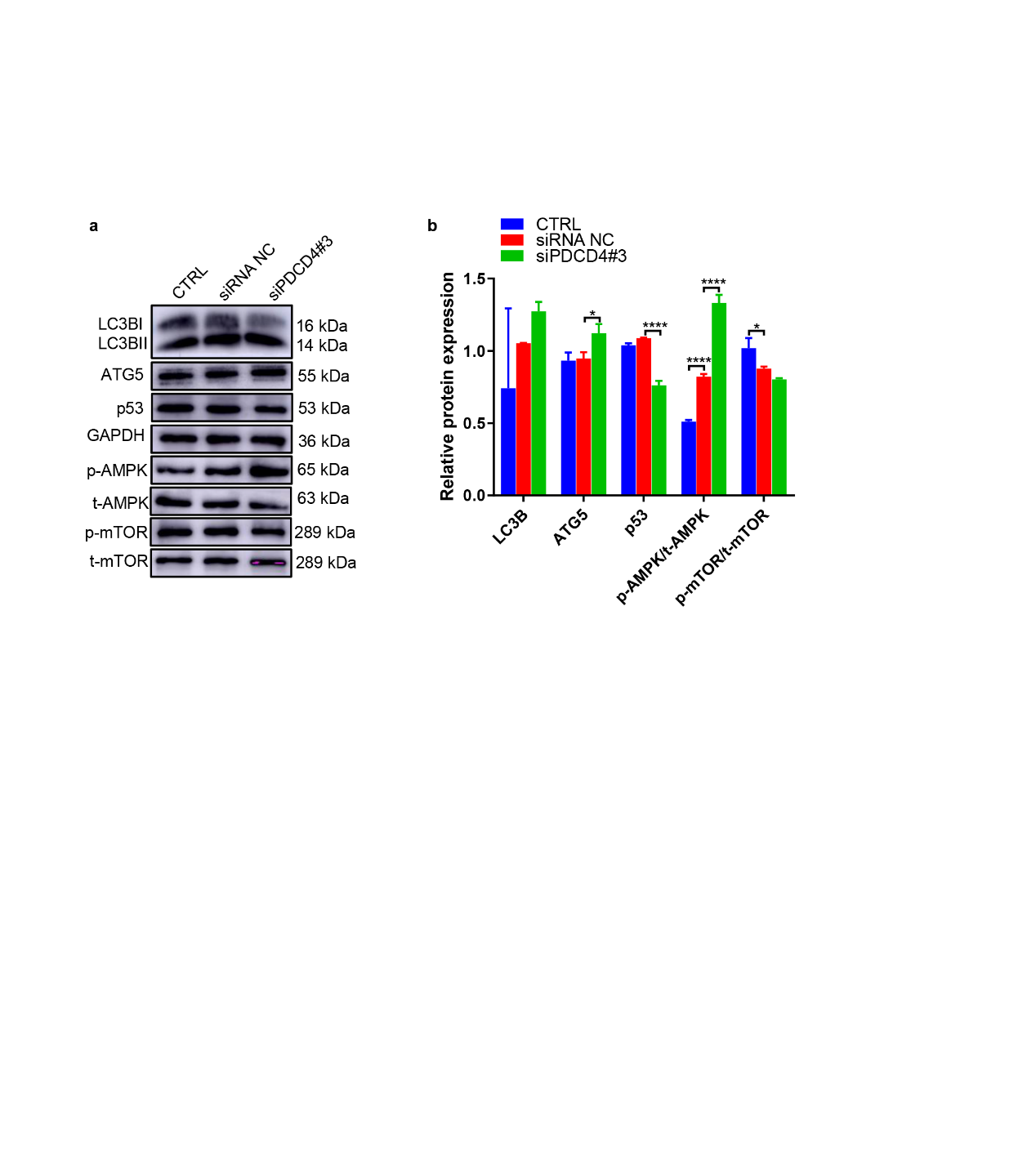


**Fig. s9** siPDCD4 can activate the AMPK/mTOR signalling pathway to promote VSMCs autophagy. Western blot analysis (a) and quantification (b) of LC3B, ATG5, p53, p/t-AMPK and p/t-mTOR in VSMCs treated with siPDCD#3 or siRNA control (n = 4 per group). The CTRL group represented the negative control group with only PBS treatment. The data are presented as the mean ± standard deviation. The data were analysed one-way ANOVA with the Bonferroni *post hoc* test. **p* < 0.05; *****p* < 0.0001.

**Table S1**

RNA oligonucleotide sequences.

| **RNA oligos** | **Sequences** |
| --- | --- |
| mimics-NC  inhibitor-NC | Sense: 5’- UUCUCCGAACGUGUCACGUTT -3’  Anti-sense: 5’- ACGUGACACGUUCGGAGAATT -3’  Sense: 5’- CAGUACUUUUGUGUAGUACAA -3’ |
| miR-320a-3p mimics  miR-320a-3p inhibitor  agomir-NC  Hsa-miR-320a-3p agomir  antagomir-NC | Sense: 5’- AAAAGCUGGGUUGAGAGGGCGA -3’  Anti-sense: 5’- GCCCUCUCAACCCAGCUUUUUU -3’  Sense: 5’- UCGCCCUCUCAACCCAGCUUUU -3’  Sense: 5’- UCUCCGAACGUGUCACGUTT -3’  Anti-sense: 5’- ACGUGACACGUUCGGAGAATT -3’  Sense: 5’- AAAAGCUGGGUUGAGAGGGCGA -3’  Anti-sense: 5’- GCCCUCUCAACCCAGCUUUUUU -3’  Sense: 5’- CAGUACUUUUGUGUAGUACAA -3’ |
| Hsa-miR-320a-3p antagomir | Sense: 5’- UCGCCCUCUCAACCCAGCUUUU -3’ |
| siRNA-NC | Sense: 5’- UUCUCCGAACGUGUCACGUTT -3’  Anti-sense: 5’- ACGUGACACGUUCGGAGAATT -3’ |
| si-PDCD4#1  (PDCD4-Homo-533) | Sense: 5’- CGCCCUUAGAAGUGGAUUATT -3’  Anti-sense: 5’- UAAUCCACUUCUAAGGGCGTT -3’ |
| si-PDCD4#2  (PDCD4-Homo-966) | Sense: 5’- GGGACAGUAAUGAGCACAATT -3’  Anti-sense: 5’- UUGUGCUCAUUACUGUCCCTT -3’ |
| si-PDCD4#3  (PDCD4-Homo-1340) | Sense: 5’- GGAACUGGAAGUACCUCAUTT -3’  Anti-sense: 5’- AUGAGGUACUUCCAGUUCCTT -3’ |

**Table S2**

| **Differentially expressed miRNAs in CT-Exo and RT-Exo** | | |
| --- | --- | --- |
| above 1.5-fold change (CT-Exo/RT-Exo) |  |  |
| Systematic Name | Fold Change | P Value |
| mmu-miR-351-5p | 94.14345058 | 0.001543586 |
| mmu-miR-6538 | 61.42467529 | 0.023048967 |
| novel_382 | 56.48546031 | 0.014468841 |
| mmu-let-7e-3p | 56.30284961 | 0.025739511 |
| mmu-miR-674-5p | 55.09039783 | 0.016790423 |
| mmu-miR-122-3p | 54.47332056 | 0.047083333 |
| mmu-miR-700-5p | 52.84191248 | 0.037570328 |
| mmu-miR-139-3p | 31.77955771 | 0.031028902 |
| mmu-miR-132-3p | 15.52947479 | 0.001265178 |
| mmu-miR-484 | 7.101759389 | 0.020935936 |
| mmu-miR-423-3p | 7.100413899 | 0.00036135 |
| mmu-miR-326-3p | 6.075044388 | 0.029537992 |
| mmu-miR-200b-5p | 6.064892227 | 0.03727916 |
| mmu-miR-361-3p | 5.726686309 | 0.001844238 |
| mmu-miR-1964-3p | 5.00455166 | 0.025783004 |
| mmu-miR-205-5p | 4.971517543 | 0.019277103 |
| mmu-miR-125b-5p | 4.732869234 | 0.00405241 |
| mmu-miR-129b-3p | 4.464908874 | 0.018266228 |
| mmu-miR-129-5p | 4.457498999 | 0.018349179 |
| mmu-miR-25-3p | 4.239275669 | 0.001349913 |
| mmu-miR-3473h-5p | 4.136969691 | 0.033216441 |
| mmu-miR-30f | 3.416446609 | 0.021327587 |
| mmu-miR-541-5p | 3.22453363 | 0.024792296 |
| mmu-miR-1198-5p | 3.129684569 | 0.004581151 |
| mmu-miR-320-3p | 2.970331662 | 0.016394786 |
| mmu-miR-222-3p | 2.924196172 | 0.016064421 |
| mmu-miR-148a-3p | 2.572280033 | 0.014623624 |
| mmu-miR-10a-5p | 2.315407853 | 0.045708164 |
| mmu-miR-22-3p | 2.289075786 | 0.021701031 |
| mmu-miR-486a-3p | 2.247090095 | 0.048455547 |
| mmu-miR-486b-3p | 2.233448607 | 0.049933101 |
| mmu-miR-3074-5p | 2.119647107 | 0.047467127 |
| mmu-miR-30d-5p | 2.119353461 | 0.043618173 |
|  |  |  |
| below 1/1.5-fold change (CT-Exo/RT-Exo) |  |  |
| Systematic Name | Fold Change | P Value |
| mmu-miR-21a-5p | 0.455624884 | 0.038703572 |
| mmu-miR-429-3p | 0.439845565 | 0.045975995 |
| mmu-miR-374b-5p | 0.418011421 | 0.036920032 |
| mmu-miR-374c-3p | 0.418011421 | 0.036920032 |
| mmu-miR-455-5p | 0.417683968 | 0.035650318 |
| mmu-miR-20a-5p | 0.391811503 | 0.028183596 |
| mmu-miR-17-5p | 0.387886436 | 0.021436253 |
| mmu-miR-199b-5p | 0.382965729 | 0.044599542 |
| mmu-miR-146a-5p | 0.382779982 | 0.013202416 |
| mmu-miR-499-5p | 0.380337471 | 0.044347646 |
| mmu-miR-574-5p | 0.373117938 | 0.019734011 |
| mmu-miR-199a-3p | 0.358798404 | 0.009561352 |
| mmu-miR-1b-5p | 0.356836899 | 0.039578254 |
| mmu-miR-1a-3p | 0.356746148 | 0.039542379 |
| mmu-miR-106b-5p | 0.356702402 | 0.020516121 |
| mmu-miR-26b-5p | 0.350408557 | 0.007046242 |
| mmu-let-7e-5p | 0.349807811 | 0.014449685 |
| mmu-miR-126a-5p | 0.329061765 | 0.014901413 |
| mmu-miR-126b-3p | 0.329061765 | 0.014901413 |
| mmu-miR-30b-3p | 0.31874273 | 0.009355289 |
| mmu-miR-379-5p | 0.300828787 | 0.002260747 |
| mmu-miR-1839-5p | 0.277076994 | 0.009353765 |
| mmu-let-7g-5p | 0.258268459 | 0.00169423 |
| mmu-miR-18a-5p | 0.257652905 | 0.048768434 |
| mmu-miR-200a-3p | 0.253255719 | 0.002519558 |
| mmu-let-7d-5p | 0.252415676 | 0.002712576 |
| mmu-miR-127-5p | 0.250162843 | 0.040952059 |
| mmu-miR-144-5p | 0.246208594 | 0.001025716 |
| mmu-miR-3071-3p | 0.233785141 | 0.004655797 |
| mmu-miR-7b-5p | 0.22772835 | 0.002973106 |
| mmu-miR-136-5p | 0.224000751 | 0.003643359 |
| mmu-miR-15b-3p | 0.197259205 | 0.000839446 |
| mmu-miR-669a-3p | 0.169599839 | 0.03398022 |
| mmu-miR-206-3p | 0.168045084 | 0.000640255 |
| mmu-miR-7073-5p | 0.15249098 | 0.033920041 |
| mmu-mR-329-3p | 0.145051596 | 0.037213323 |
| mmu-miR-152-3p | 0.106641642 | 8.28E-06 |
| mmu-miR-376b-5p | 0.071833174 | 0.008009447 |
|  |  |  |
