# Supplementary material for: Cold exposure protects against medial arterial calcification development via autophagy
Source: J Nanobiotechnology. 2023 Jul 17;21:226. doi: 10.1186/s12951-023-01985-1 (PMC10351118; doi:10.1186/s12951-023-01985-1)
Supplement: Supplementary file 5 — Supplementary Material 5 [file 12951_2023_1985_MOESM5_ESM.pdf]

Supplementary Materials for

Cold Exposure Protects Against Medial Arterial Calcification Development via Autophagy

Fu-Xing-Zi Li *et al.*

*Corresponding author: Ling-Qing Yuan, allenylq@csu.edu.cn

This PDF file includes:

Figs. S1 to S9
Tables S1 to S2

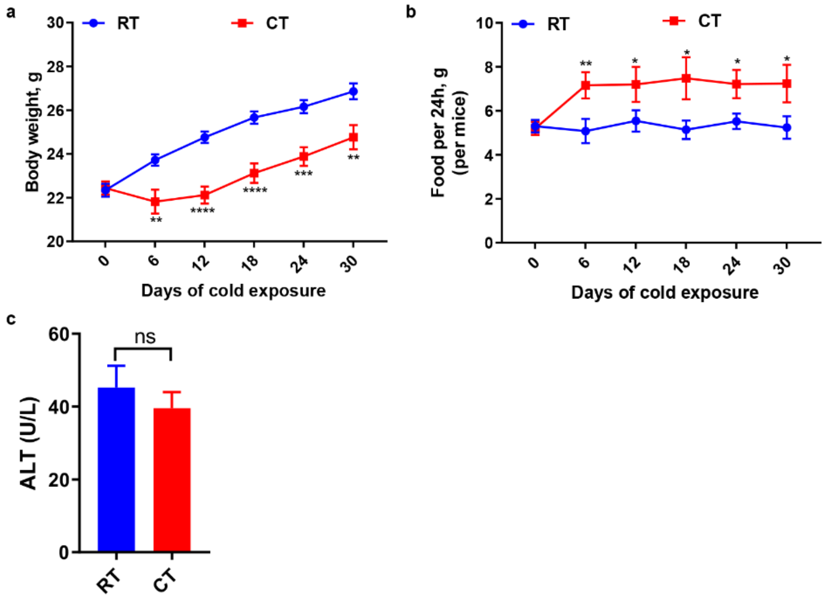

Fig. s1 General appearance of RT and CT group mice after different temperature treatments. Body weight gain (a) and food consumption (b) of cold exposed mice and RT controls over 30 days. (c) Alanine aminotransferase (ALT). n = 6 per group; ns >0.05; *$p < 0.05$; **$p < 0.01$; ***$p < 0.001$; ****$p < 0.0001$, unpaired Student's test.

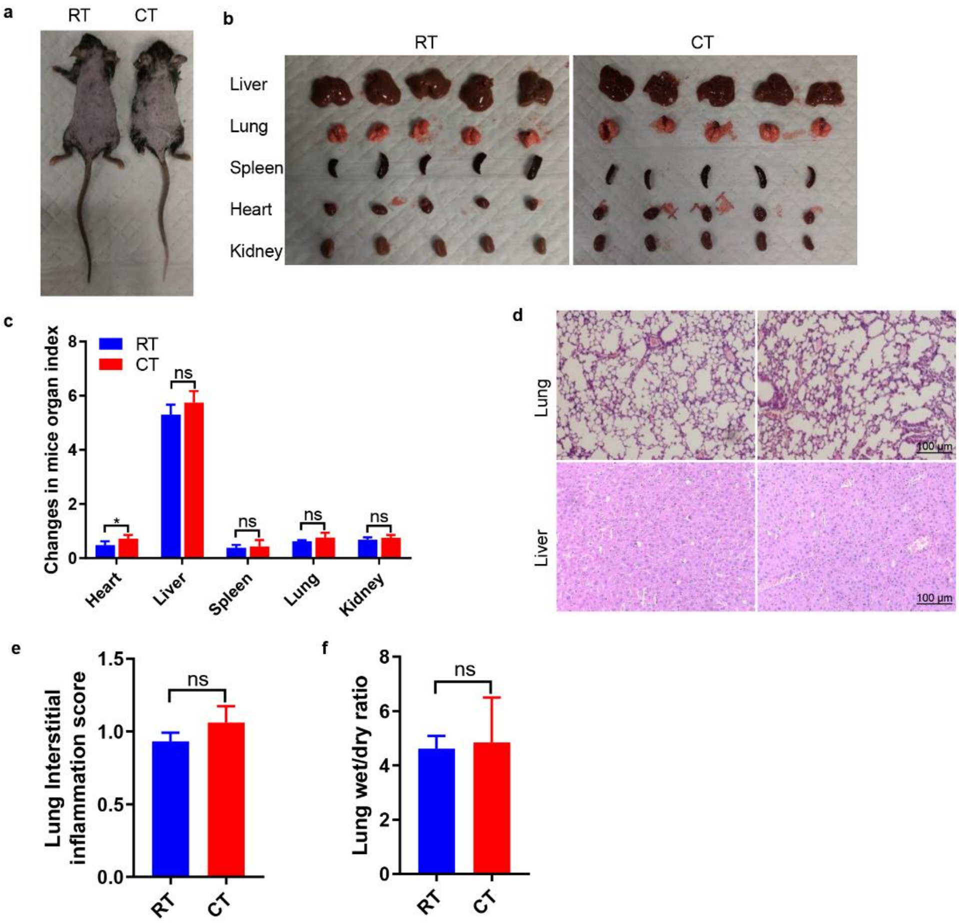

Fig. s2 (a) Observe the appearance changes and destruction of skin tissue in mice after shaving. (b) General morphology of organs such as liver, lung, spleen, heart and kidney. (c) The effect of RT or CT on different organ indices in mice. (d) Representative histological sections of fixed lungs and livers were embedded in paraffin and stained with hematoxylin and eosin (H&E) (scale bars, 100 μm). (e) Evaluation of interstitial inflammation scores in lung slices of the RT and CT groups. (f) Wet/dry ratio in lung samples. n = 6 per group, ns > 0.05 and *$p < 0.05$, unpaired t test with Welch's correction.

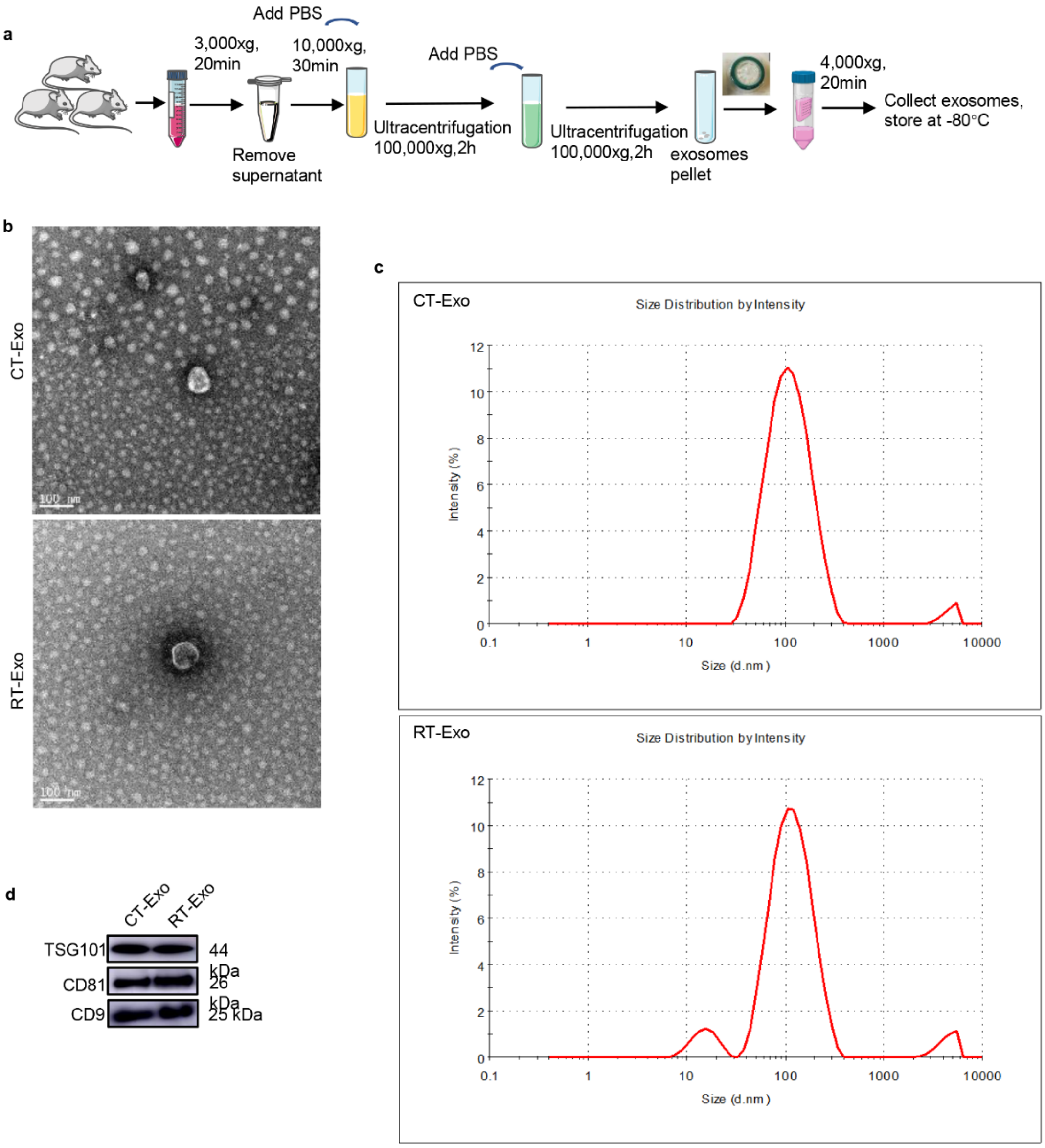

Fig. s3 (a) Flow chart showing the extraction and isolation of plasma-derived exosomes. The purification procedure is based on differential ultracentrifugation. (b) TEM analysis of exosomes. The white scale bar is 100 nm. (c) Diameter distribution of exosomes. (d) Western blot of exosome-specific proteins TSG101, CD81 and CD9, which are abundant in CT-Exo and RT-Exo.

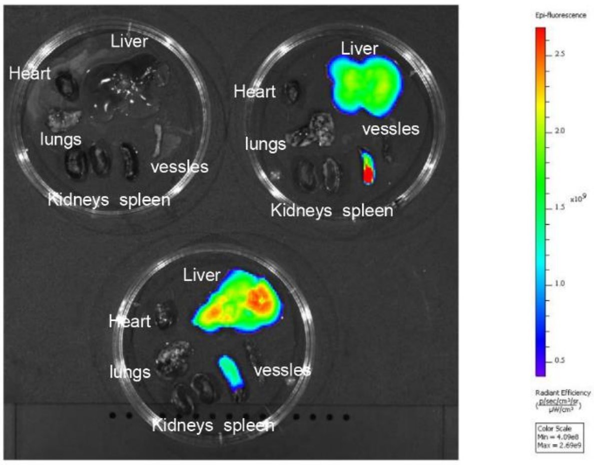

Fig. s4 Fluorescence signals were detected in the organs of mice after execution (n = 3 per group).

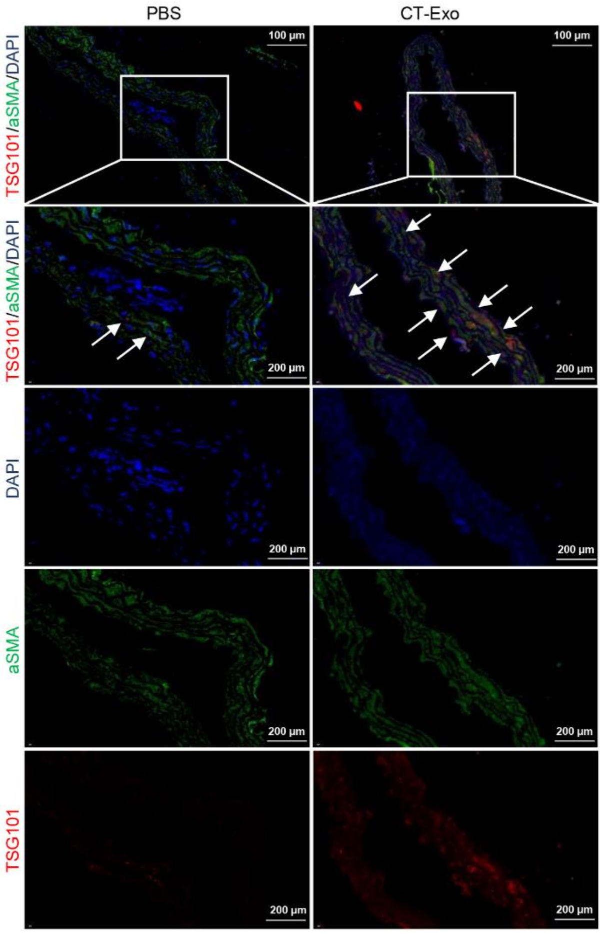

Fig. s5 Representative fluorescence micrograph showed the CT-Exo marker TSG101 (red) and smooth muscle marker α-SMA (green) in thoracic aortic sections (n = 3 per group).

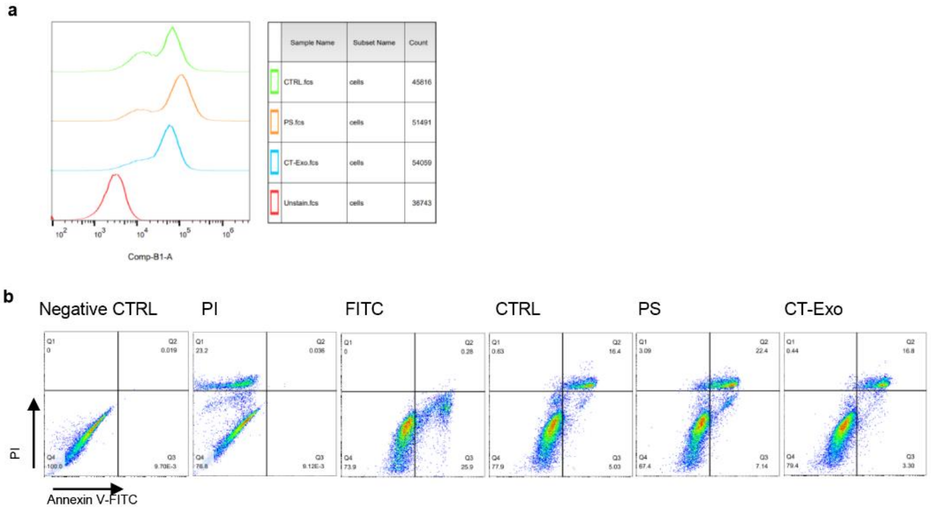

Fig. s6 CT-Exo exerted effects on the ROS level and apoptosis of VSMCs. (a) DCFH-DA measures intracellular ROS production by flow cytometry. (b) Representative flow cytometric analysis of Annexin V-FITC/PI-stained VSMCs receiving different treatments for 3 days (n = 4 per group).

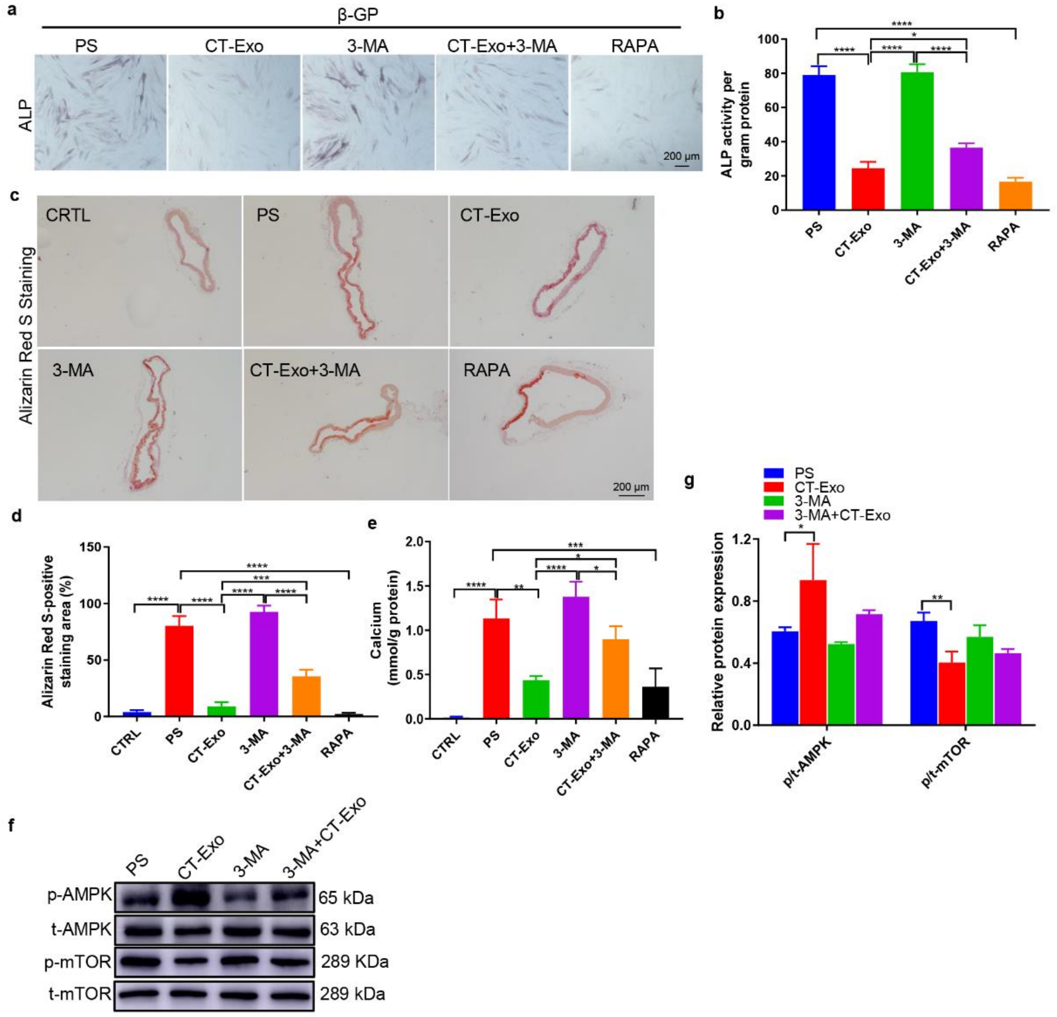

Fig. s7 The autophagy inhibitor 3-MA, through the AMPK/mTOR signalling pathway, effectively inhibited the ability of CT-Exo to promote osteogenic differentiation. (a) Representative images of ALP staining of VSMCs that had been pre-treated with the indicated concentrations of 3-MA or rapamycin for 30 min and then incubated with β-GP for 14 days (n = 5 per group). The scale bar is 200 μm. (b) Quantitative analysis of the ALP activity. (c, d) ARS staining showing calcified aorta from CRTL, PS, CT-Exo, 3-MA, CT-Exo+3-MA and RAPA mice (n = 5 per group). The black scale bar is 200 μm. (e) Vascular calcium content measurement. (f) The expression of p/t-AMPK and p/t-mTOR was determined with western blot in calcified VSMCs treated with CT-Exo, 3-MA or 3-MA+CT-Exo (n = 4 per group). (g) Quantitative analysis of western blotting results. The CTRL group represented the negative control group with only PBS treatment. The PS group represented the positive control group with only β-GP treatment. The data are expressed as the mean ± standard deviation. The data were

analysed with one-way ANOVA with the Bonferroni *post hoc* test or the unpaired, two-tailed Student's t-test. *$p < 0.05$; **$p < 0.01$; ***$p < 0.001$; ****$p < 0.0001$.

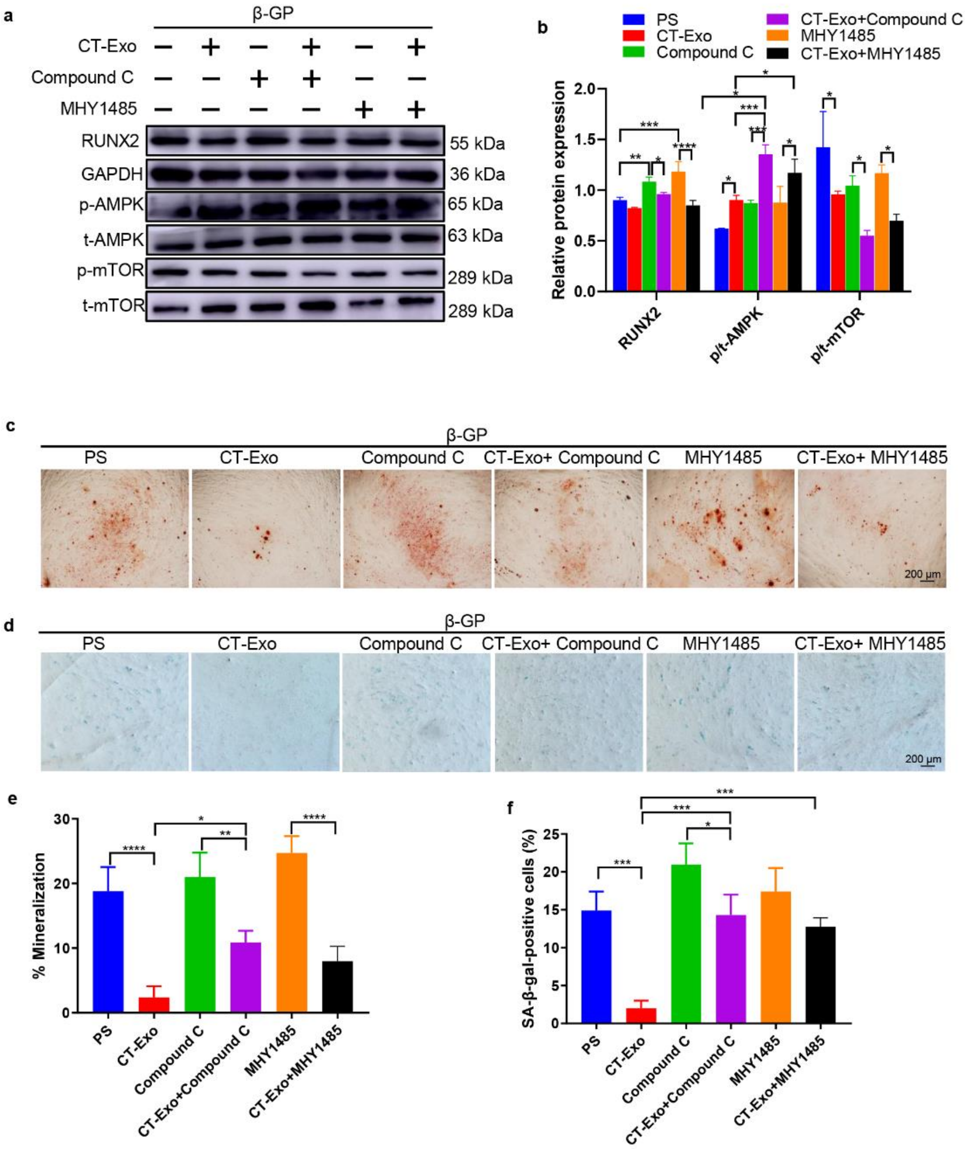

Fig. s8 The AMPK/mTOR signalling pathway mediated defensive roles of CT-Exo on calcification/aging of VSMCs. (a) Expression of p-mTOR and p-AMPK in the β-GP-induced VSMCs treated with Compound C or MHY1485 were analysed by western blot (n = 4 per group). (b) The data are presented as densitometric ratios of RUNX2/GAPDH, p/t-mTOR and p/t-AMPK respectively. (c, d) Representative micrographs of ARS and SA-β-gal staining view were shown (n = 5 per group). (e, f) The data are presented as ratio of positive staining area, shown as the mean ± standard deviation. The data were analysed with one-way ANOVA with the Bonferroni *post hoc* test or the unpaired, two-tailed Student's t-test. *$p < 0.05$; **$p < 0.01$; ***$p < 0.001$; ****$p < 0.0001$.

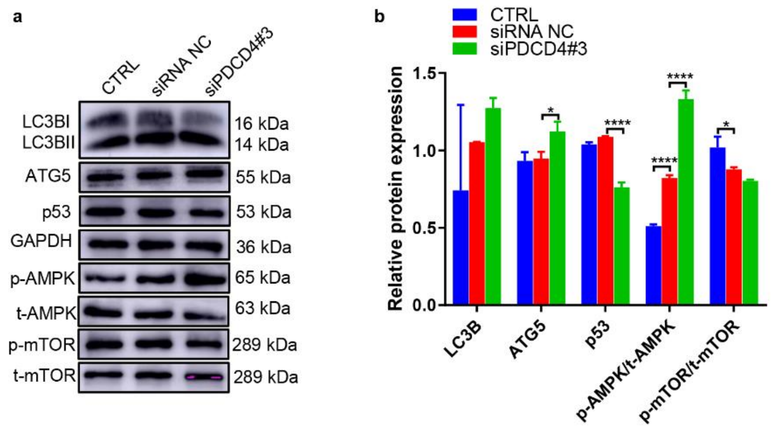

Fig. s9 siPDCD4 can activate the AMPK/mTOR signalling pathway to promote VSMCs autophagy. Western blot analysis (a) and quantification (b) of LC3B, ATG5, p53, p/t-AMPK and p/t-mTOR in VSMCs treated with siPDCD#3 or siRNA control (n = 4 per group). The CTRL group represented the negative control group with only PBS treatment. The data are presented as the mean ± standard deviation. The data were analysed one-way ANOVA with the Bonferroni *post hoc* test. *$p < 0.05$; ****$p < 0.0001$.

Table S1

RNA oligonucleotide sequences.

RNA oligos	Sequences
mimics-NC	Sense: 5'- UUCUCCGAACGUGUCACGUTT -3'<br>Anti-sense: 5'- ACGUGACACGUUCGGAGAATT -3'
inhibitor-NC	Sense: 5'- CAGUACUUUUGUGUAGUACAA -3'
miR-320a-3p mimics	Sense: 5'- AAAAGCUGGGUUGAGAGGGCGA -3'<br>Anti-sense: 5'- GCCCUCUCAACCCAGCUUUUUU -3'
miR-320a-3p inhibitor	Sense: 5'- UCGCCCUCUCAACCCAGCUUUU -3'
agomir-NC	Sense: 5'- UCUCCGAACGUGUCACGUTT -3'<br>Anti-sense: 5'- ACGUGACACGUUCGGAGAATT -3'
Hsa-miR-320a-3p agomir	Sense: 5'- AAAAGCUGGGUUGAGAGGGCGA -3'<br>Anti-sense: 5'- GCCCUCUCAACCCAGCUUUUUU -3'
antagomir-NC	Sense: 5'- CAGUACUUUUGUGUAGUACAA -3'
Hsa-miR-320a-3p antagomir	Sense: 5'- UCGCCCUCUCAACCCAGCUUUU -3'
siRNA-NC	Sense: 5'- UUCUCCGAACGUGUCACGUTT -3'<br>Anti-sense: 5'- ACGUGACACGUUCGGAGAATT -3'
si-PDCD4#1	Sense: 5'- CGCCCUUAGAAGUGGAUUATT -3'

(PDCD4-Homo-533)	Anti-sense: 5'- UAAUCCACUUCUAAGGGCGTT -3'
si-PDCD4#2	Sense: 5'- GGGACAGUAAUGAGCACAATT -3'
(PDCD4-Homo-966)	Anti-sense: 5'- UUGUGCUCAUUACUGUCCCTT -3'
si-PDCD4#3	Sense: 5'- GGAACUGGAAGUACCUCAUTT -3'
(PDCD4-Homo-1340)	Anti-sense: 5'- AUGAGGUACUUCCAGUUCCTT -3'

Table S2

Differentially expressed miRNAs in CT-Exo and RT-Exo

above 1.5-fold change (CT-Exo/RT-Exo)

Systematic Name	Fold Change	P Value
mmu-miR-351-5p	94.14345058	0.001543586
mmu-miR-6538	61.42467529	0.023048967
novel_382	56.48546031	0.014468841
mmu-let-7e-3p	56.30284961	0.025739511
mmu-miR-674-5p	55.09039783	0.016790423
mmu-miR-122-3p	54.47332056	0.047083333
mmu-miR-700-5p	52.84191248	0.037570328
mmu-miR-139-3p	31.77955771	0.031028902
mmu-miR-132-3p	15.52947479	0.001265178
mmu-miR-484	7.101759389	0.020935936
mmu-miR-423-3p	7.100413899	0.00036135
mmu-miR-326-3p	6.075044388	0.029537992
mmu-miR-200b-5p	6.064892227	0.03727916
mmu-miR-361-3p	5.726686309	0.001844238
mmu-miR-1964-3p	5.00455166	0.025783004
mmu-miR-205-5p	4.971517543	0.019277103
mmu-miR-125b-5p	4.732869234	0.00405241
mmu-miR-129b-3p	4.464908874	0.018266228
mmu-miR-129-5p	4.457498999	0.018349179
mmu-miR-25-3p	4.239275669	0.001349913
mmu-miR-3473h-5p	4.136969691	0.033216441
mmu-miR-30f	3.416446609	0.021327587
mmu-miR-541-5p	3.22453363	0.024792296
mmu-miR-1198-5p	3.129684569	0.004581151
mmu-miR-320-3p	2.970331662	0.016394786
mmu-miR-222-3p	2.924196172	0.016064421
mmu-miR-148a-3p	2.572280033	0.014623624
mmu-miR-10a-5p	2.315407853	0.045708164
mmu-miR-22-3p	2.289075786	0.021701031
mmu-miR-486a-3p	2.247090095	0.048455547
mmu-miR-486b-3p	2.233448607	0.049933101
mmu-miR-3074-5p	2.119647107	0.047467127
mmu-miR-30d-5p	2.119353461	0.043618173

below 1/1.5-fold change (CT-Exo/RT-Exo)

Systematic Name	Fold Change	P Value
mmu-miR-21a-5p	0.455624884	0.038703572
mmu-miR-429-3p	0.439845565	0.045975995
mmu-miR-374b-5p	0.418011421	0.036920032
mmu-miR-374c-3p	0.418011421	0.036920032
mmu-miR-455-5p	0.417683968	0.035650318
mmu-miR-20a-5p	0.391811503	0.028183596
mmu-miR-17-5p	0.387886436	0.021436253
mmu-miR-199b-5p	0.382965729	0.044599542
mmu-miR-146a-5p	0.382779982	0.013202416
mmu-miR-499-5p	0.380337471	0.044347646
mmu-miR-574-5p	0.373117938	0.019734011
mmu-miR-199a-3p	0.358798404	0.009561352
mmu-miR-1b-5p	0.356836899	0.039578254
mmu-miR-1a-3p	0.356746148	0.039542379
mmu-miR-106b-5p	0.356702402	0.020516121
mmu-miR-26b-5p	0.350408557	0.007046242
mmu-let-7e-5p	0.349807811	0.014449685
mmu-miR-126a-5p	0.329061765	0.014901413
mmu-miR-126b-3p	0.329061765	0.014901413
mmu-miR-30b-3p	0.31874273	0.009355289
mmu-miR-379-5p	0.300828787	0.002260747
mmu-miR-1839-5p	0.277076994	0.009353765
mmu-let-7g-5p	0.258268459	0.00169423
mmu-miR-18a-5p	0.257652905	0.048768434
mmu-miR-200a-3p	0.253255719	0.002519558
mmu-let-7d-5p	0.252415676	0.002712576
mmu-miR-127-5p	0.250162843	0.040952059
mmu-miR-144-5p	0.246208594	0.001025716
mmu-miR-3071-3p	0.233785141	0.004655797
mmu-miR-7b-5p	0.22772835	0.002973106
mmu-miR-136-5p	0.224000751	0.003643359
mmu-miR-15b-3p	0.197259205	0.000839446
mmu-miR-669a-3p	0.169599839	0.03398022
mmu-miR-206-3p	0.168045084	0.000640255
mmu-miR-7073-5p	0.15249098	0.033920041
mmu-mR-329-3p	0.145051596	0.037213323
mmu-miR-152-3p	0.106641642	8.28E-06
mmu-miR-376b-5p	0.071833174	0.008009447
